# Structural Dynamics Investigation of Human Family 1 & 2 Cystatin-Cathepsin L1 Interaction: A Comparison of Binding Modes

**DOI:** 10.1371/journal.pone.0164970

**Published:** 2016-10-20

**Authors:** Suman Kumar Nandy, Alpana Seal

**Affiliations:** Department of Biochemistry & Biophysics, University of Kalyani, Kalyani, West Bengal, India; Universidade Nova de Lisboa Instituto de Tecnologia Quimica e Biologica, PORTUGAL

## Abstract

Cystatin superfamily is a large group of evolutionarily related proteins involved in numerous physiological activities through their inhibitory activity towards cysteine proteases. Despite sharing the same cystatin fold, and inhibiting cysteine proteases through the same tripartite edge involving highly conserved N-terminal region, L1 and L2 loop; cystatins differ widely in their inhibitory affinity towards C1 family of cysteine proteases and molecular details of these interactions are still elusive. In this study, inhibitory interactions of human family 1 & 2 cystatins with cathepsin L1 are predicted and their stability and viability are verified through protein docking & comparative molecular dynamics. An overall stabilization effect is observed in all cystatins on complex formation. Complexes are mostly dominated by van der Waals interaction but the relative participation of the conserved regions varied extensively. While van der Waals contacts prevail in L1 and L2 loop, N-terminal segment chiefly acts as electrostatic interaction site. In fact the comparative dynamics study points towards the instrumental role of L1 loop in directing the total interaction profile of the complex either towards electrostatic or van der Waals contacts. The key amino acid residues surfaced via interaction energy, hydrogen bonding and solvent accessible surface area analysis for each cystatin-cathepsin L1 complex influence the mode of binding and thus control the diverse inhibitory affinity of cystatins towards cysteine proteases.

## Introduction

Cystatins are typical emergency inhibitors of C1 and C13 family of cysteine proteases (CPs) [1], classified into four groups, namely family 1 or stefins, family 2 or cystatins, family 3 or kininogens and family 4 or cystatins devoid of CP activity [2, 3]. In spite of substantial difference in protein sequence, all members of cystatin superfamily shares the characteristic cystatin fold formed by five-stranded anti-parallel β-sheets (β1-β5) wrapped around a core of a five-turn α-helix (α1) lying almost perpendicular to the sheets (S1 Fig). The connectivity within the cystatin fold is: (N)-β1-α1-β2-L1-β3-(AS)-α2-β4-L2-β5-(C); where AS is a broad “appending structure” positioned at the opposite side relative to the N-terminus and β hairpin loops L1 and L2. Stefins lack AS loop and the second α-helix (α2). Structural studies have recognized three conserved regions in cystatin fold, the exposed L1 loop containing highly conserved (Q-X-V-X-G) region flanked between projecting N-terminal segment and C-terminal L2 loop comprising equally conserved PW segment (with an exception of human stefins), altogether form a tripartite, largely hydrophobic, wedge-shaped edge complementary to the active site of papain-like CPs [4, 5].

But the conservation of tertiary structure or similar mechanism of inhibition [4] does not tally well with the inhibition profile of cystatins. The inhibitory affinities of the human family 1 & 2 cystatins, even towards a particular CP, differ notably [6]. In case of human stefins and cystatins, cystatin C emerges as the best overall inhibitor of C1 family of CPs; whereas SD type of cystatins are the poorest of the lot. Human stefin B, cystatin F appears to be 100 fold poorer inhibitor of cathepsin L in comparison to cystatin C; while stefin A, cystatin M/E are found to be 1000 fold, cystatin SN shows 10^5^ fold and cystatin S & SA exhibits 10^6^ fold lower inhibitory activity [6, 7]. Even in case for human S-type cystatins, where more than 90% sequence similarity is observed, cystatin S comes out as a significantly poorer inhibitor in comparison to cystatin SA & SN [6]. Furthermore, the relative contributions of three conserved regions, viz. N-terminal, L1 & L2 β-hairpin loops, also vary appreciably. In contrast to the previous observations [8], N-terminal segment of human stefin A and B is reported to contribute about 40% of the total free energy of binding for papain, cathepsin B & L [9, 10]; although the first β hairpin loop fails to account for stefin A in CP binding [11] but credited for stefin B-papain interaction [8]; and the second loop of stefin A shows variable affinity depending on the CP [12], responsible for 20–30% of total binding energy in bovine cystatin B-papain, -cathepsin H and–cathepsin B interaction. The L1 and L2 loop of cystatin C is recognized to hold the major share in total free energy of binding of papain, actinidin, cathepsins B and H [13]. For SD-type cystatins, the QXVXG region exhibits main effect on papain inhibition, whereas all three conserved regions participate for cathepsin CPs [6]. Apart from three conserved regions, C-terminal residues also takes part significantly in papain, cathepsin B & H interaction of stefin B [14].

*In vivo* studies further elucidate the considerable correlation of cathepsin B & L activities with the inhibitory activities of stefin A & B in breast carcinoma [15] and head & neck carcinoma [16] patients, respectively. Substantial reduction in stefin B inhibitory activity and concomitant increase in cathepsins S and L activity is observed in EPM1 pathogenesis [17]. Various reports suggests the levels of cystatin C [18], cystatin M/E [19] and stefin A [20] in tissue and extracellular fluids can serve as relatively reliable markers for diagnosis and prognosis of a variety of diseases [1, 21] while cathepsin B & L levels may well be used as a potential indicator of tumor aggressiveness and metastasis [22, 23].

In this paper the detailed molecular mechanisms behind the diverse inhibitory interaction of human family 1 & 2 cystatins, namely stefin A, B & cystatin C, D, F, M/E, S, SA, SN, with cathepsin L1 (CL1) were investigated. Firstly, the CL1-cystatin/stefin complexes were built through molecular docking and then atomistic molecular dynamics studies of the binding mode of all nine complexes were performed. The integrity of the complexes were verified, important residues involved in interaction were identified by interaction energy (IE), solvent accessible surface area (SASA), and hydrogen bond (HB) analysis and in turn correlated with the varied contributions of the conserved regions of cystatins involved in binding. Altogether our results explained the nature of interaction and variable affinity of cystatins towards CL1.

## Methods

### Prediction of intrinsic disordered region

The amino acid sequences of human CL1 and all nine cystatins were retrieved from UniProt and were subjected to DisEMBL 1.5 [24], Globplot 2.3 [25], regional order neural network (RONN) [26] and protein disorder prediction system (PrDOS) [27] to specify regions of higher flexibility and complementarity.

### PDB file preparation

Initial atomic coordinates of six proteins, to wit CL1, stefin A, stefin B, cystatin C, cystatin D, and cystatin F were selected on basis of active/inhibitory site mutants, incomplete chains, missing residues, domain swapping etc. from the Research Collaboratory for Structural Bioinformatics Protein Data Bank (www.pdb.org) and downloaded accordingly [28]. Crystal waters were removed, atoms were reordered in certain residues, terminals were modified and conformers (if any) were deleted on basis of occupancies and further the occupancies were corrected. Then crystal structures were subjected to Swiss PDB Viewer [29], mainly to clear the nomenclature issues. The specific information on file preparation is given in S1 Table. For the structures of cystatin M/E, cystatin S, cystatin SA and cystatin SN comparative models prepared previously [30] were used.

### Complex formation—molecular docking

CASTp (Computed Atlas of Surface Topology of proteins) [31], cons-PPISP (consensus–Protein–Protein Interaction Site Predictor) [32] and the InterProSurf server [33] were utilized to locate the binding-sites in prepared PDB files. To find out the binding modes of cathepsin-cystatin/stefin complexes, crystallographic and modeled structures of stefin A, B, cystatin C, D, F, M/E, S, SA & SN were docked with CL1 using protein-protein rigid body docking program ZDOCK [34] of Discovery Studio (DS) 2.5. The active site of CL1 (CYS 25, HIS 163, ASN 187) [UniProt ID: P07711] and the possible inhibitory sites of cystatins [6, 35] were defined to screen the potential docking configurations. A total of 2000 docked poses were generated for each of the cystatin/stefin-cathepsin complexes and ranked based on the default ZDOCK scoring function [36, 37] combining shape complementarities with electrostatics and desolvation energy (DE), and subsequently re-ranked with a more detailed weighted energy function ZRANK [38]. On basis of known cathepsin-cystatin/stefin conformations, binding site root mean square deviation (RMSD) were calculated and all docked poses within 7Å deviation were subjected to RDOCK [39] for further refinement. The best docking pose with the lowest RDOCK energy was considered.

### Molecular dynamics simulations

In total, 27 systems were prepared for simulation. For each of the nine CL1-cystatin/stefin complexes, we simulated (a) the complex of the CL1 and its binding partner, (b) the CL1 alone, and (c) the binding partner alone. The initial coordinates of simulation groups (b) and (c) were taken directly from group (a). All molecular dynamics simulations (MDS) were performed using GROMACS (Groningen Machine for Chemical Simulations) 4.5.4 [40] simulation packages employing Charmm27 (Chemistry at Harvard Macromolecular Mechanics) force field [41]. The systems were solvated by explicit SPC/E (extended simple point charge) water model [42] in cubic boxes maintaining a minimum 10 Å edge distance. Counter ions (Cl^-^ and Na^+^) were added by randomly replacing water molecules to achieve a neutral simulation cell of physiological ionic strength (0.10 molar). Composition of each system was given in S2 Table. All systems were then minimized using a steepest descents (SD) algorithm [43] in order to eliminate steric clashes or inappropriate geometry.

A 100 ps NVT equilibration was performed at 300 K with position restraints applied to all of the backbone atoms to ease any bad contacts at the side chain solvent interface. The velocity rescale thermostat [44] was used with a temperature coupling time constant (τ_t_) of 0.1 ps.

All bond lengths were constrained using the linear constraint solver (LINCS) [45] algorithm allowing for a 2 fs time step. Long-range electrostatic interactions were approximated using the particle mesh Ewald (PME) method [46] with a fourth-order spline interpolation and a 0.15 nm Fourier grid spacing. The short range non-bonded interactions were defined as van der Waals (VDW) and electrostatic interactions for particles within 10 Å. Then all position restraints were withdrawn and a 100 ps NPT simulation was conducted; an isotropic Parrinello-Rahman barostat [47] was set to 1.0 bar of pressure in all directions with a pressure coupling time constant (τ_p_) of 2.0 ps. Afterwards 150 ns production run was followed using velocity rescale thermostat and Parrinello-Rahman barostat; LINCS and PME treatments were also implemented as described. Snapshots of the trajectory were taken in every 2 ps.

The GROMACS suite of tools along with a secondary structure recognition algorithm DSSP (Define Secondary Structure of Proteins) [48] and VMD [49] was used for all types of MDS analysis. Every complex trajectory was subjected to gromos clustering and the middle most structure of the largest cluster was taken as representative structure (RS) and compared with the final docking output of same complex (DC) to assess the effect of refinement. Principal component analysis (PCA) was used to track the collective motions of bound and unbound inhibitors. Further, the change in solvent-accessible surface area (ΔSASA), HB pattern and IEs of each residue in the binding interface were determined. Microsoft Excel program was used for preparation of the graph and DS visualizer was employed for 3-D figure generation.

## Results and Discussion

All the intrinsic disorder predicting servers detected the N- and C-terminal region of all ten proteins as disordered regions. Additionally the regions near chain break (residues 172–179) and various loop regions (residues 11–25, 54–69, 80–109, 154–161) of CL1 were recognized as disordered region. Further, AS loop of cystatin C, D & M/E was noticed as disordered region in RONN; Globplot & RONN spotted part of β4 of cystatin C and D; in all S-type cystatins, β4, α2 and β2 was noted as disordered regions by RONN and DisEMBL respectively; in addition DisEMBL marked β3 of cystatin SN as highly disordered loops.

In the following sub-sections, we first checked the consistency among nine simulations of unbound CL1, then stability of the all nine complexes were assessed, the effect of refinement was quantified and binding modes of complexes were compared in view of diverse participation of conserved regions in VDW and electrostatic interactions. The detailed analyses of all nine individual CL1-stefin/cystatin complexes were placed in S1 Text. The unbound CL1s were named after the corresponding CL1-stefin/cystatin complex (say, CL1_A_ denotes the unbound CL1 taken from CL1-Stefin A complex) from which its initial coordinates were taken as stated in MDS section under Methods. The amino acid residues of stefins/cystatins were referred according to their positions in the alignment (S2 Fig) and for CL1 the crystal structure (PDB ID: 1ICF) numbering was followed.

### Consistency of simulations: unbound cathepsins

**Fig 1:**
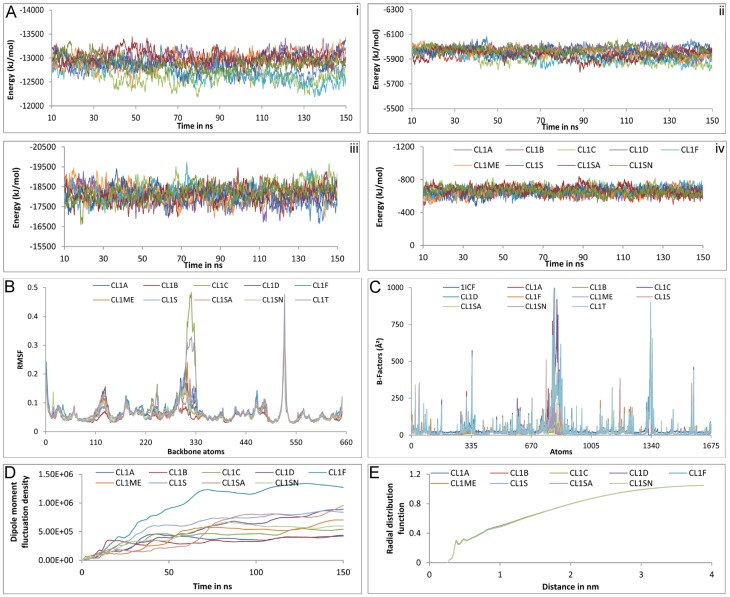
Convergence of the properties of un-bound cathepsin L1s in solution. A. Non-bonded electrostatic (i, iii) and VDW (ii, iv) energy profile in intra-protein (i, ii) and protein-water (iii, iv) interfaces. Color code is same for i-iv. B. RMSF analysis. C. Crystallographic and simulated B-factors. D. Protein dipole moment fluctuation densities. E. Radial distribution functions between backbone atoms of protein and water oxygen atoms.

All nine simulations of unbound CL1 were appeared to be in stable state in terms of intra-protein, protein-solvent electrostatic and VDW energy (Fig 1A). Electrostatic energy profile exhibited more fluctuation than the VDW one, protein-water interface recorded higher variation compared to intra-protein electrostatics.

Although average structures might not be physically meaningful or energetically accessible but can be used as a tool for characterizing sampling. Here a true representative average structure of every trajectory was computed by an iterative procedure; average structure was calculated using the first frame and then repeated with the resulting average structure until the RMSD between successive average structure becomes <0.001 Å [50]. The individual trajectory-average structures of CL1 differed from the crystal structure by 0.873–1.033 Å, compared to a 0.272–0.419 Å difference with respect to the global trajectory-average; while they differed from each other by 0.399–0.639 Å. S3 Table showed, the individual trajectory-averages of unbound CL1s (CL1_A_—CL1_SN_) were closer to the global trajectory average (CL1_GT_) than the crystal structure (PDB ID: 1ICF). Little variation among these average structures pointed towards convergence of simulation and indicated toward possible overlap of conformational spaces between the trajectories [51]. In fact PCA depicts an overlap of 0.327–0.614 among individual trajectories and 0.449–0.643 between individual and global trajectories (S4 Table). Stable RMSD output and atomic motions of unbound receptors also seconded the same by exhibiting similar nature of fluctuation (S1 Text and Fig 1B). Discrepancies observed in the residues 70–80 and 103–108 of unbound cathepsin of cystatin C complex, which also contributed at global trajectory (CL1_GT_), were already predicted as disordered region.

Structural properties of the unbound cathepsins illustrated little variation in individual trajectories and resembled more with the global trajectory rather the crystal structure (S5 Table). The molecular volume, density and radius of gyration (Rg) recorded minute variation of 0.3–0.6%, 0.4–0.5% & 0.4–1% with relative to the crystal structure and 0.4%, 0.4% & 0.2–0.4% in case of global trajectory, respectively. Only noticeable change was observed in case of SASA, 7.4–10.6% in comparison to crystal structure and 0.8–2.1% for global trajectory. This might be due to difference in environment between solvent simulation and crystallographic structure determination. In contrast, to all these structural characteristics crystallographic and simulated B-factors hardly correlated with each other, simulated ones reported markedly higher values (Fig 1C). This might be due to huge difference in determination techniques, time-scales and environment [52].

Dielectric relaxation of unbound CL1s was considered through dipole moments fluctuation density, which were stabilized around 90 ns although CL1F described distinctly higher values (Fig 1D). Hydration of CL1 surface defined by radial distribution functions were seemed to be indistinguishable from each other (Fig 1E). Thus we can say the non-bonded energies, conformational differences, dielectric relaxation, hydration of protein and most of the structural properties except B-factor looked to be converged within the limit of the simulation.

### Stability of the complexes

**Fig 2:**
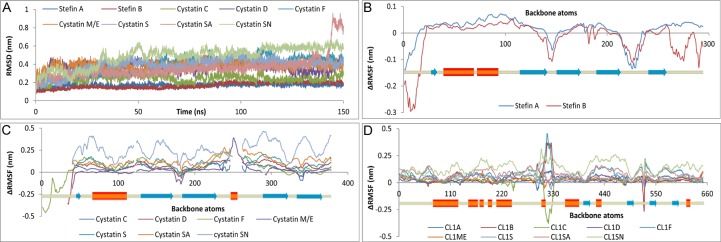
Stability of the complexes. RMSD of cathepsin L1-stefin/cystatin complexes (A) and ΔRMSF (RMSF_bound_—RMSF_unbound_) in stefins (B), cystatins (C) and cathepsin L1s (D).

DSSP profile demonstrated overall stability of secondary structural elements in receptor and inhibitor in both bound and unbound state over the period of simulation; recording only a maximum change of 3% in secondary structural elements between bound and unbound proteins (S1 Text). RMSD analysis also depicted by and large stability of all complexes—stefin A, B and cystatin C, D & SA complexes got stabilized readily within 10–20 ns whereas cystatin F, M/E, S & SN complexes illustrated high relaxation time of ≈60 ns (Fig 2A). ΔRMSF study (ΔRMSF = RMSFbound—RMSF_unbound_) recorded mostly higher fluctuation of the bound proteins with comparatively lower fluctuation at the sites of direct interaction (Fig 2B–2D). Apart from the global observations some local attributes were also noticed, such as marked stabilization of β-hairpin loops in bound state especially in case of stefin A, B and cystatin C complexes (Fig 2B and 2C and S3A, S3B, S7A, S7B, S11A and S11B Figs); split of β5 into β-sheet & β-bridge in bound cystatin D leads to higher RMSF at C-terminal (Fig 2C and S15A and S16B Figs) and presence of 2^nd^ α-helix in all cystatins but cystatin M/E were observed both in bound and unbound state (S1 Text). The four residue insertions in the AS loop of cystatin M/E (S2 Fig), also recognized as disordered region, might be the reason behind disappearance of α2, as well of its unique appearance in DSSP analysis, and higher fluctuation in RMSF analysis (Fig 2C, S23A, S23B and S24B Figs). CL1 illustrated little difference between bound and unbound states except near the region of chain break (residue 175) and the loop region (residues 95–113), both noted as disordered segment (Fig 2D). These two regions were also registered high RMSF values and contributed in RMSD plot of CL1s associated with stefin B, cystatin C, F, S, SN complexes and unbound receptors (Fig 2A and 2D and S8A, S8C, S12A, S12C, S20A, S20C, S28A, S28C, S36A and S36C Figs). Cystatin C, D, M/E and SN complexes exhibited more variation in RMSD profile, CL1-Stefin B complex reported an increase in RMSD near 75ns and again stabilized ≈100 ns; cystatin SA complex also illustrated an increase in RMSD in post 130ns simulation; in the final 35 ns cystatin SN reported almost a plateau (Fig 2A). The peculiarities of the complex RMSD were gathered either from the receptor or the inhibitor, such as bound receptor of stefin B complex showed a similar increase in RMSD after 75 ns due to enhanced fluctuation of disordered loop residues 99–112 (Fig 2A and S8A and S8C Fig); markedly higher fluctuation in AS loop was responsible for late increase in RMSD in cystatin SA complex (Fig 2A and S32B Fig); while higher RMSF of the bound inhibitors were accountable for the RMSD fluctuations of cystatin C (S12A and S12B Fig), M/E (S24A and S24B Fig—AS loop) and higher RMSF of both bound receptor and inhibitor were liable for RMSD fluctuations of cystatin D (S16 Fig), SN (S36 Fig). The bound CL1s and stefins/cystatins portrayed either lower or comparable RMSD with the unbound one. In case the bound ligand reported higher RMSD values compared to the unbound ligand, the bound receptor registered lower RMSD compared to the unbound CL1 and vice-versa–thus illustrating the stability of the complexes (S1 Text). Further the Rg and total SASA served as additional measure of stability of the complexes recording only ≈2% variation (S6 Table).

### Quantification of refinement by MDS

Each of the CL1-stefin/cystatin complex trajectories was subjected to gromos clustering [53] and the middle most structure of the largest cluster was taken as representative structure (RS) and compared with the final docking output of same complex (DC) to quantify the effects of refinement through MDS.

Comparison of secondary structure of RSs with corresponding DCs revealed certain regions of the receptor were transformed into helix (residues 58–60), while in other regions helices (residues 64–66, 102–104) and sheets (83–84, 105–107) were altered into turn and coils as an effect of refinement. In case of inhibitors, after refinement, various secondary structure modifications were observed in 1^st^ β-sheet (sheet structure shifted towards N-terminal in Cystatin M/E & SA, reappeared in cystatin SN, shortened in Cystatin C & D); 2^nd^ and 3^rd^ β-sheet were shortened in cystatin F, M/E, SN and cystatin M/E, S respectively; elongation of 5^th^ β-sheet was seen in stefin complexes, while the same was broken into two parts in cystatin C & D complexes and got shortened in cystatin M/E and SA (S7 Table).

**Fig 3:**
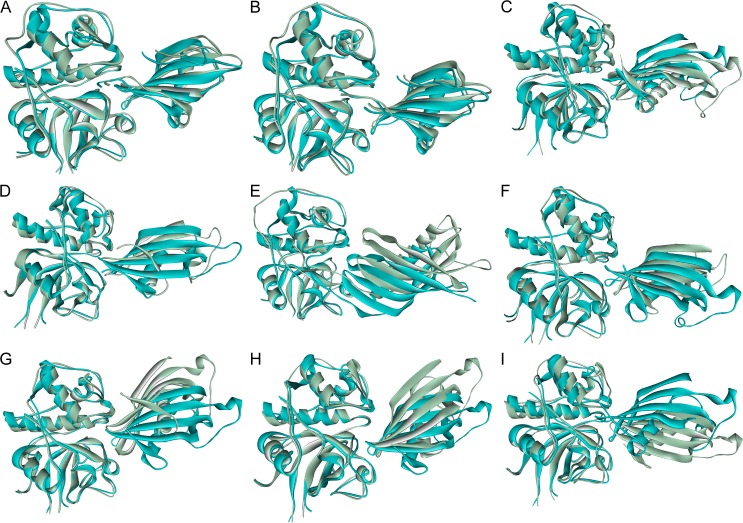
Change in conformation of complexes after refinement. Superimposed structures of refined complexes and corresponding docking outputs of cathepsin L1 complexes with (A) stefin A, (B) stefin B, (C) cystatin C, (D) cystatin D, (E) cystatin F, (F) cystatin M/E, (G) cystatin S, (H) cystatin SA, (I) cystatin SN. Refined structure was shown in green and docked output in cyan.

Superposition of receptors of RS with equivalent DC illustrated differences in binding conformations of the inhibitors after MDS; shift of inhibitor centroids, relative motion between secondary structures were observed on refinement, Cystatin F and M/E recorded the highest RMSD (Fig 3 and S8 Table). Depending upon the degree of changes implicated on refinement, the binding interface of the DC & RS differs accordingly; cystatin D, F and SN showed the most number of alterations (S9 Table). The RS of stefin A moved a bit far from the receptor along horizontal axis, shift of centroids by 2.64 Å, in comparison to the docked complex (Fig 3A and S8 Table); which enables the refined complex to identify the importance of Pro3 [9] of stefin A and Asn66 of CL1 in molecular interaction and HB forming that remained unrecognized in the docking studies (Table 1, S1 Text and S6 Fig). Refined Stefin B exhibited least deviation and centroid motion (Fig 3B, S8 Table) but good enough to reveal the crucial roles of Met1, Ala49, His75 of the inhibitor [10, 14] and Gly68 of CL1 in hydrogen bonding, electrostatic and VDW interaction (Table 1, S1 Text and S10 Fig). In DCs of stefin A & B, catalytic site residue Cys25 of CL1 was predicted to form HB with stefins, but was lost on refinement—in agreement with the exosite nature of stefins. Cystatin C was tilted and centroid was repositioned in its refined form–the N-terminal and L1 loop came up from the docking plane whereas the L2 loop goes down due to clockwise (β2, β4, β5) and anti-clockwise (β1, β3) motions of β-sheets (Fig 3C and S8 Table). As a result the HB between Gly67 and Asp162 was the only HB of DC that retained after refinement and Gly68 of CL1, N-terminal Gly19, entire L1 loop residues of cystatin C specially Ile64 except Lys62 emerges as new interaction centre (Table 1, S1 Text and S14 Fig). RS of cystatin D also took an inclined conformation in comparison to the DC, undergone a large diagonal shift in YZ plane, while β-hairpin loops of both structures remained almost fixed, the N-terminal & far end of RS (C-terminal, AS loop) moved away with respect to docked conformation (Fig 3D and S8 Table). The electrostatic, VDW interaction and hydrogen bonding pattern remained almost same except the N-terminal participation got reduced and contribution of two β-hairpin loops amplified after refinement (Table 1, S1 Text and S18 Fig). In cystatin F DC, mainly the N-terminal along with two β-hairpin loops blocked the catalytic site of the receptor; while in the RS, the role of N-terminal remained almost same but L1 & L2 loops moved away from the catalytic core (Fig 3E and S8 Table). Different conformations of N-terminal and counter rotations of β-hairpin loop forming sheets of RS have contributed to the repositioning of cystatin centroids; different conformations of terminal regions and AS loop as well recorded the highest deviation among refined cystatins (S8 Table). Correspondingly the interactions with L1 and L2 loop got diminished; Thr6 of cystatin F and Glu63, Asn66 of receptor came into the interaction sphere (Table 1, S9 Table, S1 Text and S22 Fig). All three conserved sites of cystatin M/E stayed in almost same position before and after refinement, although the refined inhibitor undergoes an anti-clockwise rotation for which the far end of the refined inhibitor and L2 loop moved away from the docked position (Fig 3F). A shift of centroid was observed, L1 loop of RS popped up the docking plane due to counter rotations of β2 & β3 sheets (S8 Table). Two HBs between L2 loop and receptor residues were lost and rest of the interaction pattern remain roughly the same (Table 1 and S9 Table, S1 Text and S26 Fig). Cystatin S got pulled towards the receptor on refinement—documented second highest centroid movement; rotated anti-clockwise by keeping the point of interaction of L1 loop almost same, while L2 loop dipped below the docking plane due to counter rotations of β4 & β5 and N-terminal moved off from the receptor (Fig 3G and S8 Table). Interacting residues remained more or less identical before and after MDS (S9 Table) but their involvement in interaction changed a lot; after refinement, HB pattern got altered totally, L1 loop residues dominated the interaction (Table 1, S1 Text and S30 Fig). Position of three conserved site remained almost stationary for cystatin SA before and after refinement but the far end of the inhibitor (C-terminal, AS loop) came closer the receptor as in case of cystatin S but reported a lower centroid shift (Fig 3H and S8 Table). Residue association in electrostatic & VDW interaction stayed nearly same but lot of short lived HBs formed with L2 loop of the inhibitor on refinement owing to its sinking below the docking plane due to counter rotations of β4 & β5 (Table 1, S8 and S9 Table, S1 Text and S34 Fig). RS of cystatin SN moved away from the receptor rotated in clockwise direction, accounted for the highest centroid shift, portraying the reverse scenario of cystatin S & SA (Fig 3I and S8 Table). Consequently N-terminal region acted as the principal interaction hub and number of interacting residues reduced heavily from the β-turns (Table 1, S9 Table, S1 Text and S38 Fig).

**Table 1:** Key residues of interaction.

Cathepsin L1 complex with	Receptor	Inhibitor
Stefin A	Gly20, ***Gln21***, *Gly23*, Cys65, ***Asn66***, Gly68, Glu141, ***Leu144***, *Phe14*5, *Met161*, ***Asp162***, ***Trp189***	***Met1***, *Ile2*, ***Pro3***, ***Gly4***, *Gln46*, ***Val47***, ***Val48***, ***Ala49***, *Gly50*, *Phe70*, ***Lys71***, *Leu73*, *Pro74*
Stefin B	Gly20, ***Gln21***, *Gly23*, **Cys65**, ***Asn66***, ***Gly68***, *Leu69*, Met70, **Asp71**, *Leu144*, *Phe145*, Tyr146, *Met161*, ***Asp 162***, ***Trp 189***, *Glu192*	***Met1***, ***Met2***, ***Cys3***, ***Gly4***, *Pro6*, *Gln46*, ***Val48***, ***Ala49***, *Gly50*, Asn52, *Phe70*, *Gln71*, *Ser72*, *Leu73*, ***His75***, *Lys78*, Ser83, ***Tyr 97***
Cystatin C	Gln60, *Glu63*, *Asn66*, ***Gly68***, *Leu69*, *Gly139*, *His140*, *Leu144*, ***Asp162***	***Gly19***, *Pro20*, ***Gln63***, ***Ile64***, ***Val65***, ***Ala66***, **Gly67**, *Tyr115*, *Pro118*, *Trp119*
Cystatin D	*Glu63*, ***Asn66***, **Gly68**, *Leu69*, *Gly139*, Asp160, ***Asp162***	*Gly18*, *Gln63*, ***Ile64***, ***Val65***, ***Gly66***, Asn69, Gln113, Asn115, ***Trp119***
Cystatin F	*Gln21*, **Glu63**, *Cys65*, ***Asn66***, *Leu69*, ***Asp 71***, *Tyr72*, *Gln78*, **Asp114**, *Gly139*, *His140*, **Glu159**, ***Asp160***, ***Met161***, *Asp162*, **Ala214**, **Ser216**	***Thr6***, *Cys7*, *Ser13*, ***Arg14***, ***Lys16***, **Lys66**, ***Trp119***
Cystatin M/E	*Gln60*, ***Glu63***, ***Asn66***, ***Gly68***, *Leu69*, *Gly139*, *Leu144*, **Glu159**, ***Asp160***, **Met161**, ***Asp162***	***Arg21***, *Gln29*, *Leu64*, ***Val65***, ***Ala66***, ***Gly67***, *Ile68*, ***Lys69***, *Pro118*, ***Trp119***
Cystatin S	*Glu63*, ***Asn66***, Gly68, *Leu69*, *Gly139*, *Leu144*, *Asp162*, *Trp189*	*Gln63*, ***Phe65***, ***Gly66***, ***Asn69***, *Phe71*, *Glu113*, *Pro118*, ***Trp119***, Asn127
Cystatin SA	***Gln21***, **Glu63**, ***Asn66***, *Gly139*, *Leu144*, **Asp160**, ***Asp162***, *Glu192*	***Gly19***, Gln63, *Ile64*, ***Val65***, *Gly66*, Gln113, *Tyr115*, ***Trp119***, Arg122, Ser124, *Val126*, Asn127, **Arg129**
Cystatin SN	*Gln21*, *Asn66*, *Tyr72*, **Asp137**, *Gly139*, **Asp160**, ***Asp162***	***Gly19***, ***Tyr21***, *Val65*, Gly66, Tyr115, ***Trp119***

Residues that reports high IE (>30KJ/mol) are in bold, hydrogen bonded residues are underlined and residues marked in *SASA analysis* are *italicized*.

### Comparative study of the interaction profile of complexes

#### Cathepsin L1

**Fig 4:**
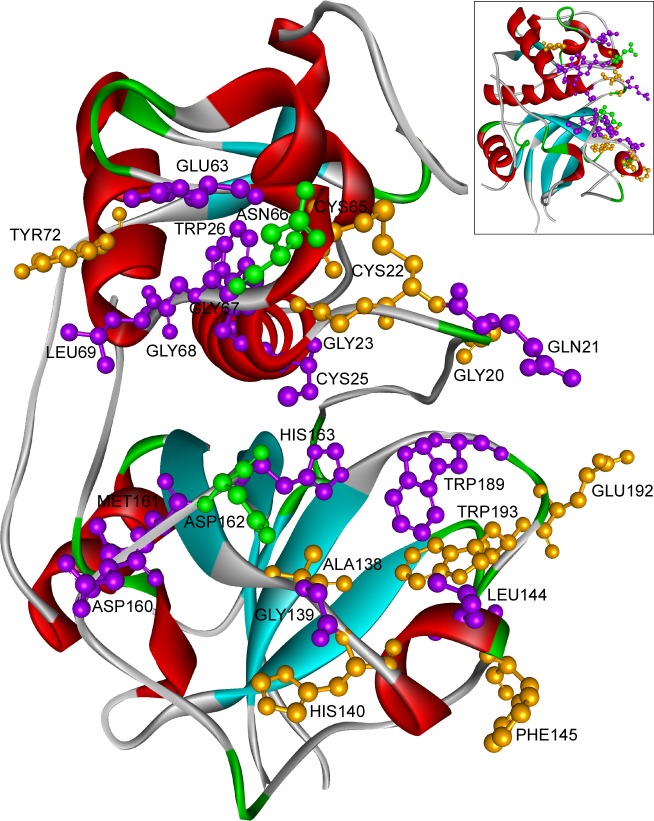
Participation of cathepsin L1 residues in cathepsin L1-stefin/cystatin interaction. Residues always involve in interaction were colored in green, mostly took part were in purple and occurred seldom were in orange. cathepsin L1 (inset) and its 90˚ rotated were shown in picture. Residues with IE >10KJ/mol were considered for the representation.

Our observations goes well with the current knowledge that human family 1 and 2 cystatins does not directly bind to the catalytic site residues of CL1 (Cys25, His163, Asn187), rather blocks the access to these residues by binding in nearby positions (Table 1). The CL1 residues were portrayed in Fig 4 according to their frequency of participation in CL1-stefin/cystatin interaction. We found four distinct regions of CL1 that contributed heavily in the interaction, to wit

1. residues Gln21;
2. residues Glu63, Asn66, Gly68, Leu69;
3. residues Gly139, Leu144; and
4. residues Asp160, Asp162 (Table 1).

The first group was more important for stefins; the third group of residues mainly participated in VDW contacts; the residues of second and fourth group contributed to both electrostatic and VDW interaction. In stefin interaction few more residues took part, such as Gly20, Gly23, Cys65, Phe145, Met161 and Trp189. Among all these residues Asn66 and Asp162 came common in all the complexes, participated in all kind of interaction with high IE as well (Table 1).

#### Cystatins

Among all nine CL1-cystatin complexes, VDW interaction dominated the IE profile in five complexes (Stefin A, B, Cystatin C, D and S), electrostatic interaction did so in three (Cystatin F, M/E and SN) and in cystatin SA-CL1 complex, electrostatic and VDW contacts were of equal importance (Table 2). Correlated motions always remained largely associated with the conserved region that dominated the overall interaction of any particular complex. Out of three conserved regions, the conserved L2 loop served as a VDW interaction centre; generally provided 10–30% of total potential energy of interaction to cystatins and 20–25% for stefins with two marked exceptions of cystatin S and SA, where L2 loop hold a larger share, 42% and 53% of IE, respectively. The highly conserved PW motif (S2 Fig) provided 50–90% of the total L2 loop contribution, while Trp alone contributed 40–75%. Cystatin SA-CL1 complex came up as an exception, where PW motif provided only 20% of the L2 loop IE and the rest amount was contributed by the adjacent β4, β5 sheet residues (S34A Fig). In stefins, as the conserved Trp remained absent Pro alone contributed 12–15% of IE, comparable to the cystatin complexes.

**Table 2:** Relative contribution of conserved regions and their participation in electrostatic and van der Waals interaction.

Cathepsin L1 complex with	Contribution in Interaction Energy (IE) in %				IE (E:V)
	N-terminal	L1 Loop	L2 Loop	C-terminal	
Stefin A	39	35	24	2	28:72
E:V	26:74	23:77	37:63	49:51	
Stefin B	45	23	21	11	40:60
E:V	48:52	25:75	33:67	52:48	
Cystatin C	19	68	13	-	32:68
E:V	42:58	33:67	16:84		
Cystatin D	6	61	33	-	32:68
E:V	33:67	34:66	28:72		
Cystatin F	73	17	10	-	73:27
E:V	78:22	87:13	13:87		
Cystatin M/E	27	55	18	-	61:39
E:V	100:0	61.5:38.5	4:96		
Cystatin S	6	52	42	-	21.5:78.5
E:V	60:40	34:66	1:99		
Cystatin SA	26	21	53	-	53:47
E:V	99:1	25:75	41:59		
Cystatin SN	67	19	14	-	69:31
E:V	93.5:6.5	17:83	20:80		

E- Electrostatic Interaction & V–van der Waals Interaction

**Fig 5:**
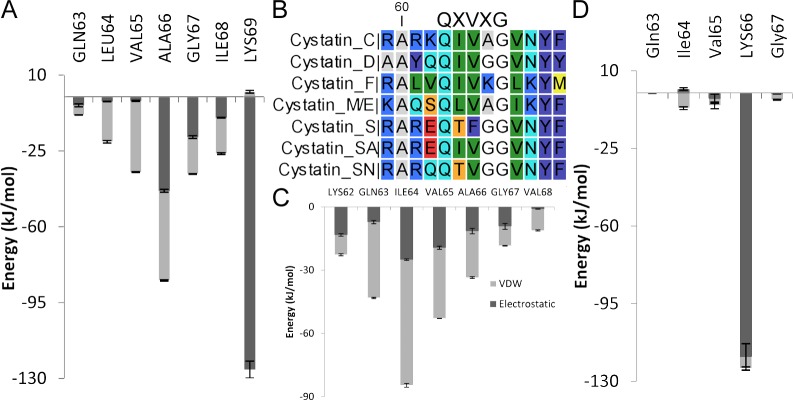
Contribution of L1 loop in cathepsin L1-cystatin interaction. Interaction energy profile of L1 loop of cystatin M/E (A), cystatin C (C), cystatin F (D) and alignment of L1 loop residues of cystatins (B). Error bars represent the estimated error in GROMACS calculation. Legends were same in A, C, D.

L1 loop seemed to be of major importance for Cystatin C, D, M/E & S, where it provided 52–68% of total IE, for the rest the share varied between 17–35% (Table 2). L1 loop region in general acted as VDW interaction centre for stefin/cystatin complexes, 65–83% of L1 loop contribution was provided by VDW contacts, while cystatin F and M/E complexes emerged as exceptions. The third and fourth residue of the QXVXG motif looked to be of more importance, provided 50–60% of the L1 loop contribution; for cystatin F the contribution became highest (93% of L1 loop IE) and reached the low for cystatin C & M/E (30.7% & 35.4% respectively). All hydrophobic residues in QIVAG contributed in cystatin C and the most hydrophobic second Ile residue played the central role (Fig 5C)–nullifies the usual dominance of 3^rd^ & 4^th^ residue. CL1-cystatin C complex also recorded the highest L1 loop contribution (68% of IE) with the help of strongest hydrophobic triplet IVA among the cystatins (Table 2 and Fig 5B). The presence of Lys residue within the QXVXG motif (Fig 5D) of cystatin F disrupted the hydrophobic core and as a result VDW contribution, merely turned into 13% of L1 loop’s share of IE. Only one residue, Lys66, recorded high IE in the IE profile of cystatin F (Fig 5D), in contrast to regular presence of 2–3 residues in IE profile form L1 loop with a high score and noted change in SASA. In case of cystatin M/E the residue Lys69, showed high electrostatic IE, formed multiple HBs, and influenced the interaction of L1 loop in a great extent (Fig 5A and S26B Fig). Although the hydrophobic core and the regular involvement of QXVXG persisted, due to the presence of more hydrophobic residues Ala66 & Ile68 in the region (Fig 5B), but the overall interaction profile of L1 loop (Fig 5A) moved into the electrostatic way (61.5% of L1 loop contribution).

Role of N-terminal also varied widely among the complexes, while it provided ≈40% of IE to the stefins, the contribution of this region became as low as 6% for cystatin D & S complexes, turned moderate (19–27% of total IE) for cystatin C, M/E & SA and recorded highest for cystatin F and SN complexes, 73% & 67% of total IE, respectively (Table 2). Mostly N-terminal acted as an electrostatic interaction centre, only in cystatin C, D and stefin A, VDW interaction prevailed; for stefin B electrostatic and VDW contacts were equally important. The C-terminal also shared equal contribution of electrostatic and VDW interaction but appeared to be of significance for stefin-CL1 complexes only.

**Fig 6:**
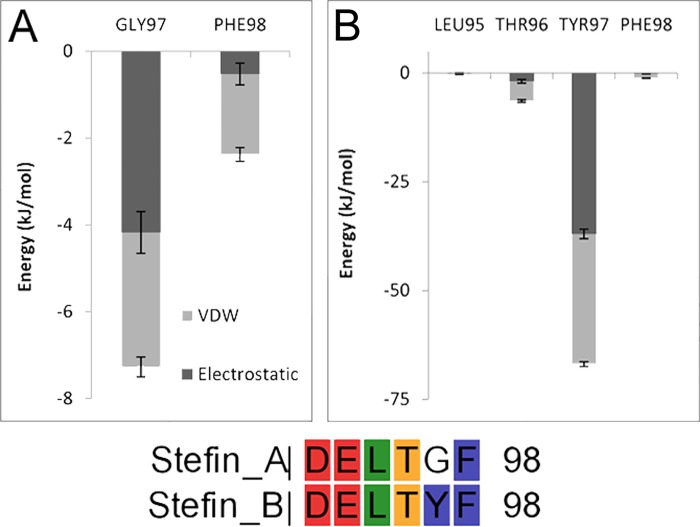
C-terminal interaction profile of stefin-cathepsin L1 complexes. Potential energy of interaction of C-terminal region of Stefin A (A) and Stefin B (B) along with their C-terminal alignment.

Further similarities were noticed between the interaction profiles of the complexes, which also portrayed diverse approaches of cystatins towards CL1. Depending on parallel roles of inhibitor and receptor residues, similarities in IE profile and diverse contributions of conserved regions, stefin/cystatin–CL1 complexes might be grouped into stefin A, B; cystatin C, M/E; cystatin D, S and cystatin F, SN, while cystatin SA stood alone. This differential interaction patterns most likely had been the reason of varying affinity of cystatins towards cathepsins along with the dissimilar amino acid compositions at three interacting sites. For example, presence of Gly instead Tyr (residue 97) was the obvious reason behind minor contribution of C-terminal of stefin A compared to stefin B-CL1 complex (Table 2 and Fig 6); similarly occurrence of more hydrophobic Ala at 66^th^ position helped cystatin C and M/E to establish hydrophobic contacts more efficiently than others (Fig 5A–5C), which also contributed to the L1 loop participation (Table 2) & CL1 affinity as well; consecutive presence of less hydrophobic Thr and Phe at L1 loop (Fig 5B, 64^th^ & 65^th^ position) of cystatin S might be the reason of its inactive nature; differential affinity of S-type of cystatins in spite of their huge sequence similarity might be attributed to their extremely diverse interaction profile (S30A, S34A and S38A Figs), varying contribution of conserved regions (Table 2).

## Conclusions

In this work, inhibitory complexes of CL1 and human family 1 and 2 cystatins were modeled; built complexes were verified with available experimental data [5, 9, 10, 12, 13, 14, 54] and used for further study of molecular interactions between receptor and inhibitor. Cystatins were exosite binding inhibitors of CL1, interacted with the receptor through their three conserved regions in more than one way as documented by their interaction profiles. While the CL1-cystatin F, M/E, SN complexes were dominated by electrostatic interaction, the interaction profile of cystatin SA was equipoised between electrostatic and VDW contacts and in the rest, VDW interaction prevailed. L1 and L2 loop mainly acted as VDW interaction centre, whereas electrostatic interaction prevailed in N-terminal. In fact, L1-loop dictated the balance of the overall interaction profile, whether it will be dominated by VDW contacts or electrostatic interaction will play the key role. The little differences in amino acid compositions in the respective conserved sites controlled the diverse interaction patterns of cystatins, which in turn determined the wide-ranging affinity of cystatins towards cathepsins. These results were in tune with the available information and explained the nature of interactions & variable affinities of cystatins as well. Our findings will be helpful in understanding the details of cystatin inhibition and will serve as a guideline for mutation & protein designing studies.

## Supporting Information

S1 Fig Structure and connectivity of cystatin fold. The prepared human cystatin C (S1 Table) was used for visualization.
(TIF)

S2 Fig Alignment of human family 1 and 2 cystatins. Disulfide bonds and conserved regions involved in CP inhibition were marked. Alignment was performed in Clustal X2 [55] with default parameters and visualized by CLC sequence viewer 7 (http://www.clcbio.com/products/clc-sequence-viewer/).
(TIF)

S3 Fig DSSP analysis of Stefin A–Cathepsin L1 complex. Secondary structure content of Stefin A in bound (A) and unbound (B) state and that of Cathepsin L1 in bound (C) and unbound (D) form.
(TIF)

S4 Fig RMSD & RMSF analyses for Stefin A-Cathepsin L1 complex. (A) Average backbone RMSD of complex, inhibitor (I) and receptor (R) in bound and unbound state. RMSF of Stefin A (B) and Cathepsin L1 (C) in complex and in absence of their binding partner.
(TIF)

S5 Fig PCA of Stefin A-Cathepsin L1 complex. (A) Covariance matrix illustrating correlated and anticorrelated motions of bound (top left) and unbound (bottom right) stefin A. The secondary structure of stefin A backbone is represented along the axes (from left to right and from bottom to top). (b) Motion of the largest eigenvector of stefin A in absence (left) and presence (right) of cathepsin L1.
(TIF)

S6 Fig Binding interface characterization of Stefin A-Cathepsin L1 complex. (A) Potential energy of interaction between binding interface residues of stefin A (I) & cathepsin L1 (R). Error bars represent the estimated error in GROMACS calculation. (B) Average number of HBs formed among interface residues; error bars designate standard deviation. (C) Appreciable changes in SASA on complex formation among binding interface residues.
(TIF)

S7 Fig DSSP analysis of Stefin B–Cathepsin L1 complex. Secondary structure content of Stefin B in bound (A) and unbound (B) state and that of Cathepsin L1 in bound (C) and unbound (D) form.
(TIF)

S8 Fig RMSD & RMSF analyses for Stefin B-Cathepsin L1 complex. (A) Average backbone RMSD of the complex, inhibitor (I) and receptor (R) in bound and unbound state. RMSF of Stefin B (B) and Cathepsin L1 (C) in complexed form and in free state in solution.
(TIF)

S9 Fig PCA of Stefin B-Cathepsin L1 complex. (A) Covariance matrix illustrating correlated and anticorrelated motions of bound (top left) and unbound (bottom right) stefin B. The secondary structure of stefin B backbone is represented along the axes (from left to right and from bottom to top). (b) Motion of the largest eigenvector of stefin B in absence (left) and presence (right) of cathepsin L1.
(TIF)

S10 Fig Binding interface characterization of Stefin B-Cathepsin L1 complex. (A) Potential energy of interaction between binding interface residues of stefin B (I) & cathepsin L1 (R). Error bars represent the estimated error in GROMACS calculation. (B) Average number of HBs formed among interface residues. Error bars designate standard deviation. (C) Appreciable changes in SASA on complex formation among binding interface residues.
(TIF)

S11 Fig DSSP analysis of Cystatin C–Cathepsin L1 complex. Secondary structure content of cystatin C in bound (A) and unbound (B) state and that of cathepsin L1 in bound (C) and unbound (D) form.
(TIF)

S12 Fig RMSD & RMSF analyses for Cystatin C-Cathepsin L1 complex. (A) Average backbone RMSD of the complex, inhibitor (I) and receptor (R) in bound and unbound state. RMSF of cystatin C (B) and cathepsin L1 (C) in complexed form and in free state in solution.
(TIF)

S13 Fig PCA of Cystatin C-Cathepsin L1 complex. (A) Covariance matrix illustrating correlated and anticorrelated motions of bound (top left) and unbound (bottom right) cystatin C. The secondary structure of cystatin C backbone is represented along the axes (from left to right and from bottom to top). (b) Motion of the largest eigenvector of cystatin C in absence (left) and presence (right) of CL1.
(TIF)

S14 Fig Binding interface characterization of Cystatin C-Cathepsin L1 complex. (A) Potential energy of interaction between binding interface residues of cystatin C (I) & cathepsin L1 (R). Error bars represent the estimated error in GROMACS calculation. (B) Average number of HBs formed among interface residues. Error bars designate standard deviation. (C) Appreciable changes in SASA on complex formation among binding interface residues.
(TIF)

S15 Fig DSSP analysis of Cystatin D–Cathepsin L1 complex. Secondary structure content of cystatin D in bound (A) and unbound (B) state & that of cathepsin L1 in bound (C) and unbound (D) form.
(TIF)

S16 Fig RMSD & RMSF analyses for Cystatin D-Cathepsin L1 complex. (A) Average backbone RMSD of the complex, inhibitor (I) and receptor (R) in bound and unbound state. RMSF of cystatin D (B) and cathepsin L1 (C) in complexed form and in free state in solution.
(TIF)

S17 Fig PCA of Cystatin D-Cathepsin L1 complex. (A) Covariance matrix illustrating correlated and anticorrelated motions of bound (top left) and unbound (bottom right) cystatin D. The secondary structure of cystatin D backbone is represented along the axes (from left to right and from bottom to top). (b) Motion of the largest eigenvector of cystatin D in absence (left) and presence (right) of cathepsin L1.
(TIF)

S18 Fig Binding interface characterization of Cystatin D-Cathepsin L1 complex. (A) Potential energy of interaction between binding interface residues of cystatin D (I) & cathepsin L1 (R). Error bars represent the estimated error in GROMACS calculation. (B) Average number of HBs formed among interface residues. Error bars designate standard deviation. (C) Appreciable changes in SASA on complex formation among binding interface residues.
(TIF)

S19 Fig DSSP analysis of Cystatin F–Cathepsin L1 complex. Secondary structure content of cystatin F in bound (A) and unbound (B) state & that of cathepsin L1 in bound (C) and unbound (D) form.
(TIF)

S20 Fig RMSD & RMSF analyses for Cystatin F-Cathepsin L1 complex. (A) Average backbone RMSD of the complex, inhibitor (I) and receptor (R) in bound and unbound state. RMSF of cystatin F (B) and cathepsin L1 (C) in complexed form and in free state in solution.
(TIF)

S21 Fig PCA of Cystatin F-Cathepsin L1 complex. (A) Covariance matrix illustrating correlated and anticorrelated motions of bound (top left) and unbound (bottom right) cystatin F. The secondary structure of cystatin F backbone is represented along the axes (from left to right and from bottom to top). (b) Motion of the largest eigenvector of cystatin F in absence (left) and presence (right) of cathepsin L1.
(TIF)

S22 Fig Binding interface characterization of Cystatin F-Cathepsin L1 complex. (A) Potential energy of interaction between binding interface residues of cystatin F (I) & cathepsin L1 (R). Error bars represent the estimated error in GROMACS calculation. (B) Average number of HBs formed among interface residues. Error bars designate standard deviation. (C) Appreciable changes in SASA on complex formation among binding interface residues.
(TIF)

S23 Fig DSSP analysis of Cystatin M/E–Cathepsin L1 complex. Secondary structure content of cystatin M/E in bound (A) and unbound (B) state and that of cathepsin L1 in bound (C) and unbound (D) form.
(TIF)

S24 Fig RMSD & RMSF analyses for Cystatin M/E-Cathepsin L1 complex. (A) Average backbone RMSD of the complex, inhibitor (I) and receptor (R) in bound and unbound state. RMSF of cystatin M/E (B) and cathepsin L1 (C) in complexed form and in free state in solution.
(TIF)

S25 Fig PCA of Cystatin M/E-Cathepsin L1 complex. (A) Covariance matrix illustrating correlated and anticorrelated motions of bound (top left) and unbound (bottom right) cystatin M/E. The secondary structure of cystatin M/E backbone is represented along the axes (from left to right and from bottom to top). (b) Motion of the largest eigenvector of cystatin M/E in absence (left) and presence (right) of cathepsin L1.
(TIF)

S26 Fig Binding interface characterization of Cystatin M/E-Cathepsin L1 complex. (A) Potential energy of interaction between binding interface residues of cystatin M/E (I) & cathepsin L1 (R). Error bars represent the estimated error in GROMACS calculation. (B) Average number of HBs formed among interface residues. Error bars designate standard deviation. (C) Appreciable changes in SASA on complex formation among binding interface residues.
(TIF)

S27 Fig DSSP analysis of Cystatin S–Cathepsin L1 complex. Secondary structure content of cystatin S in bound (A) and unbound (B) state & that of cathepsin L1 in bound (C) and unbound (D) form.
(TIF)

S28 Fig RMSD & RMSF analyses for Cystatin S-Cathepsin L1 complex. (A) Average backbone RMSD of the complex, inhibitor (I) and receptor (R) in bound and unbound state. RMSF of cystatin S (B) and cathepsin L1 (C) in complexed form and in free state in solution.
(TIF)

S29 Fig PCA of Cystatin S-Cathepsin L1 complex. (A) Covariance matrix illustrating correlated and anticorrelated motions of bound (top left) and unbound (bottom right) cystatin S. The secondary structure of cystatin S backbone is represented along the axes (from left to right and from bottom to top). (b) Motion of the largest eigenvector of cystatin S in absence (left) and presence (right) of cathepsin L1.
(TIF)

S30 Fig Binding interface characterization of Cystatin S-Cathepsin L1 complex. (A) Potential energy of interaction between binding interface residues of cystatin S (I) & cathepsin L1 (R). Error bars represent the estimated error in GROMACS calculation. (B) Average number of HBs formed among interface residues. Error bars designate standard deviation. (C) Appreciable changes in SASA on complex formation among binding interface residues.
(TIF)

S31 Fig DSSP analysis of Cystatin SA–Cathepsin L1 complex. Secondary structure content of cystatin SA in bound (A) and unbound (B) state and that of cathepsin L1 in bound (C) and unbound (D) form.
(TIF)

S32 Fig RMSD & RMSF analyses for Cystatin SA-Cathepsin L1 complex. (A) Average backbone RMSD of the complex, inhibitor (I) and receptor (R) in bound and unbound state. RMSF of cystatin SA (B) and cathepsin L1 (C) in complexed form and in free state in solution.
(TIF)

S33 Fig PCA of Cystatin SA-Cathepsin L1 complex. (A) Covariance matrix illustrating correlated and anticorrelated motions of bound (top left) and unbound (bottom right) cystatin SA. The secondary structure of cystatin SA backbone is represented along the axes (from left to right and from bottom to top). (b) Motion of the largest eigenvector of cystatin SA in absence (left) and presence (right) of cathepsin L1.
(TIF)

S34 Fig Binding interface characterization of Cystatin SA-Cathepsin L1 complex. (A) Potential energy of interaction between binding interface residues of cystatin SA (I) & cathepsin L1 (R). Error bars represent the estimated error in GROMACS calculation. (B) Average number of HBs formed among interface residues. Error bars designate standard deviation. (C) Appreciable changes in SASA on complex formation among binding interface residues.
(TIF)

S35 Fig DSSP analysis of Cystatin SN–Cathepsin L1 complex. Secondary structure content of cystatin SN in bound (A) and unbound (B) state & that of cathepsin L1 in bound (C) and unbound (D) form.
(TIF)

S36 Fig RMSD & RMSF analyses for Cystatin SN-Cathepsin L1 complex. (A) Average backbone RMSD of the complex, inhibitor (I) and receptor (R) in bound and unbound state. RMSF of cystatin SN (B) and cathepsin L1 (C) in complexed form and in free state in solution.
(TIF)

S37 Fig PCA of Cystatin SN-Cathepsin L1 complex. (A) Covariance matrix illustrating correlated and anticorrelated motions of bound (top left) and unbound (bottom right) cystatin SN. The secondary structure of cystatin SN backbone is represented along the axes (from left to right and from bottom to top). (b) Motion of the largest eigenvector of cystatin SN in absence (left) and presence (right) of cathepsin L1.
(TIF)

S38 Fig Binding interface characterization of Cystatin SN-Cathepsin L1 complex. (A) Potential energy of interaction between binding interface residues of cystatin SN (I) & cathepsin L1 (R). Error bars represent the estimated error in GROMACS calculation. (B) Average number of HBs formed among interface residues. Error bars designate standard deviation. (C) Appreciable changes in SASA on complex formation among binding interface residues.
(TIF)

S1 Table Structure files preparation. (DOCX)

S2 Table Composition of simulated systems. (DOCX)

S3 Table Pairwise RMSD (Å) of the crystal structure (1ICF), individual trajectory-averages (CL1A—CL1SN) and global trajectory-average structure (GT) using mainchain atoms. (DOCX)

S4 Table Conformational space normalized overlaps between cathepsin L1 (CL1). (DOCX)

S5 Table Structural Properties of unbound CL1s. (DOCX)

S6 Table Structural properties of complexes as a measure of stability. (DOCX)

S7 Table Changes in secondary structure after refinement through MDS. (DOCX)

S8 Table Reciprocal arrangements of cystatins in Docked & Refined structures. (DOCX)

S9 Table Changes in Binding Interface (BI). (DOCX)

S10 Table Flexibility of bound and unbound cystatins. (DOCX)

S1 Text Simulation results of Cathepsin L1-Stefin/Cystatin complexes. (DOCX)
