# Supplementary figures and images for: Structural Dynamics Investigation of Human Family 1 & 2 Cystatin-Cathepsin L1 Interaction: A Comparison of Binding Modes
Source: PLoS One. 2016 Oct 20;11(10):e0164970. doi: 10.1371/journal.pone.0164970 (PMC5072729; doi:10.1371/journal.pone.0164970)

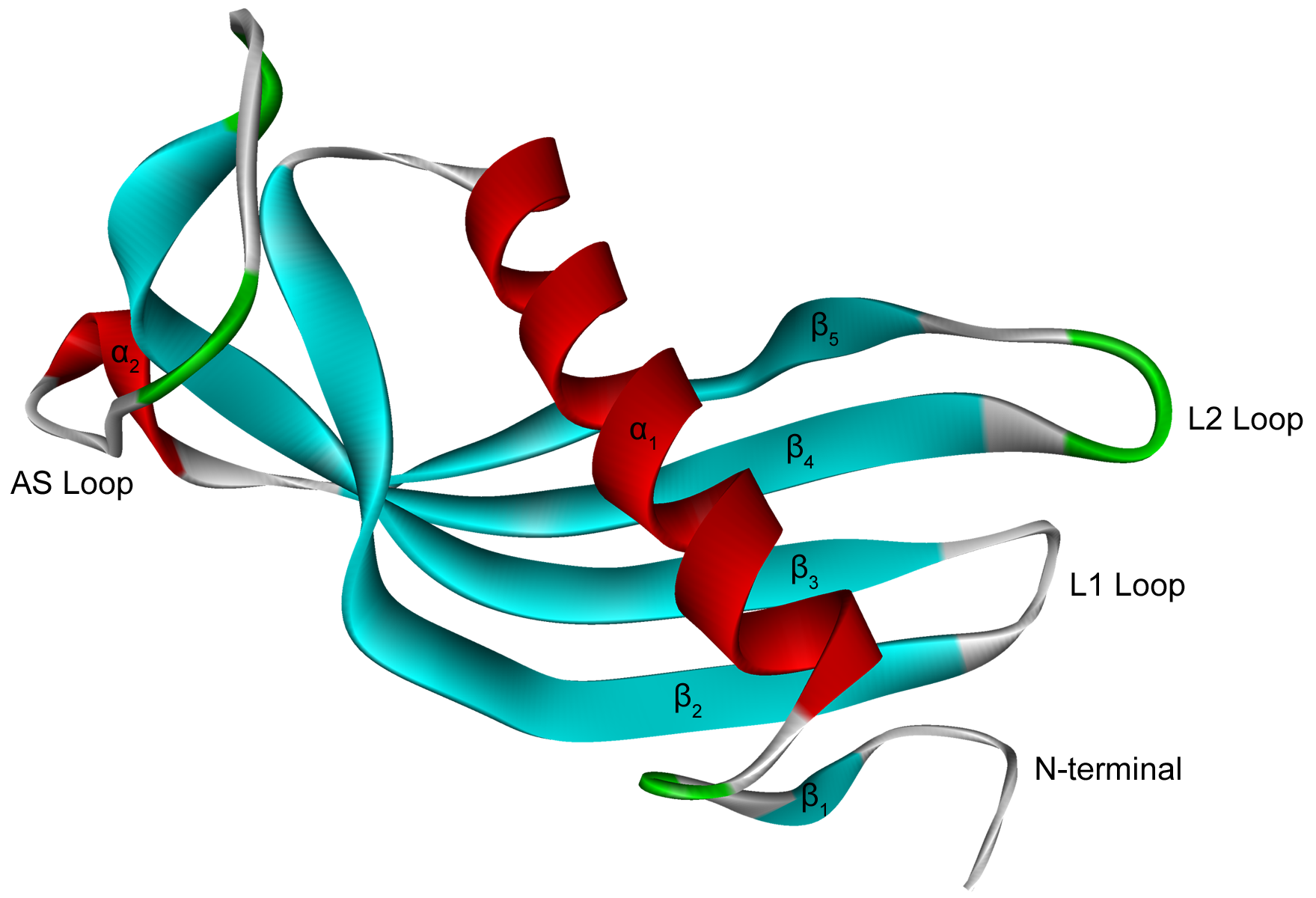

Supplement: S1 Fig — The prepared human cystatin C (S1 Table) was used for visualization. (TIF) [file pone.0164970.s001.tif]

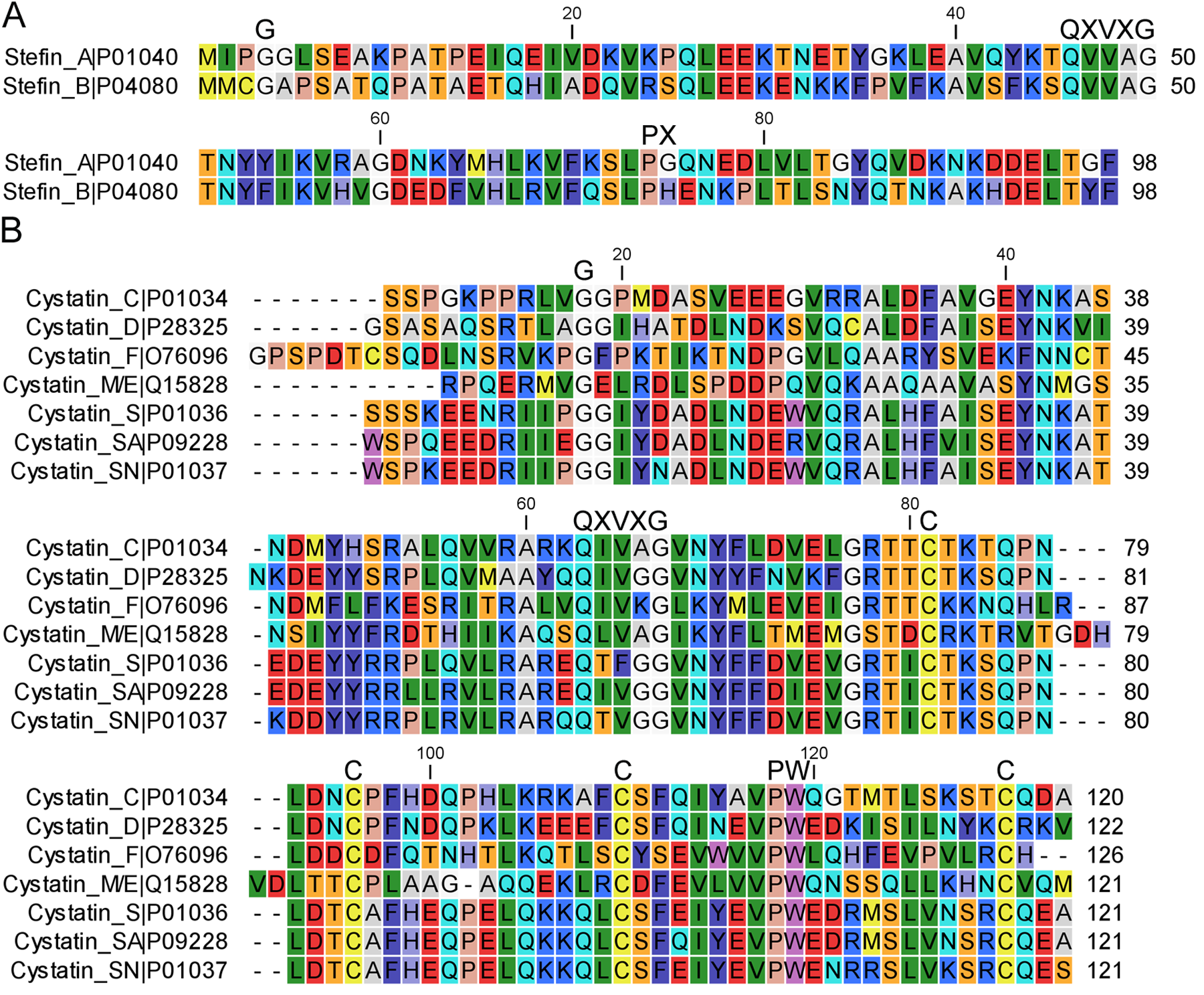

Supplement: S2 Fig — Disulfide bonds and conserved regions involved in CP inhibition were marked. Alignment was performed in Clustal X2 [55] with default parameters and visualized by CLC sequence viewer 7 (http://www.clcbio.com/products/clc-sequence-viewer/). (TIF) [file pone.0164970.s002.tif]

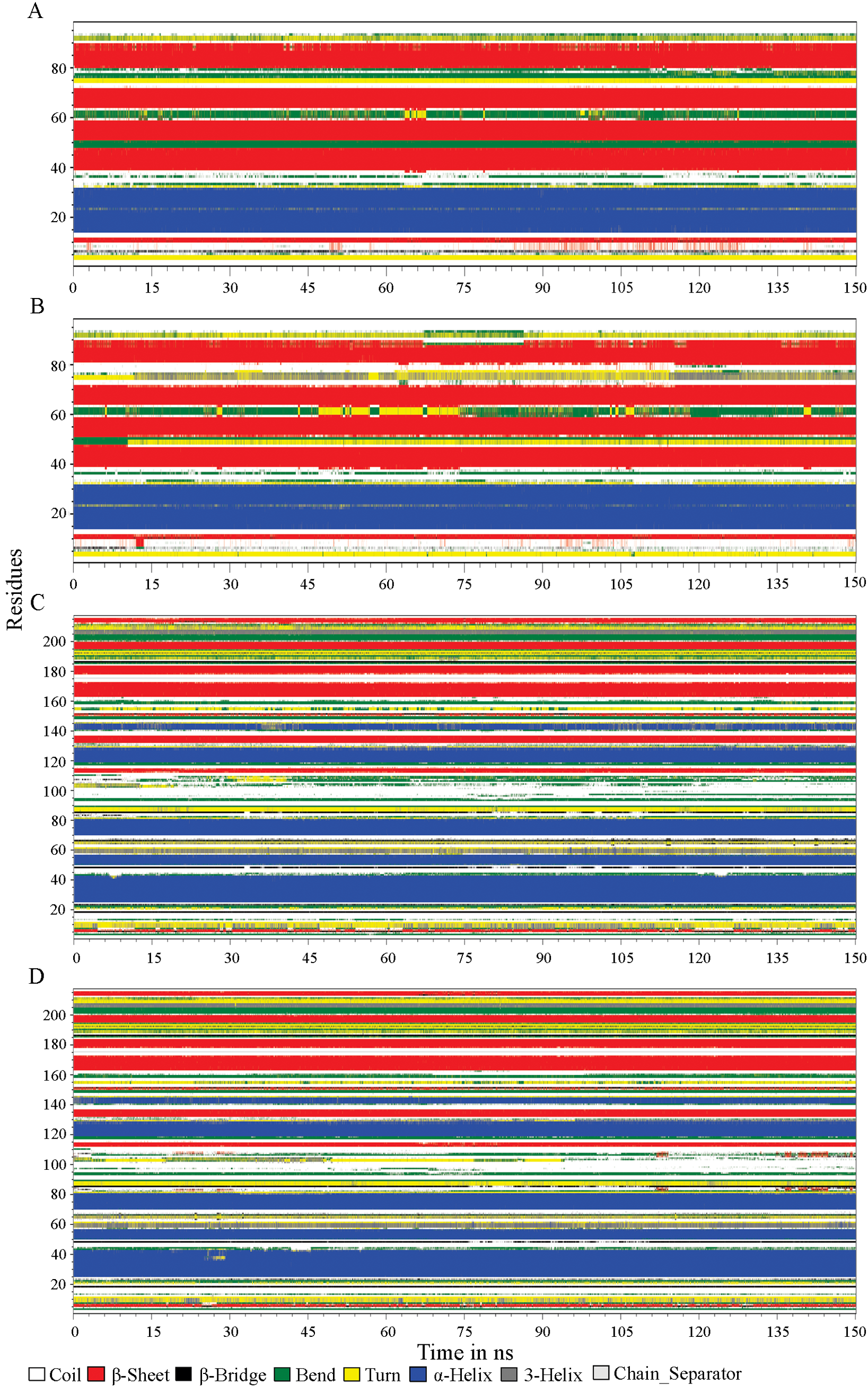

Supplement: S3 Fig — Secondary structure content of Stefin A in bound (A) and unbound (B) state and that of Cathepsin L1 in bound (C) and unbound (D) form. (TIF) [file pone.0164970.s003.tif]

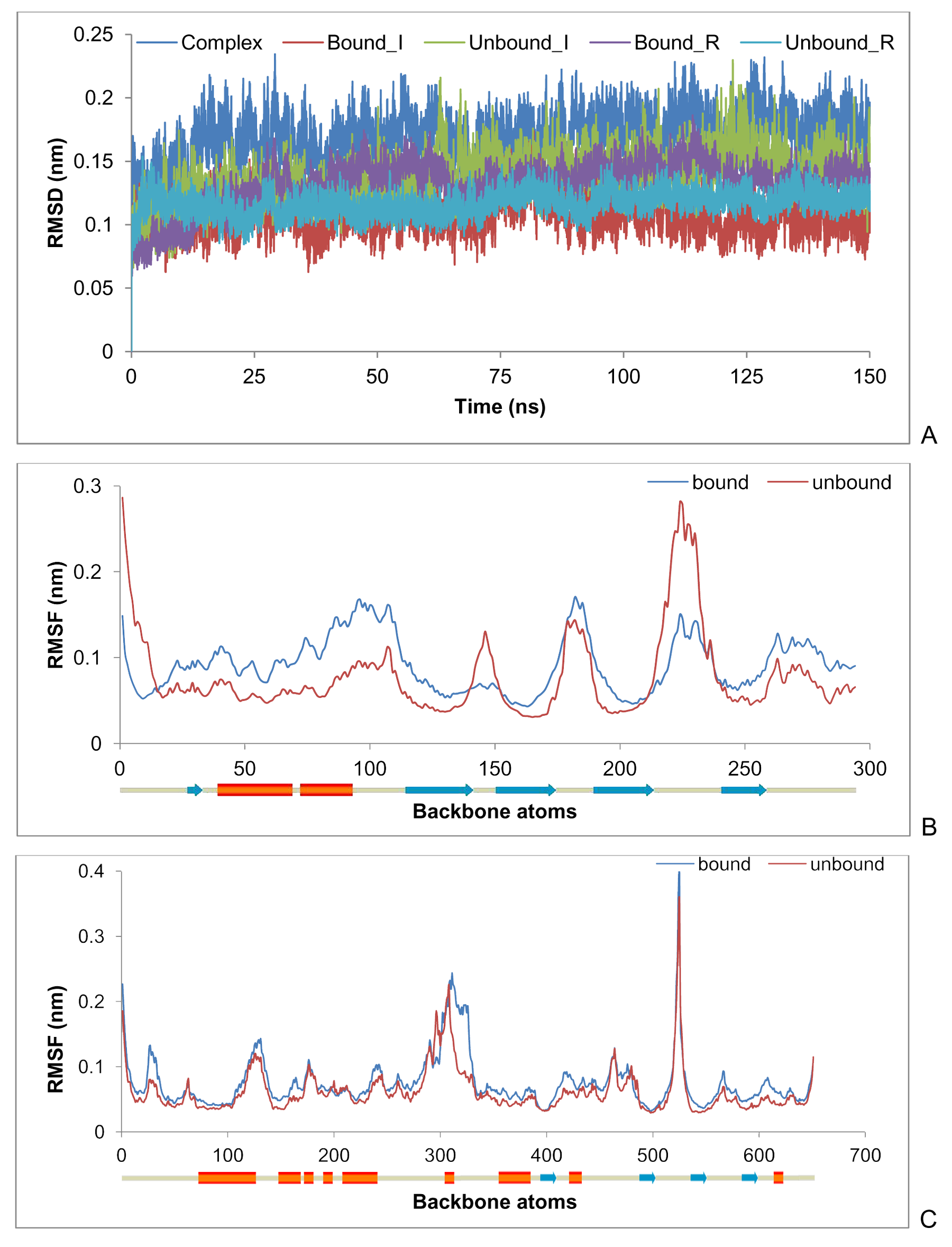

Supplement: S4 Fig — (A) Average backbone RMSD of complex, inhibitor (I) and receptor (R) in bound and unbound state. RMSF of Stefin A (B) and Cathepsin L1 (C) in complex and in absence of their binding partner. (TIF) [file pone.0164970.s004.tif]

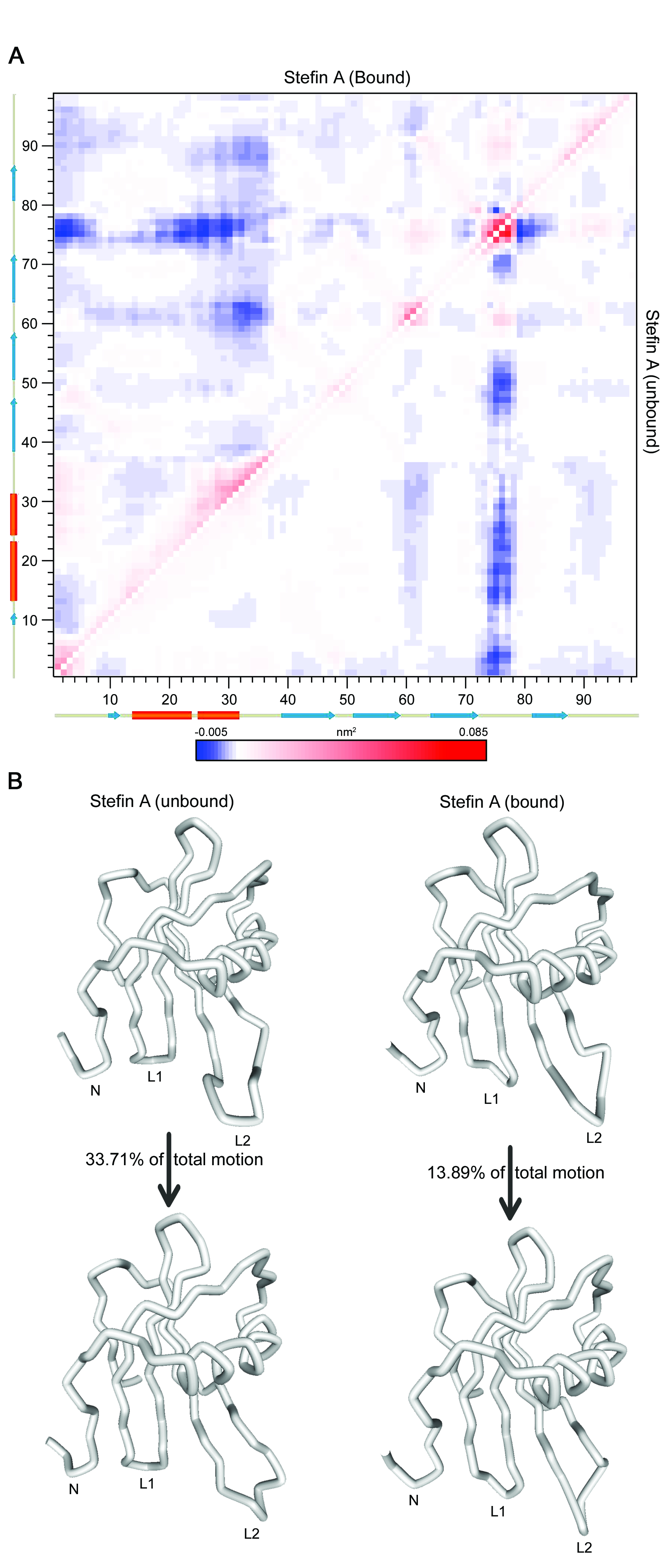

Supplement: S5 Fig — (A) Covariance matrix illustrating correlated and anticorrelated motions of bound (top left) and unbound (bottom right) stefin A. The secondary structure of stefin A backbone is represented along the axes (from left to right and from bottom to top). (b) Motion of the largest eigenvector of stefin A in absence (left) and presence (right) of cathepsin L1. (TIF) [file pone.0164970.s005.tif]

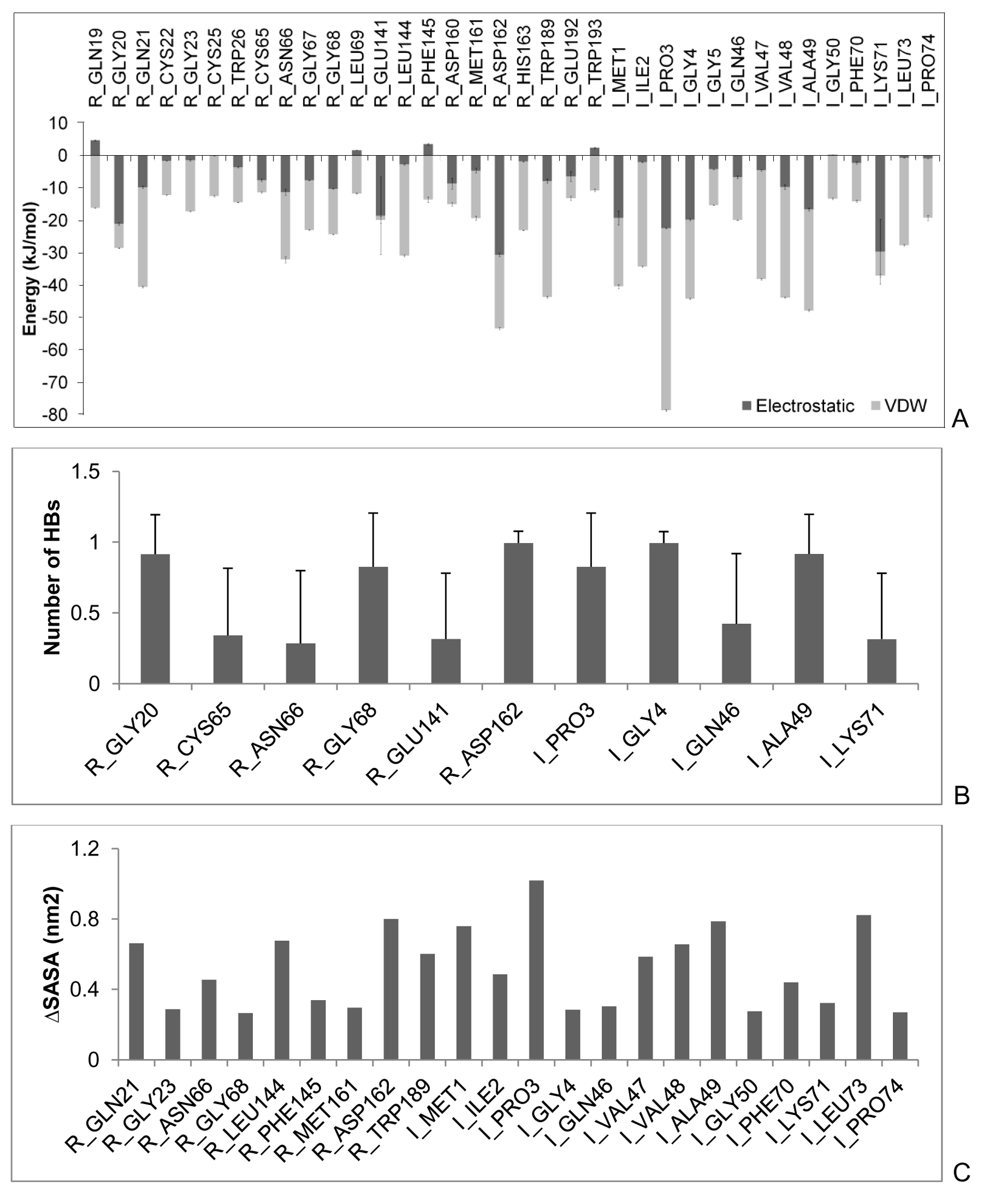

Supplement: S6 Fig — (A) Potential energy of interaction between binding interface residues of stefin A (I) & cathepsin L1 (R). Error bars represent the estimated error in GROMACS calculation. (B) Average number of HBs formed among interface residues; error bars designate standard deviation. (C) Appreciable changes in SASA on complex formation among binding interface residues. (TIF) [file pone.0164970.s006.tif]

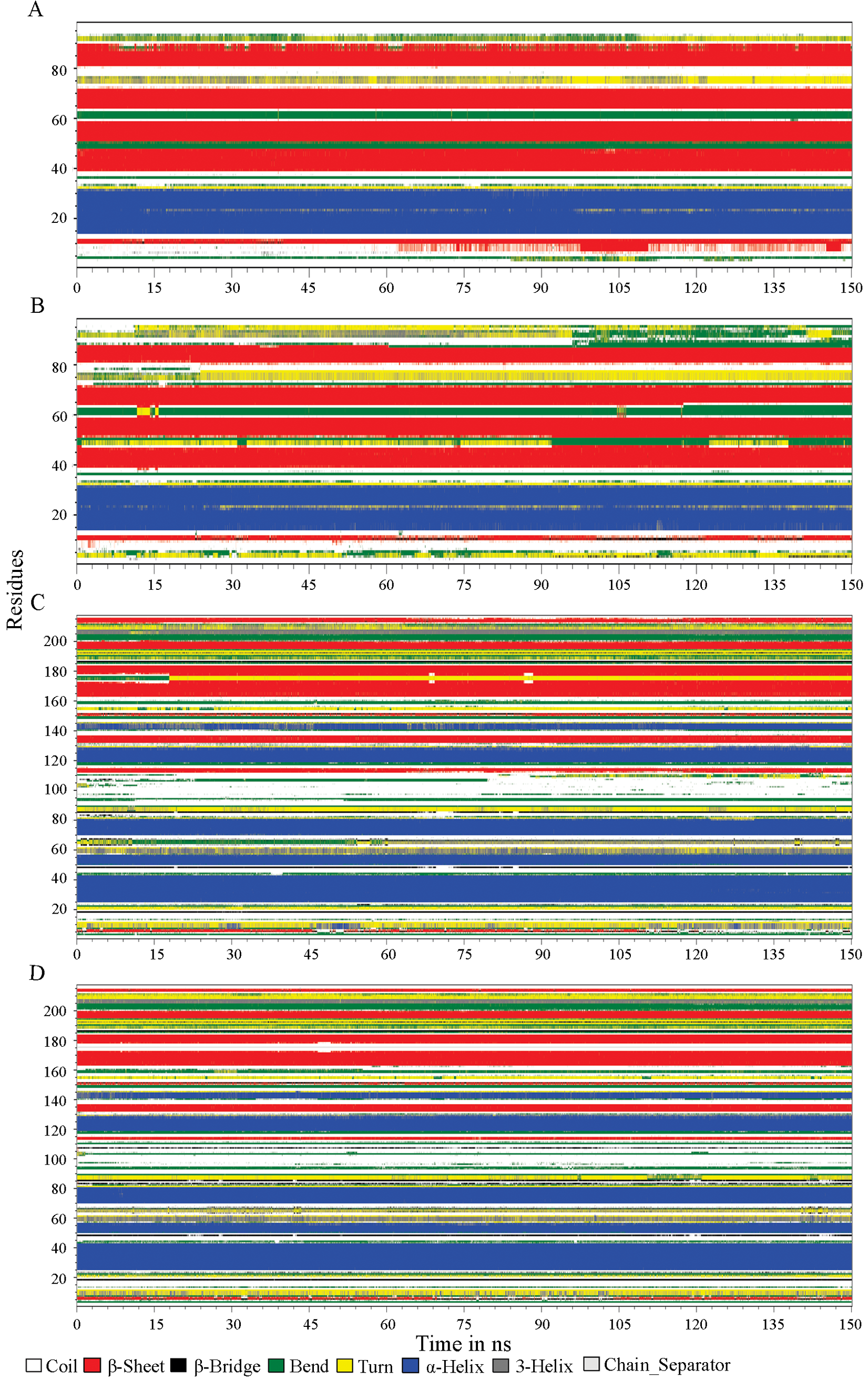

Supplement: S7 Fig — Secondary structure content of Stefin B in bound (A) and unbound (B) state and that of Cathepsin L1 in bound (C) and unbound (D) form. (TIF) [file pone.0164970.s007.tif]

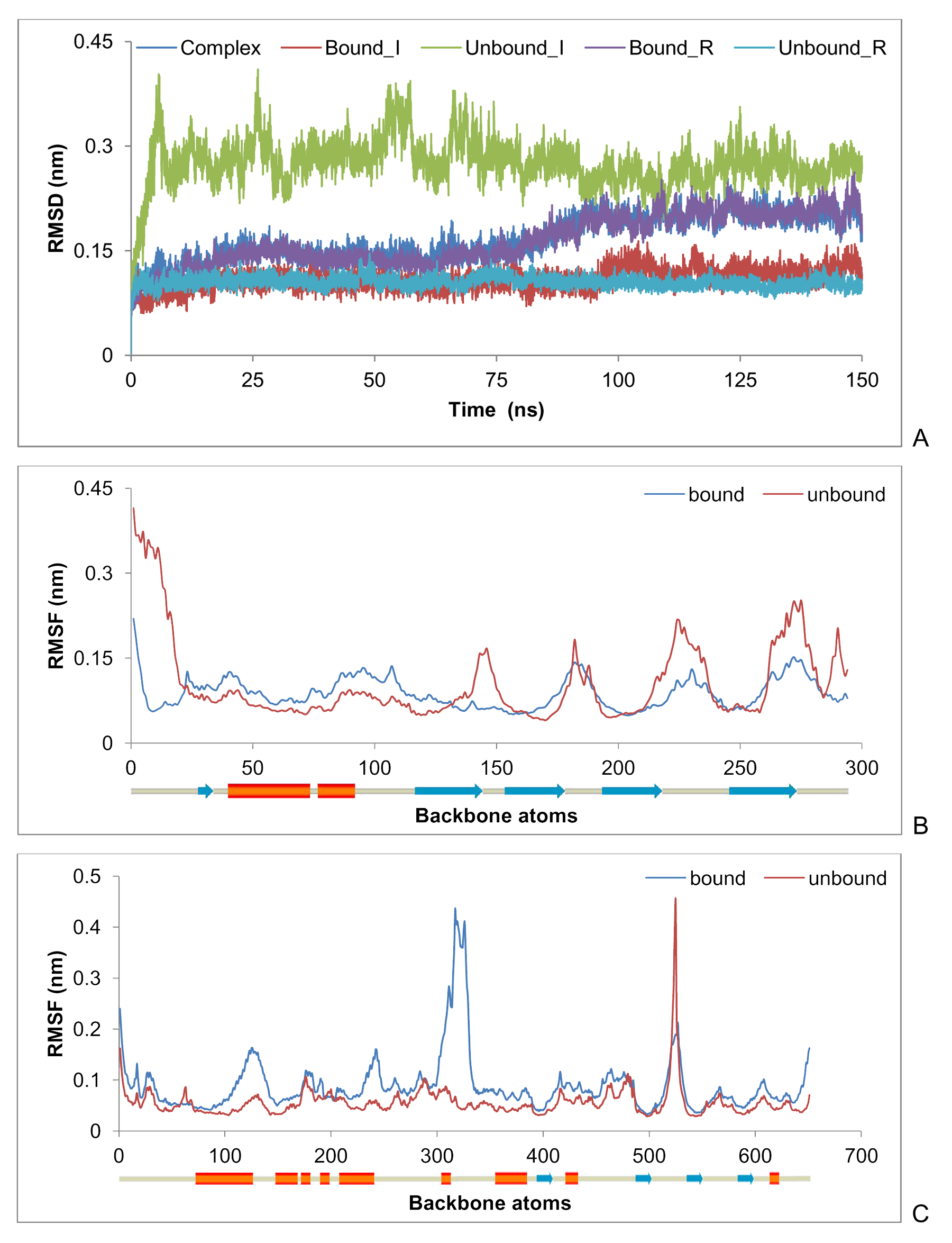

Supplement: S8 Fig — (A) Average backbone RMSD of the complex, inhibitor (I) and receptor (R) in bound and unbound state. RMSF of Stefin B (B) and Cathepsin L1 (C) in complexed form and in free state in solution. (TIF) [file pone.0164970.s008.tif]

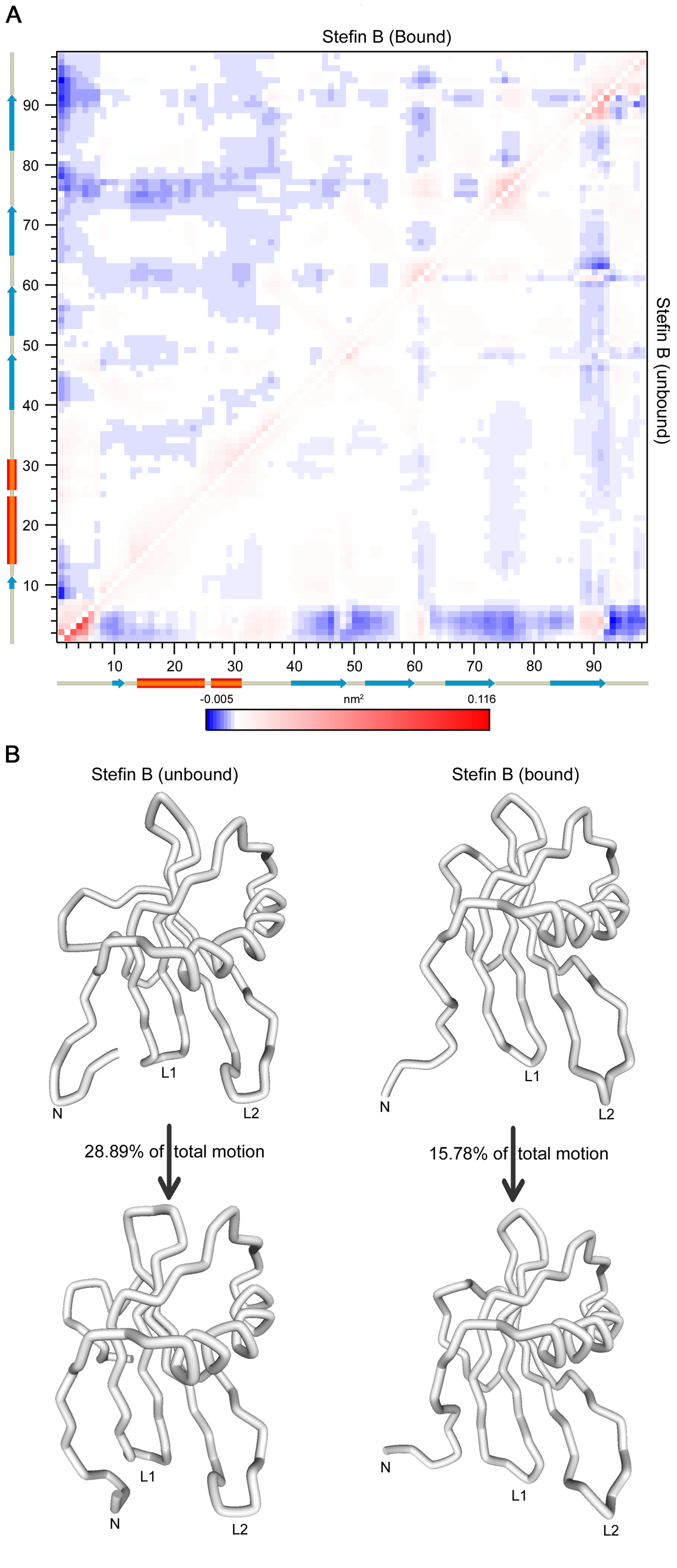

Supplement: S9 Fig — (A) Covariance matrix illustrating correlated and anticorrelated motions of bound (top left) and unbound (bottom right) stefin B. The secondary structure of stefin B backbone is represented along the axes (from left to right and from bottom to top). (b) Motion of the largest eigenvector of stefin B in absence (left) and presence (right) of cathepsin L1. (TIF) [file pone.0164970.s009.tif]

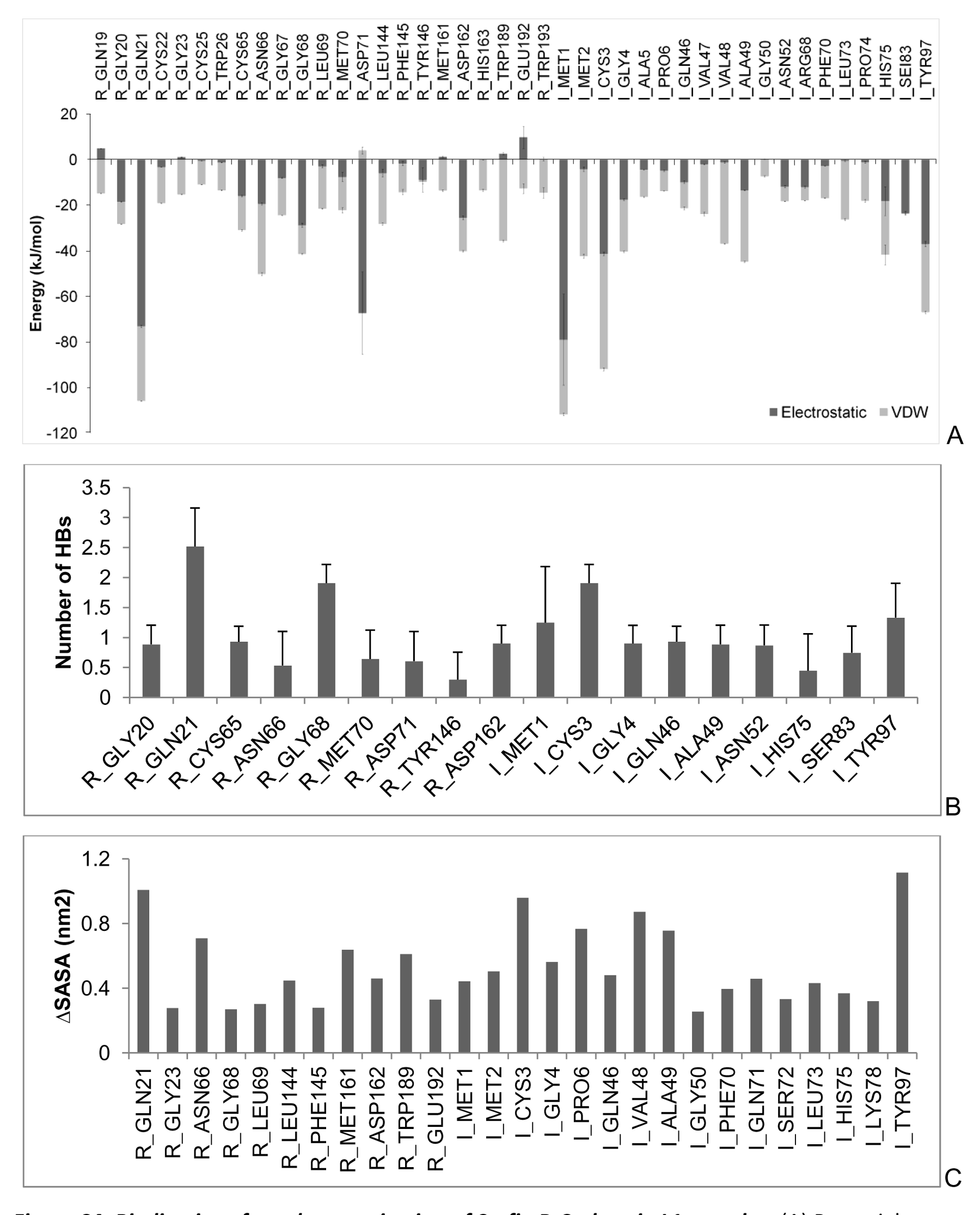

Supplement: S10 Fig — (A) Potential energy of interaction between binding interface residues of stefin B (I) & cathepsin L1 (R). Error bars represent the estimated error in GROMACS calculation. (B) Average number of HBs formed among interface residues. Error bars designate standard deviation. (C) Appreciable changes in SASA on complex formation among binding interface residues. (TIF) [file pone.0164970.s010.tif]

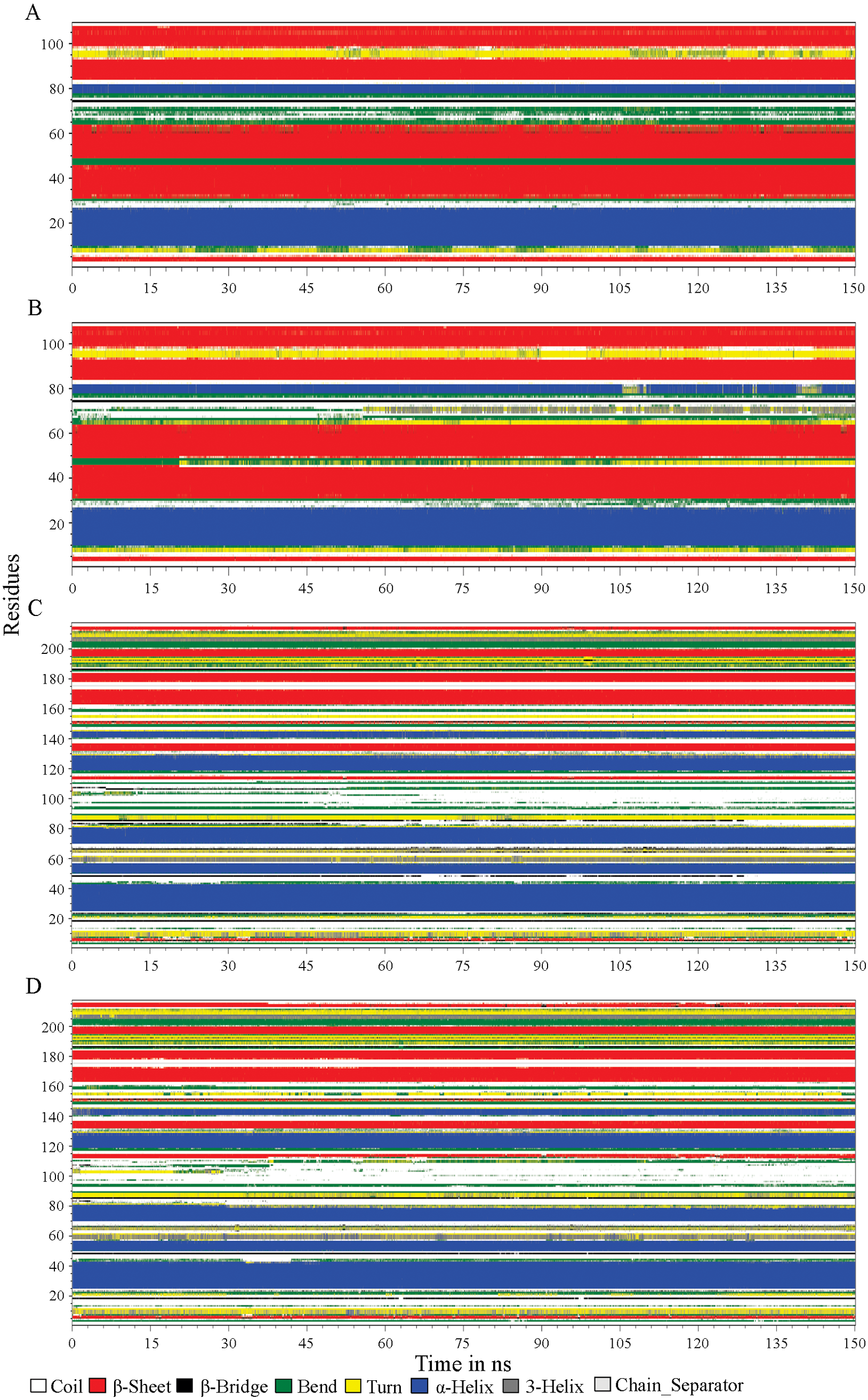

Supplement: S11 Fig — Secondary structure content of cystatin C in bound (A) and unbound (B) state and that of cathepsin L1 in bound (C) and unbound (D) form. (TIF) [file pone.0164970.s011.tif]

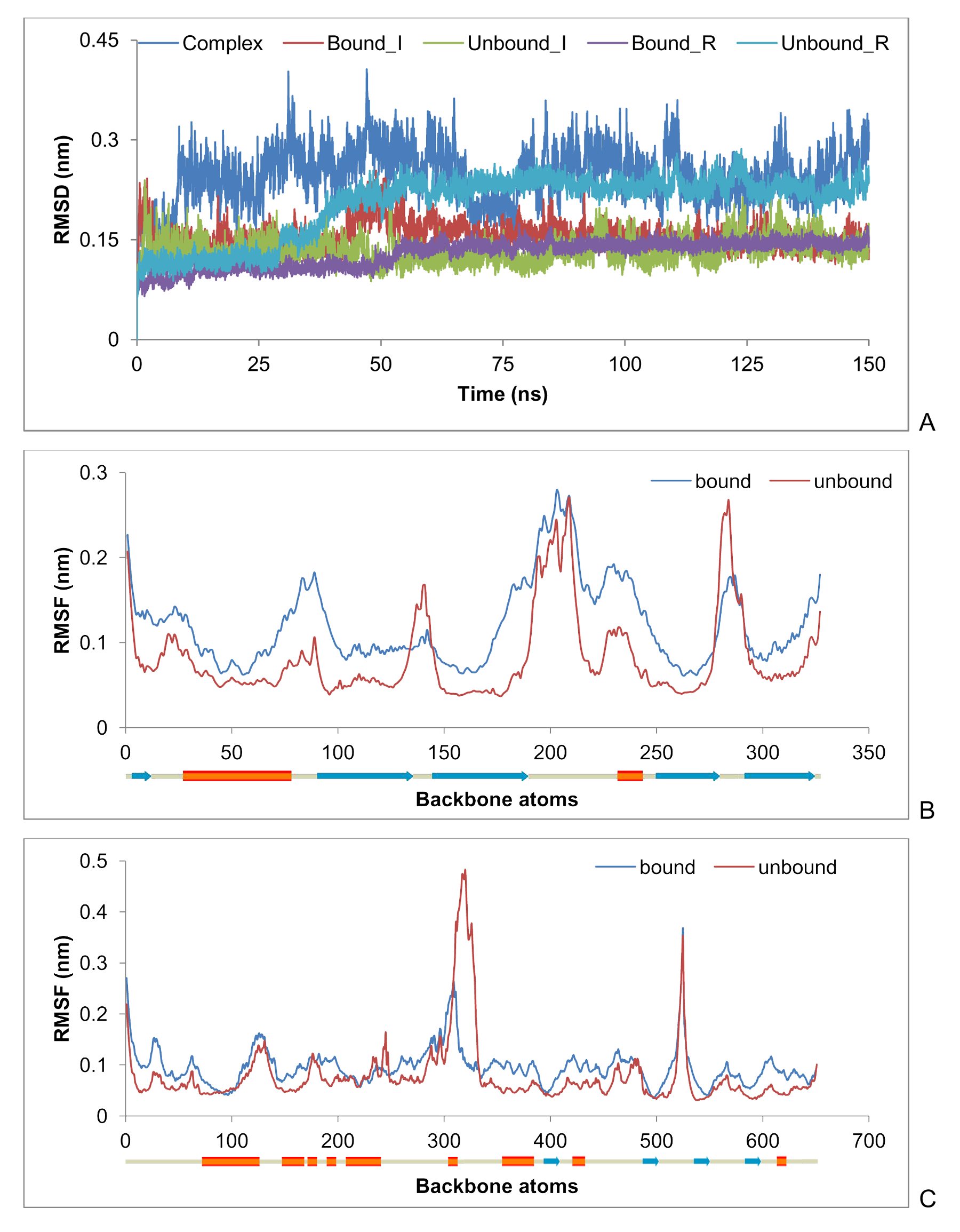

Supplement: S12 Fig — (A) Average backbone RMSD of the complex, inhibitor (I) and receptor (R) in bound and unbound state. RMSF of cystatin C (B) and cathepsin L1 (C) in complexed form and in free state in solution. (TIF) [file pone.0164970.s012.tif]

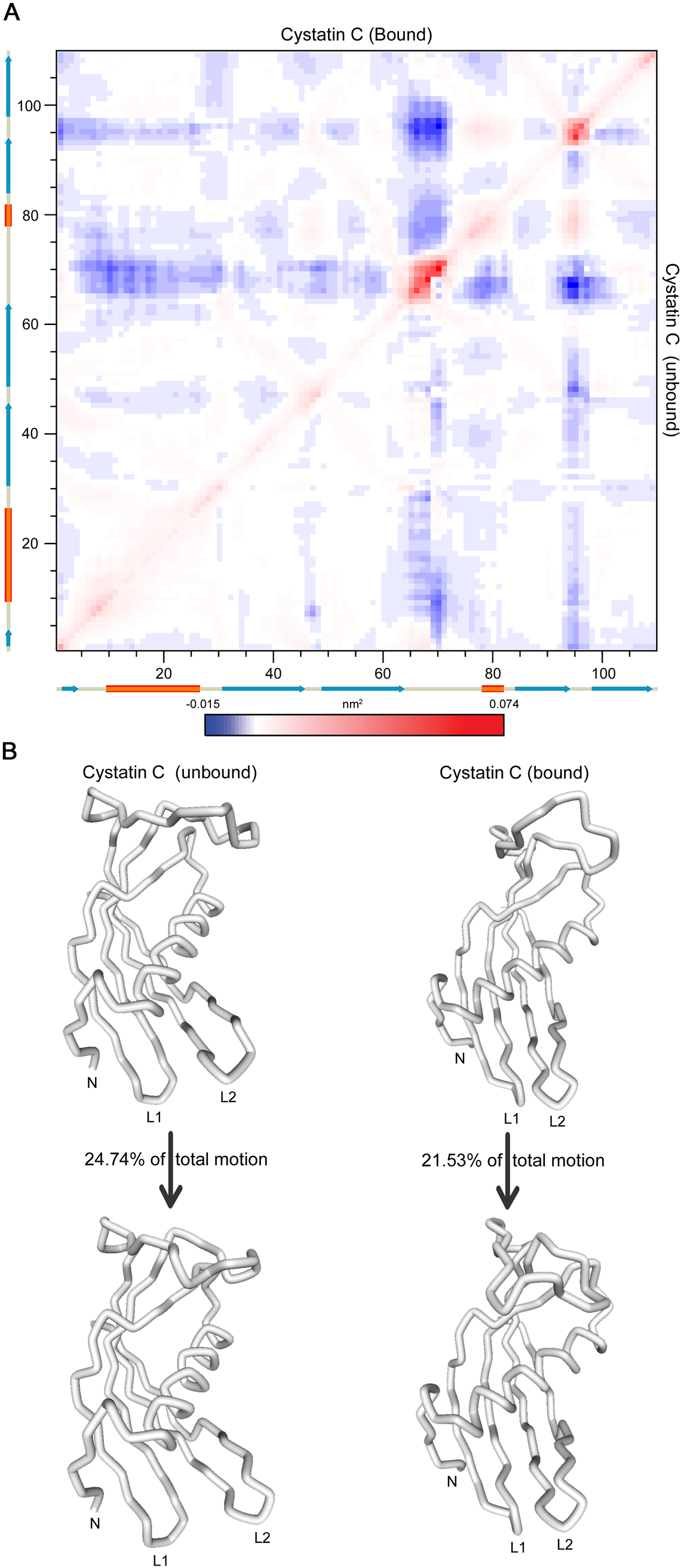

Supplement: S13 Fig — (A) Covariance matrix illustrating correlated and anticorrelated motions of bound (top left) and unbound (bottom right) cystatin C. The secondary structure of cystatin C backbone is represented along the axes (from left to right and from bottom to top). (b) Motion of the largest eigenvector of cystatin C in absence (left) and presence (right) of CL1. (TIF) [file pone.0164970.s013.tif]

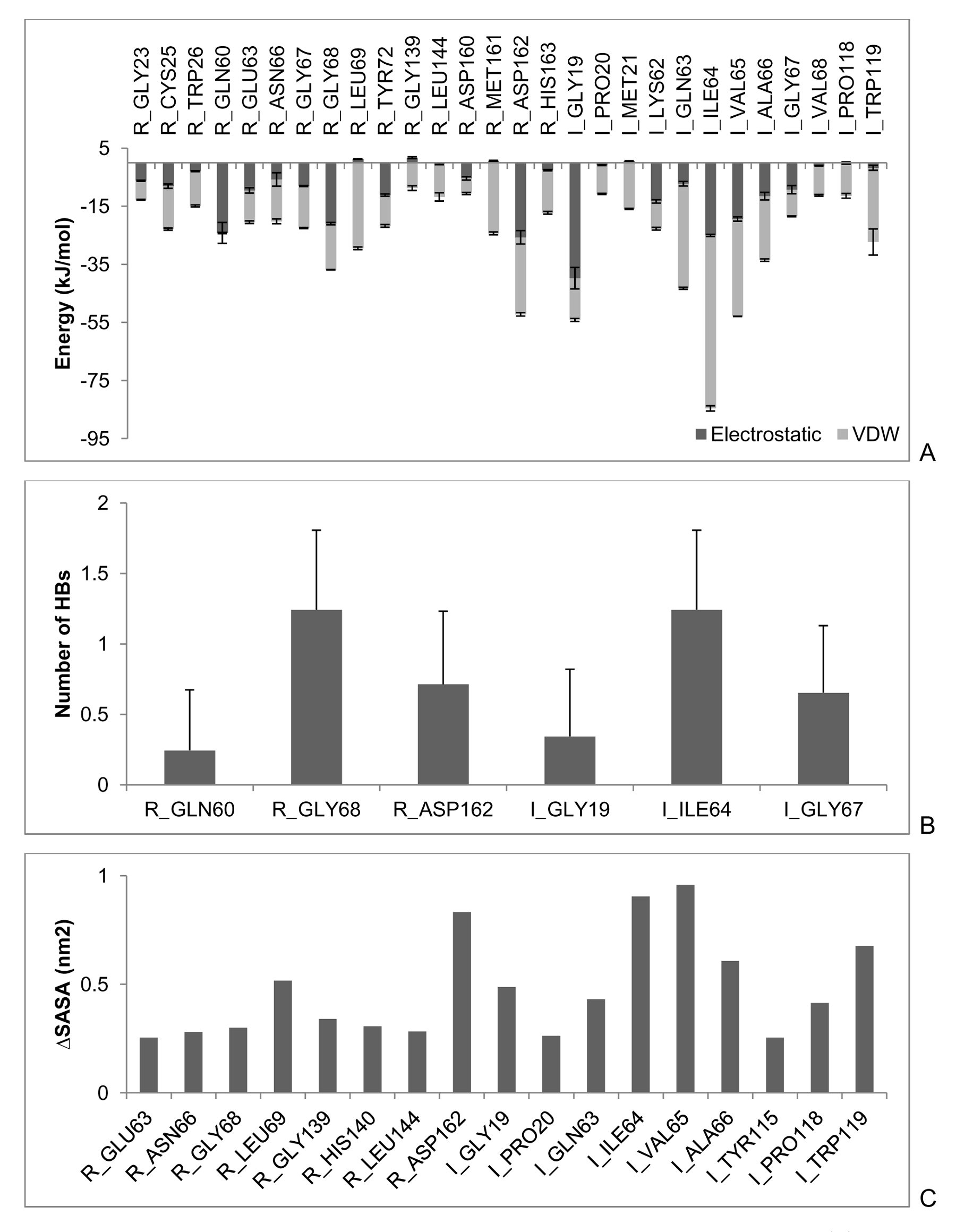

Supplement: S14 Fig — (A) Potential energy of interaction between binding interface residues of cystatin C (I) & cathepsin L1 (R). Error bars represent the estimated error in GROMACS calculation. (B) Average number of HBs formed among interface residues. Error bars designate standard deviation. (C) Appreciable changes in SASA on complex formation among binding interface residues. (TIF) [file pone.0164970.s014.tif]

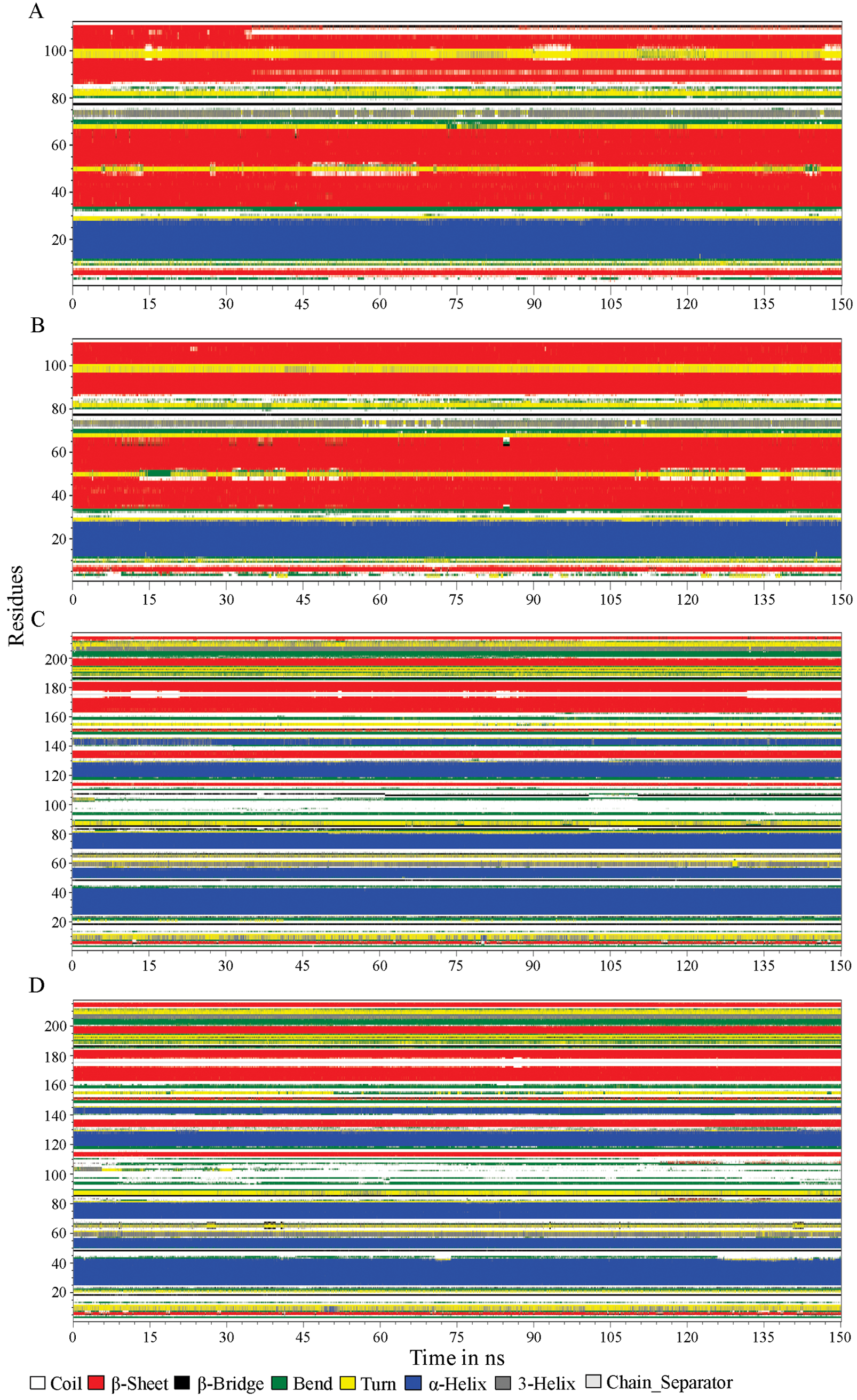

Supplement: S15 Fig — Secondary structure content of cystatin D in bound (A) and unbound (B) state & that of cathepsin L1 in bound (C) and unbound (D) form. (TIF) [file pone.0164970.s015.tif]

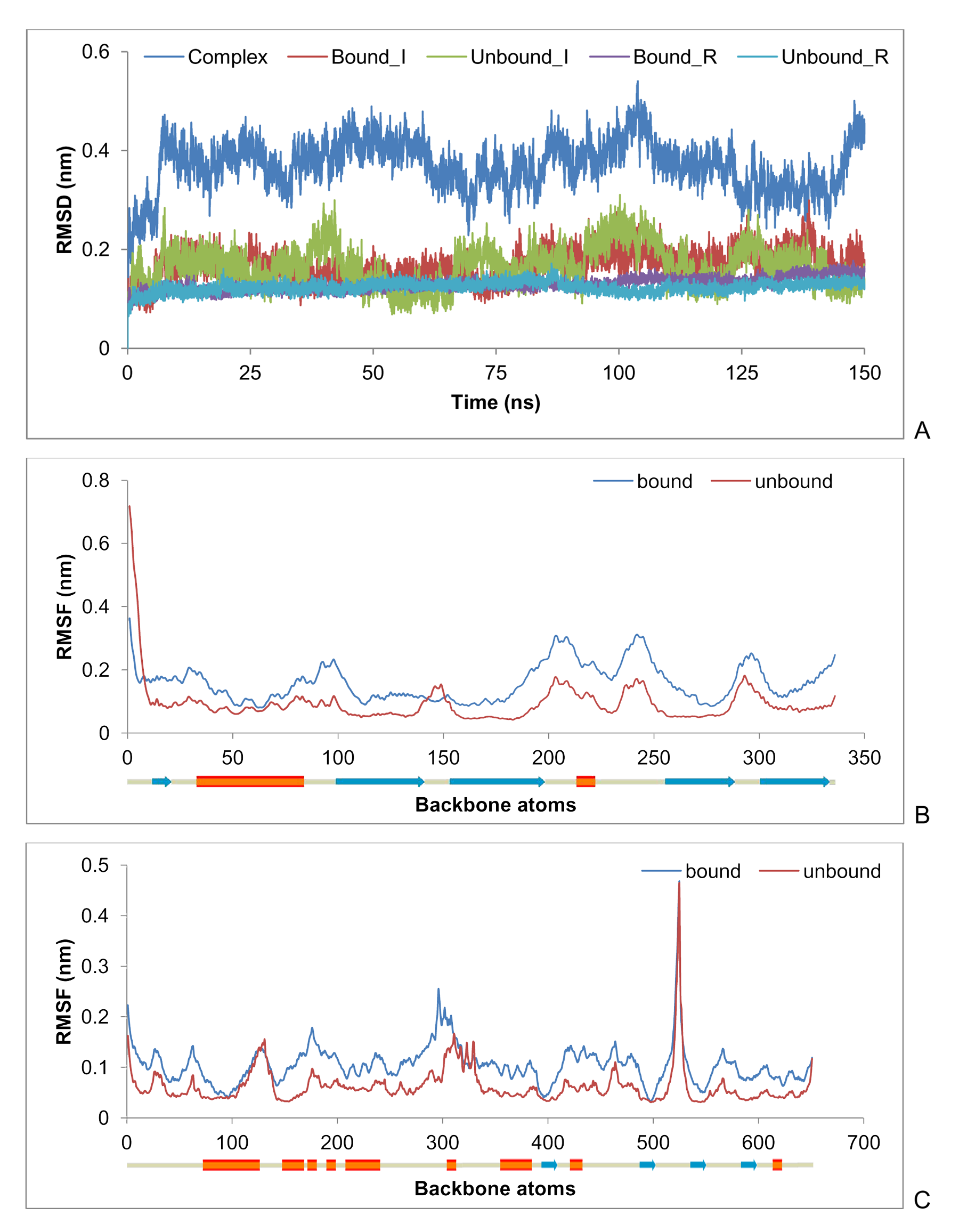

Supplement: S16 Fig — (A) Average backbone RMSD of the complex, inhibitor (I) and receptor (R) in bound and unbound state. RMSF of cystatin D (B) and cathepsin L1 (C) in complexed form and in free state in solution. (TIF) [file pone.0164970.s016.tif]

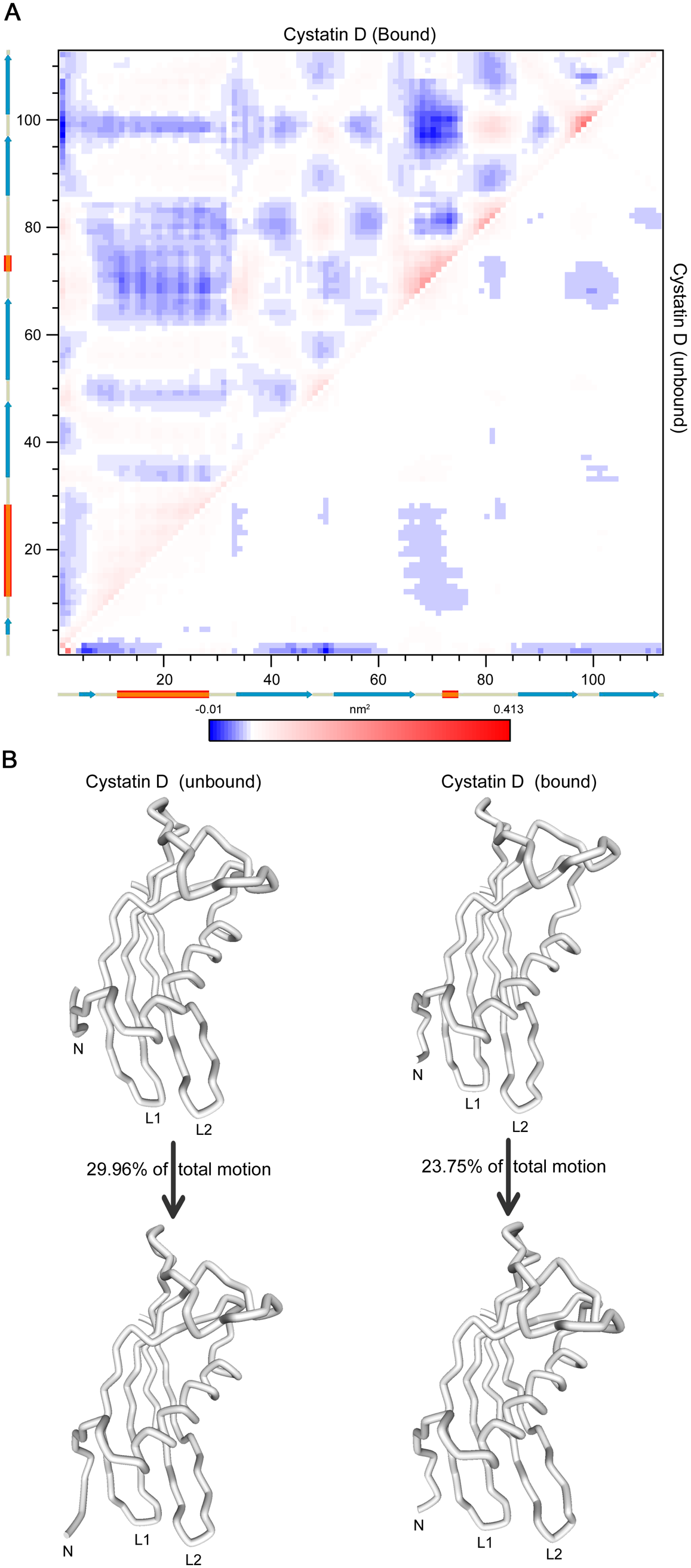

Supplement: S17 Fig — (A) Covariance matrix illustrating correlated and anticorrelated motions of bound (top left) and unbound (bottom right) cystatin D. The secondary structure of cystatin D backbone is represented along the axes (from left to right and from bottom to top). (b) Motion of the largest eigenvector of cystatin D in absence (left) and presence (right) of cathepsin L1. (TIF) [file pone.0164970.s017.tif]

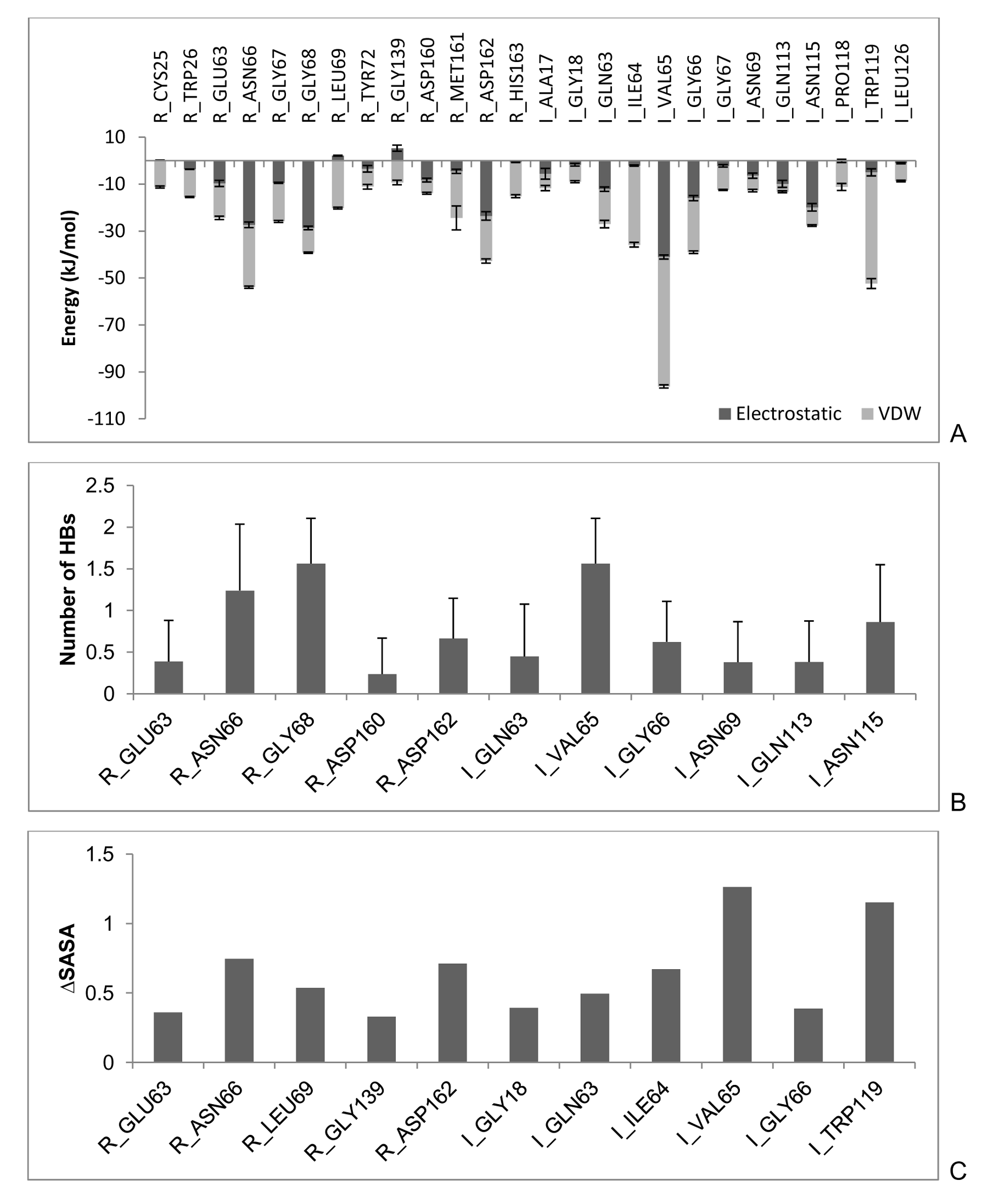

Supplement: S18 Fig — (A) Potential energy of interaction between binding interface residues of cystatin D (I) & cathepsin L1 (R). Error bars represent the estimated error in GROMACS calculation. (B) Average number of HBs formed among interface residues. Error bars designate standard deviation. (C) Appreciable changes in SASA on complex formation among binding interface residues. (TIF) [file pone.0164970.s018.tif]

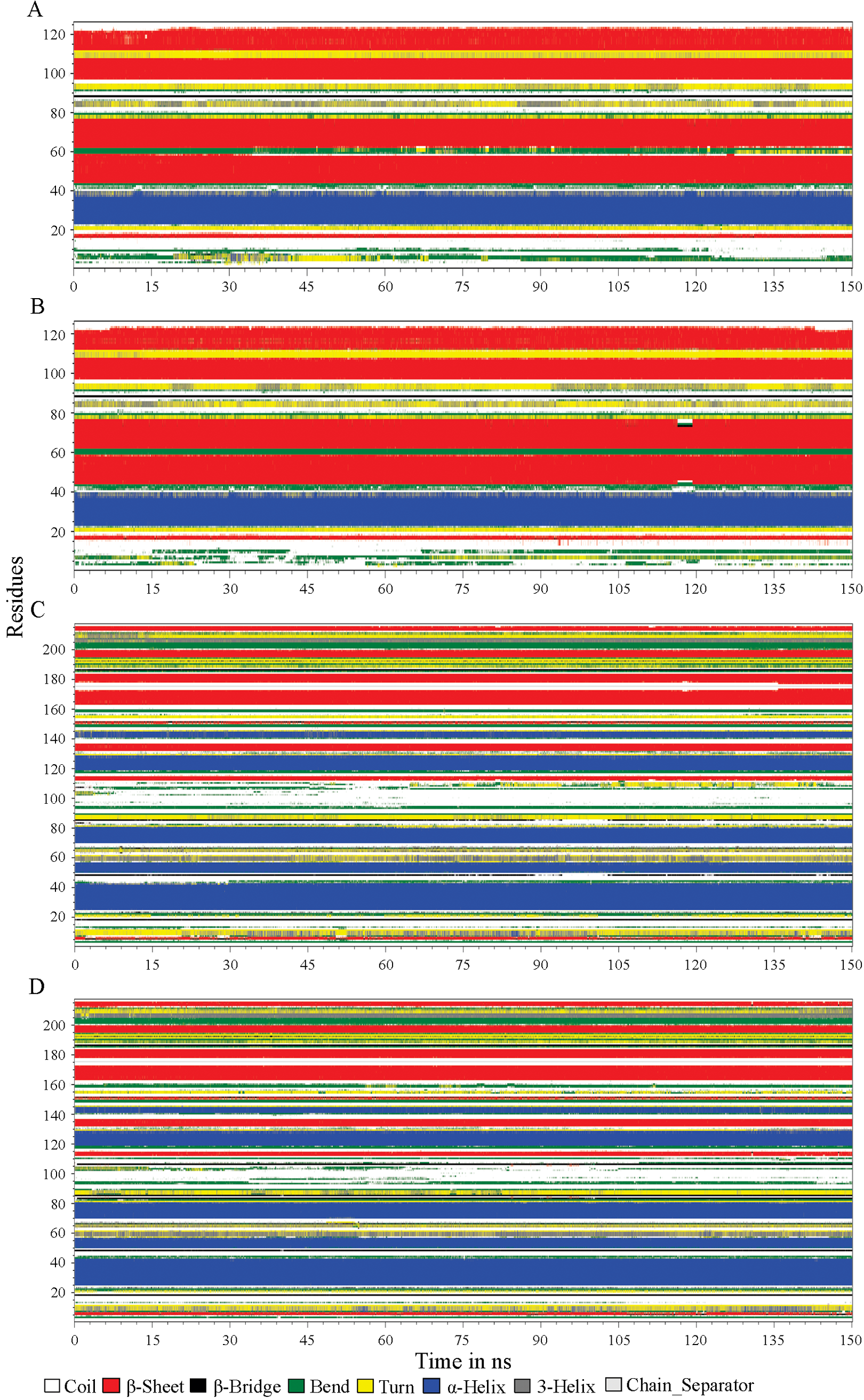

Supplement: S19 Fig — Secondary structure content of cystatin F in bound (A) and unbound (B) state & that of cathepsin L1 in bound (C) and unbound (D) form. (TIF) [file pone.0164970.s019.tif]

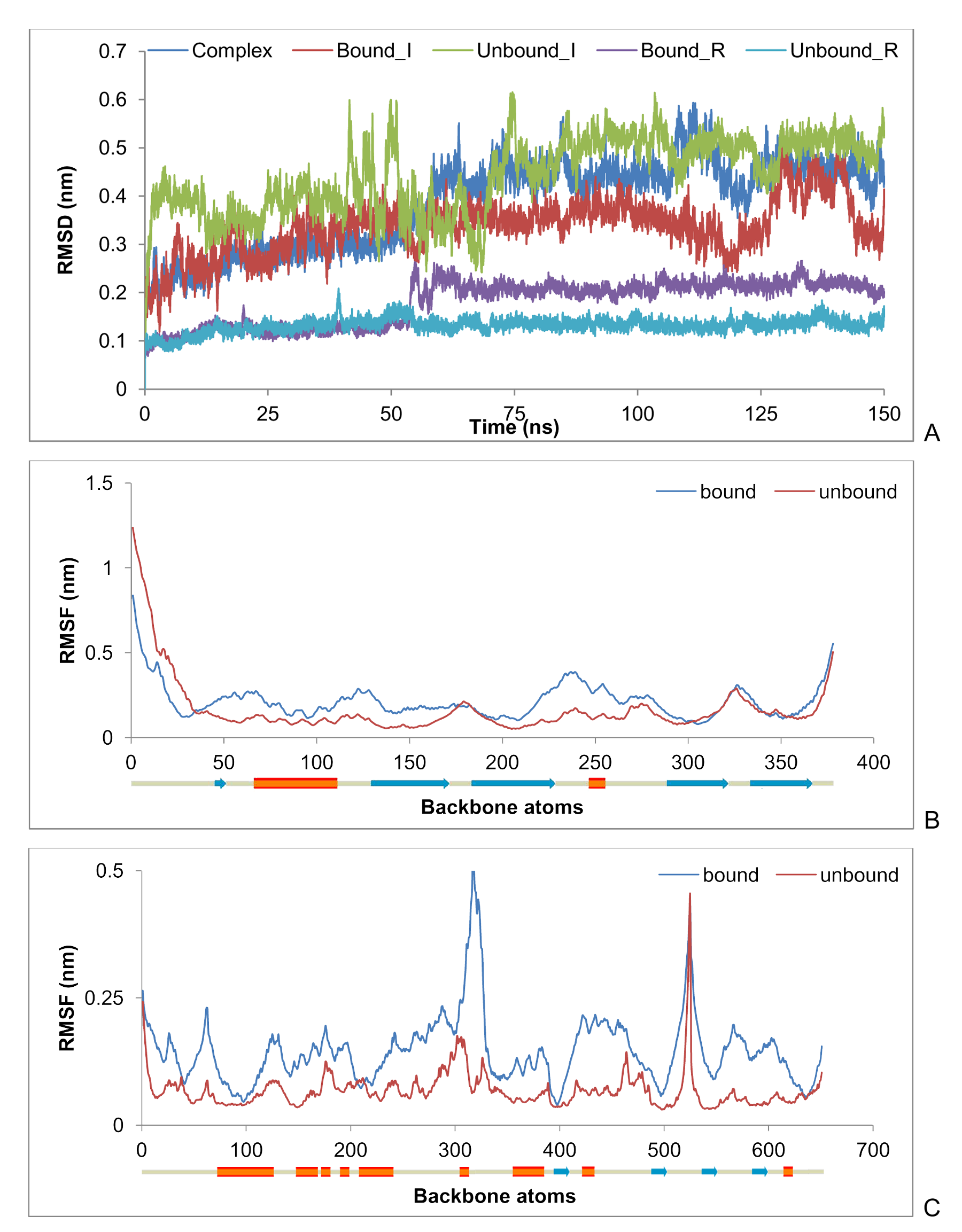

Supplement: S20 Fig — (A) Average backbone RMSD of the complex, inhibitor (I) and receptor (R) in bound and unbound state. RMSF of cystatin F (B) and cathepsin L1 (C) in complexed form and in free state in solution. (TIF) [file pone.0164970.s020.tif]

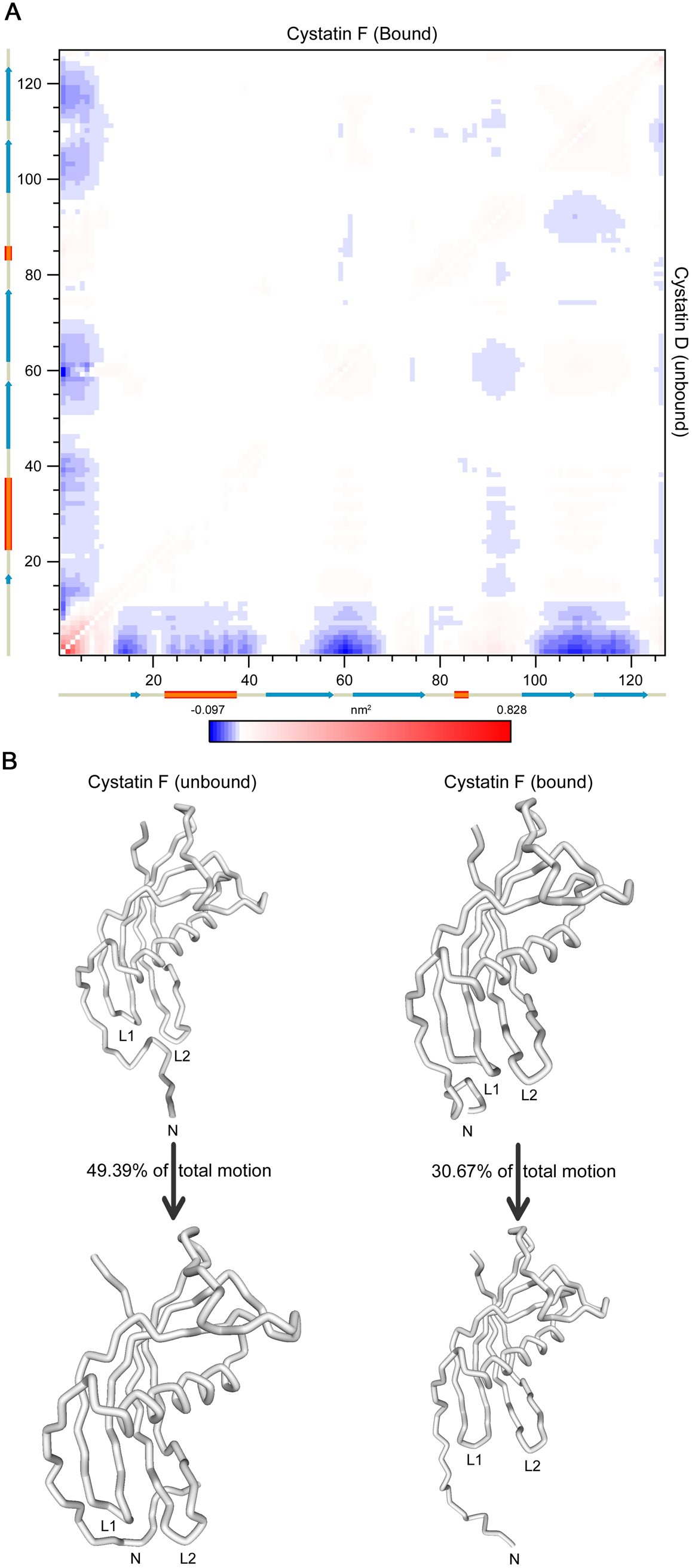

Supplement: S21 Fig — (A) Covariance matrix illustrating correlated and anticorrelated motions of bound (top left) and unbound (bottom right) cystatin F. The secondary structure of cystatin F backbone is represented along the axes (from left to right and from bottom to top). (b) Motion of the largest eigenvector of cystatin F in absence (left) and presence (right) of cathepsin L1. (TIF) [file pone.0164970.s021.tif]

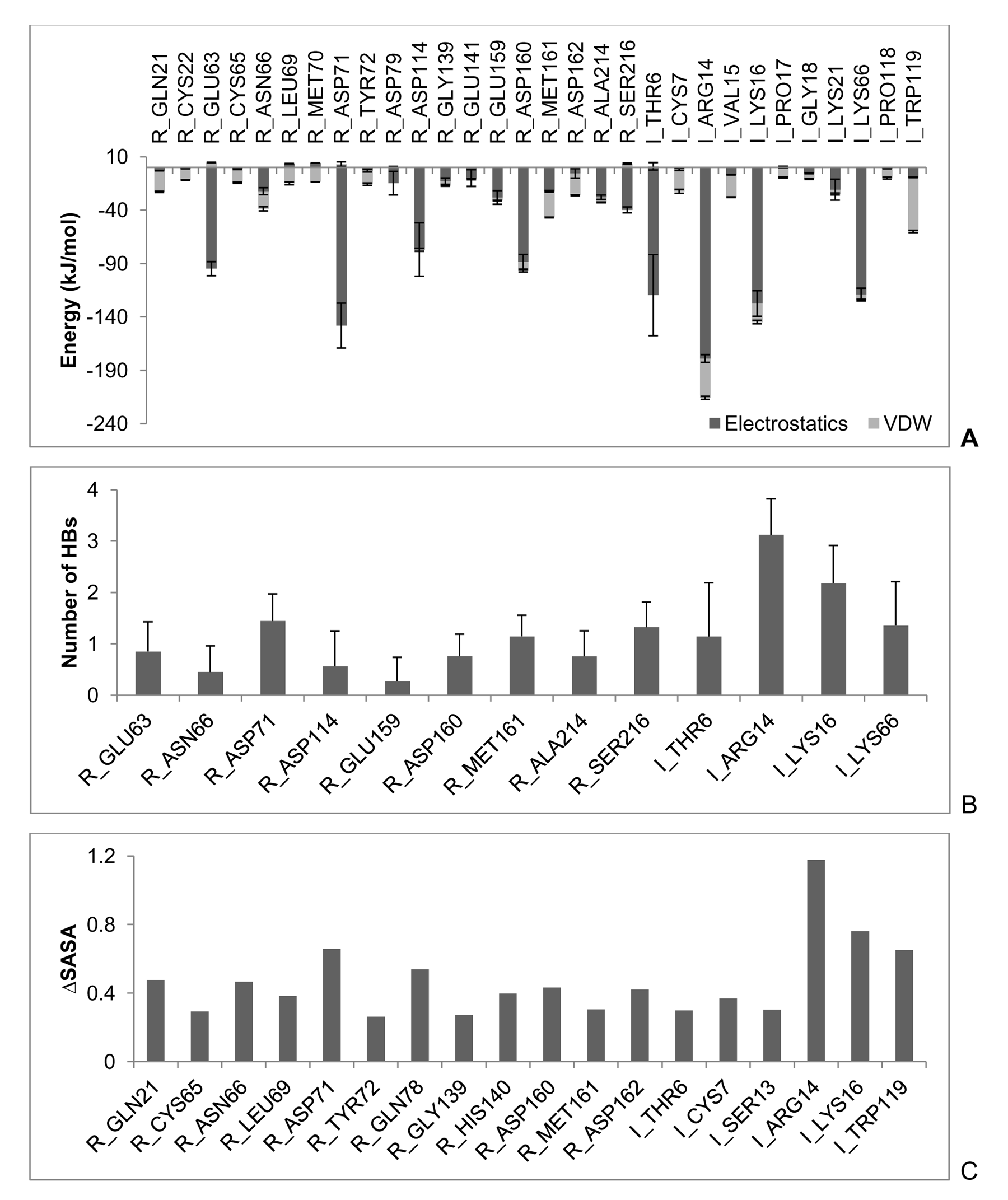

Supplement: S22 Fig — (A) Potential energy of interaction between binding interface residues of cystatin F (I) & cathepsin L1 (R). Error bars represent the estimated error in GROMACS calculation. (B) Average number of HBs formed among interface residues. Error bars designate standard deviation. (C) Appreciable changes in SASA on complex formation among binding interface residues. (TIF) [file pone.0164970.s022.tif]

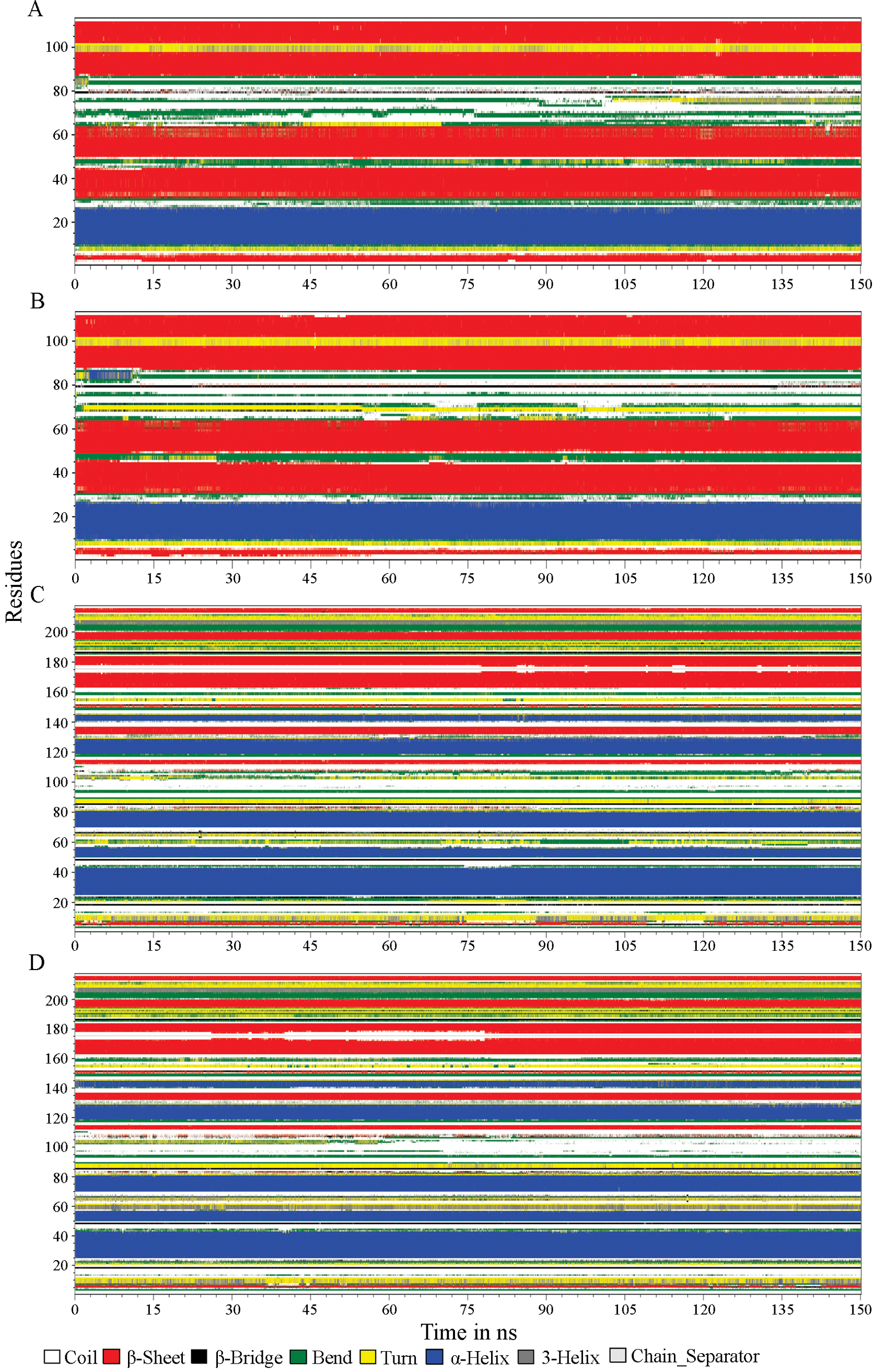

Supplement: S23 Fig — Secondary structure content of cystatin M/E in bound (A) and unbound (B) state and that of cathepsin L1 in bound (C) and unbound (D) form. (TIF) [file pone.0164970.s023.tif]

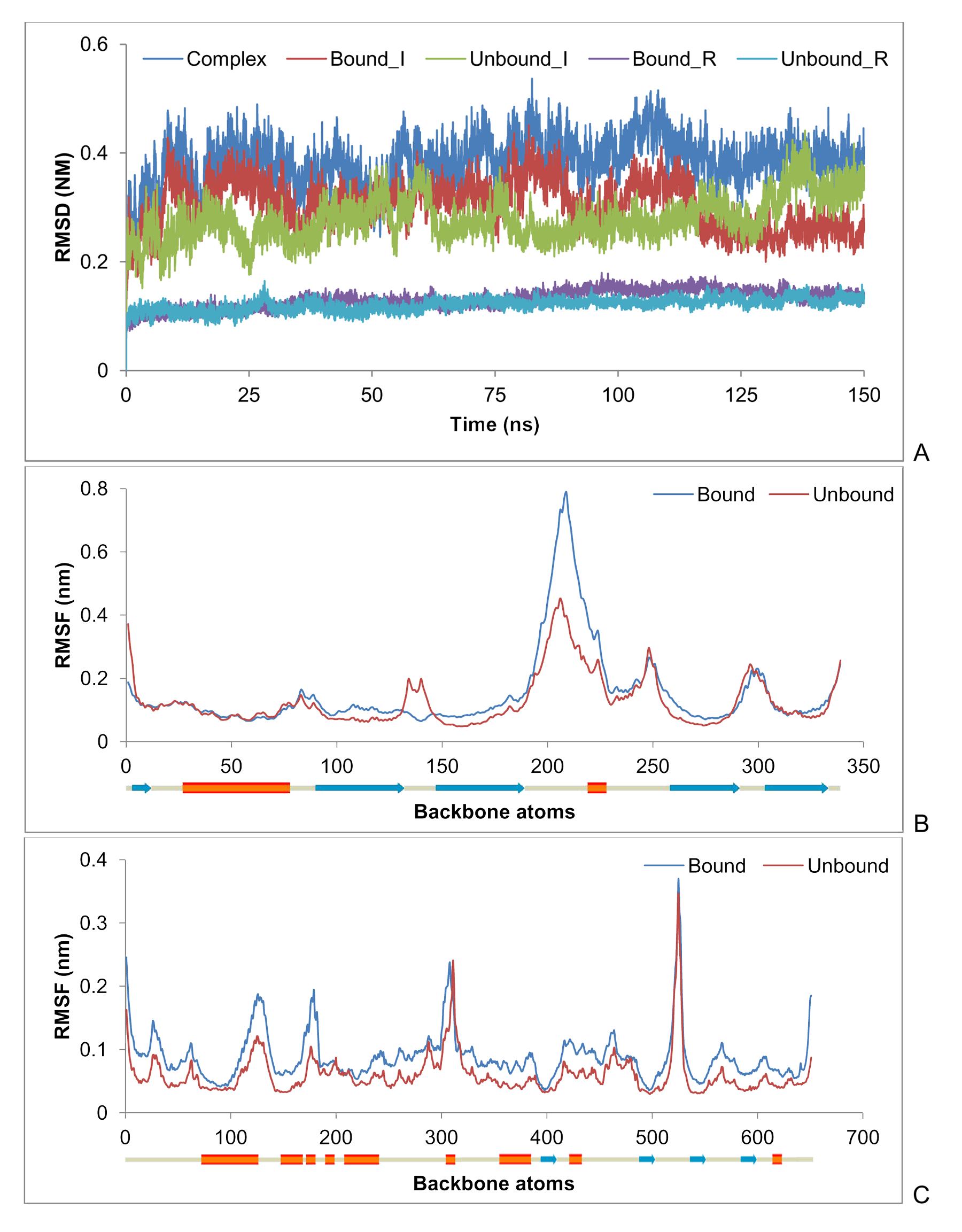

Supplement: S24 Fig — (A) Average backbone RMSD of the complex, inhibitor (I) and receptor (R) in bound and unbound state. RMSF of cystatin M/E (B) and cathepsin L1 (C) in complexed form and in free state in solution. (TIF) [file pone.0164970.s024.tif]

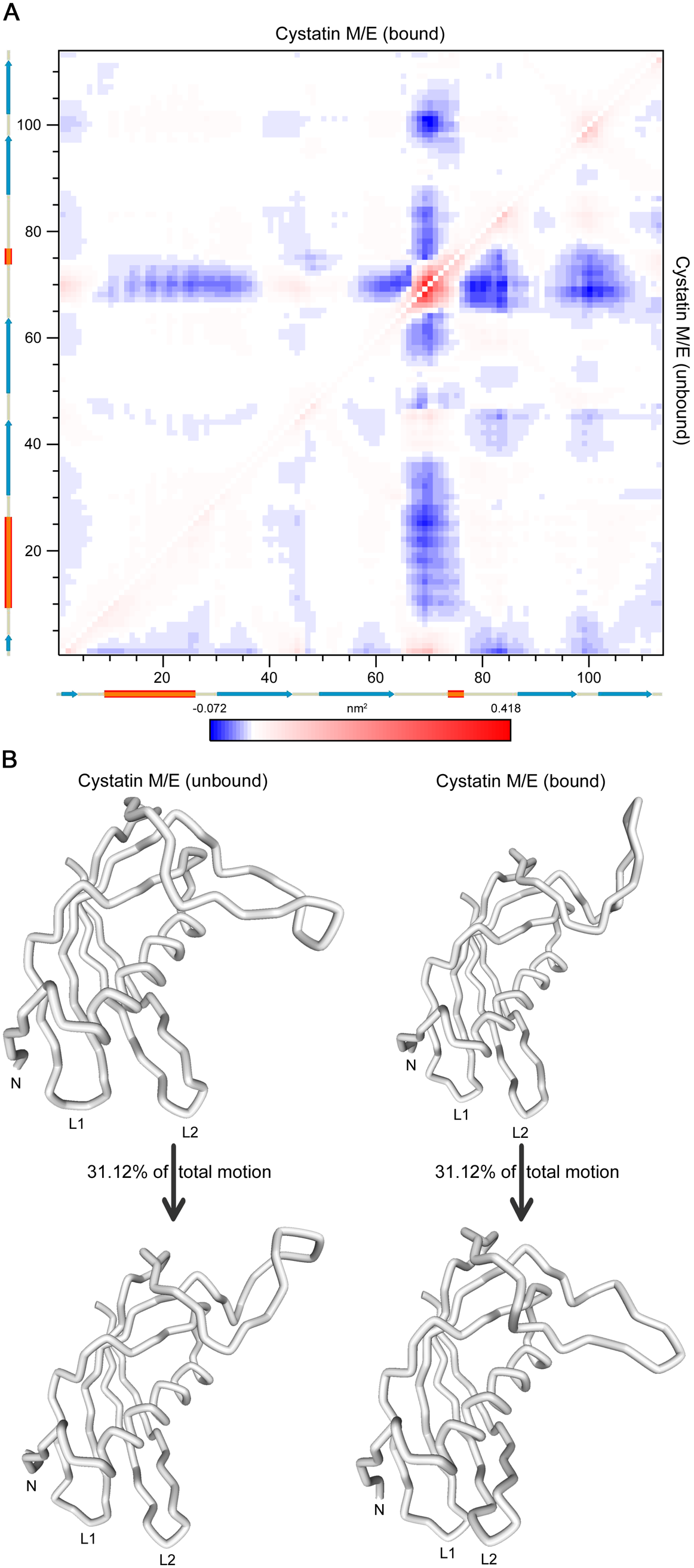

Supplement: S25 Fig — (A) Covariance matrix illustrating correlated and anticorrelated motions of bound (top left) and unbound (bottom right) cystatin M/E. The secondary structure of cystatin M/E backbone is represented along the axes (from left to right and from bottom to top). (b) Motion of the largest eigenvector of cystatin M/E in absence (left) and presence (right) of cathepsin L1. (TIF) [file pone.0164970.s025.tif]

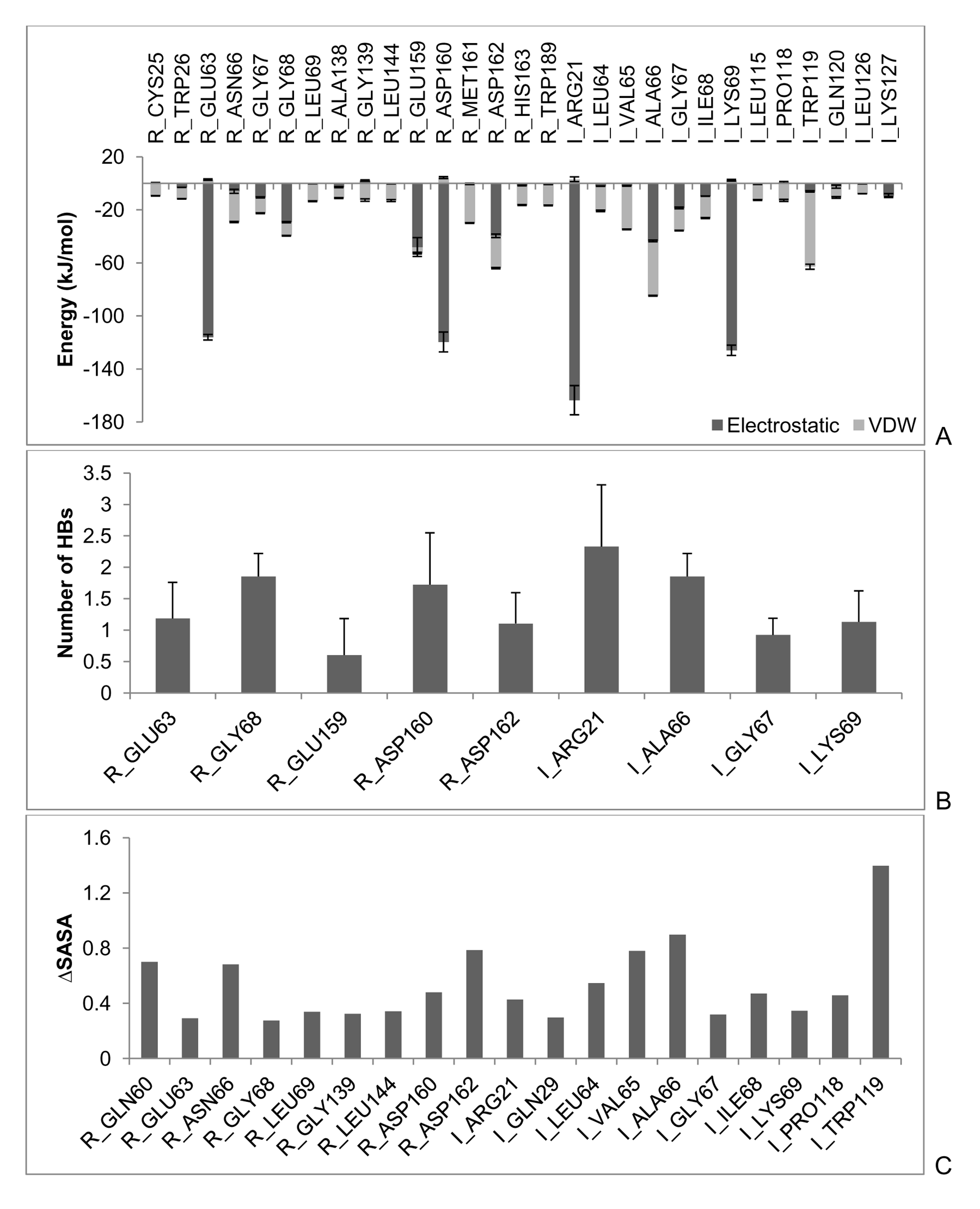

Supplement: S26 Fig — (A) Potential energy of interaction between binding interface residues of cystatin M/E (I) & cathepsin L1 (R). Error bars represent the estimated error in GROMACS calculation. (B) Average number of HBs formed among interface residues. Error bars designate standard deviation. (C) Appreciable changes in SASA on complex formation among binding interface residues. (TIF) [file pone.0164970.s026.tif]

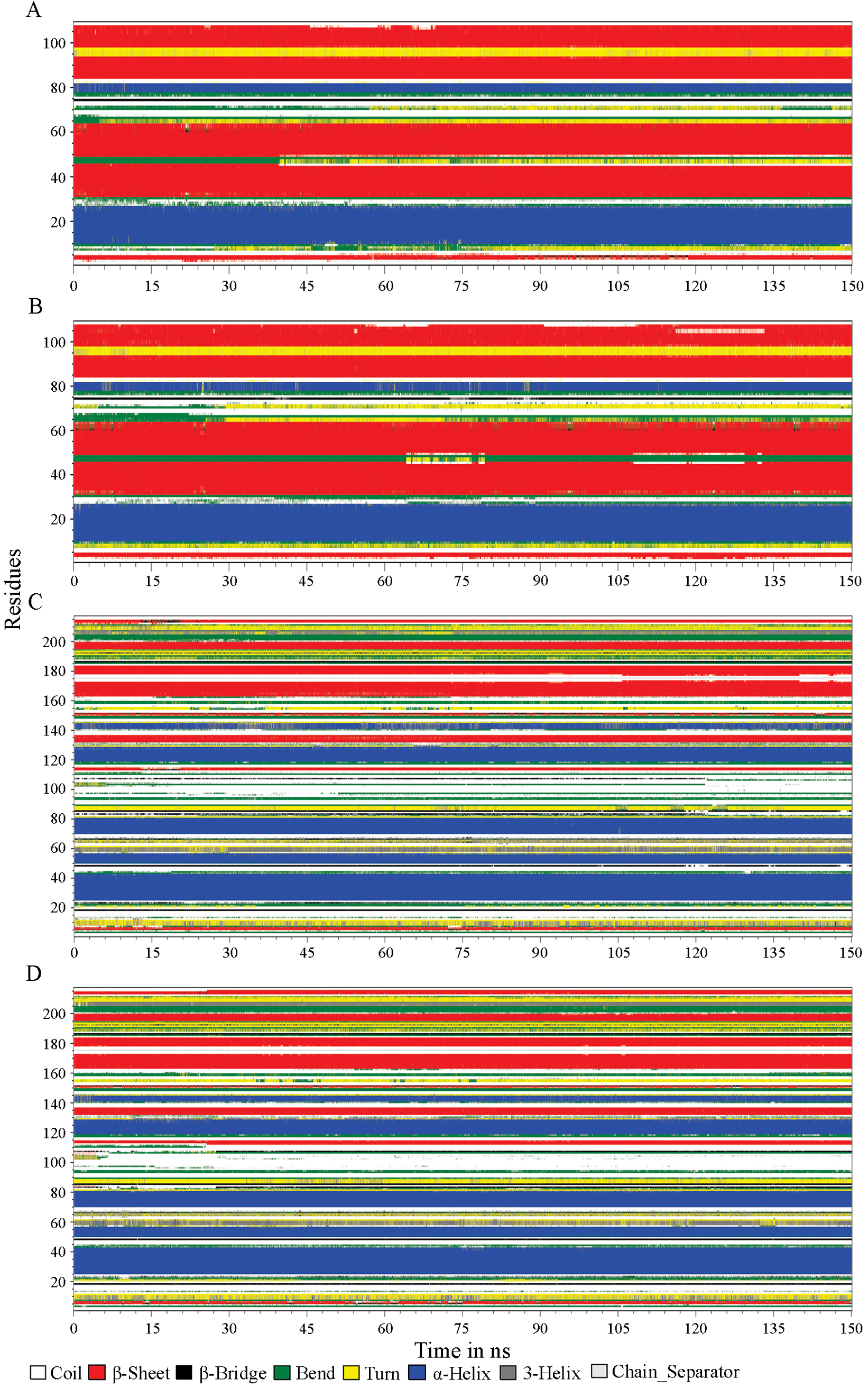

Supplement: S27 Fig — Secondary structure content of cystatin S in bound (A) and unbound (B) state & that of cathepsin L1 in bound (C) and unbound (D) form. (TIF) [file pone.0164970.s027.tif]

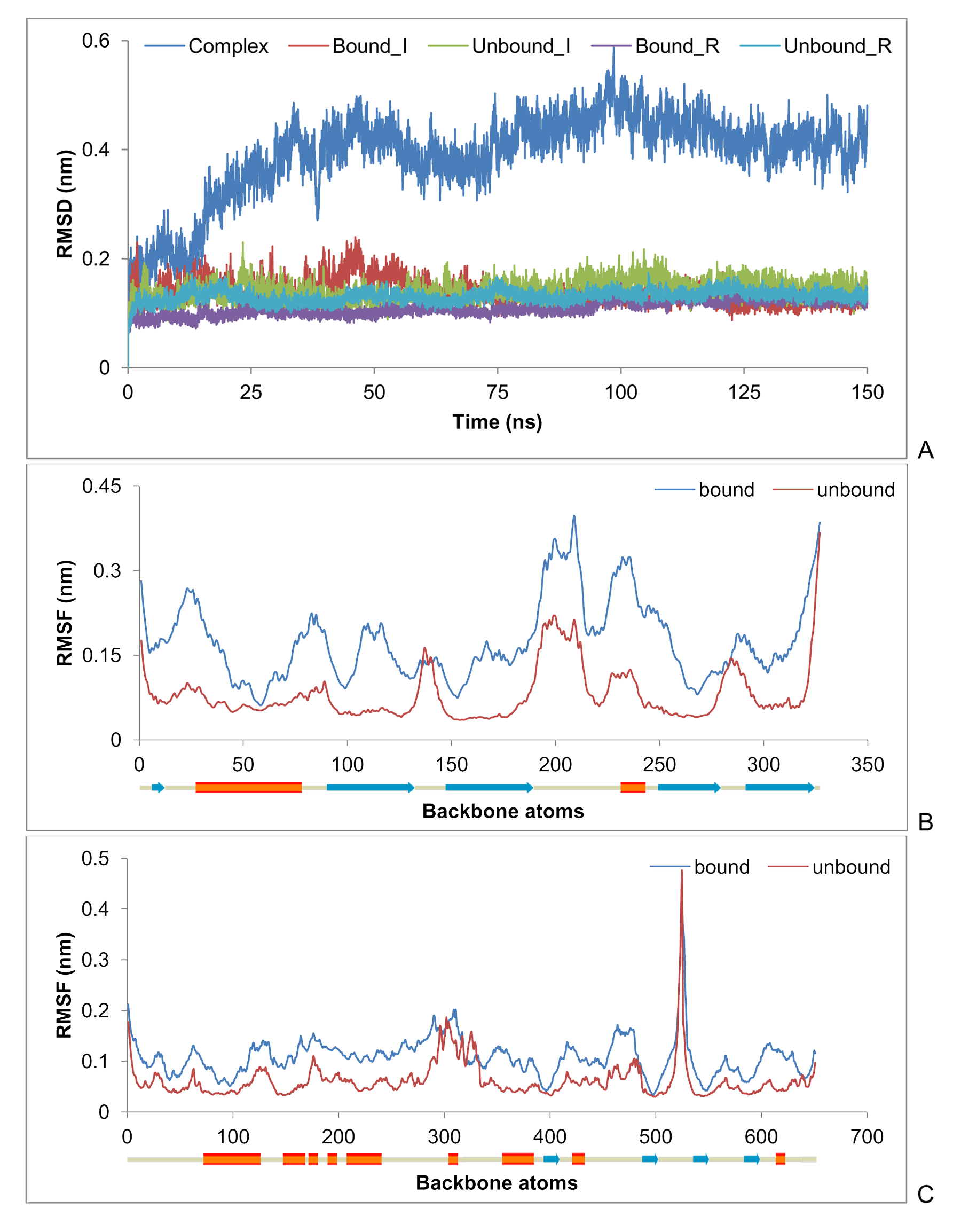

Supplement: S28 Fig — (A) Average backbone RMSD of the complex, inhibitor (I) and receptor (R) in bound and unbound state. RMSF of cystatin S (B) and cathepsin L1 (C) in complexed form and in free state in solution. (TIF) [file pone.0164970.s028.tif]

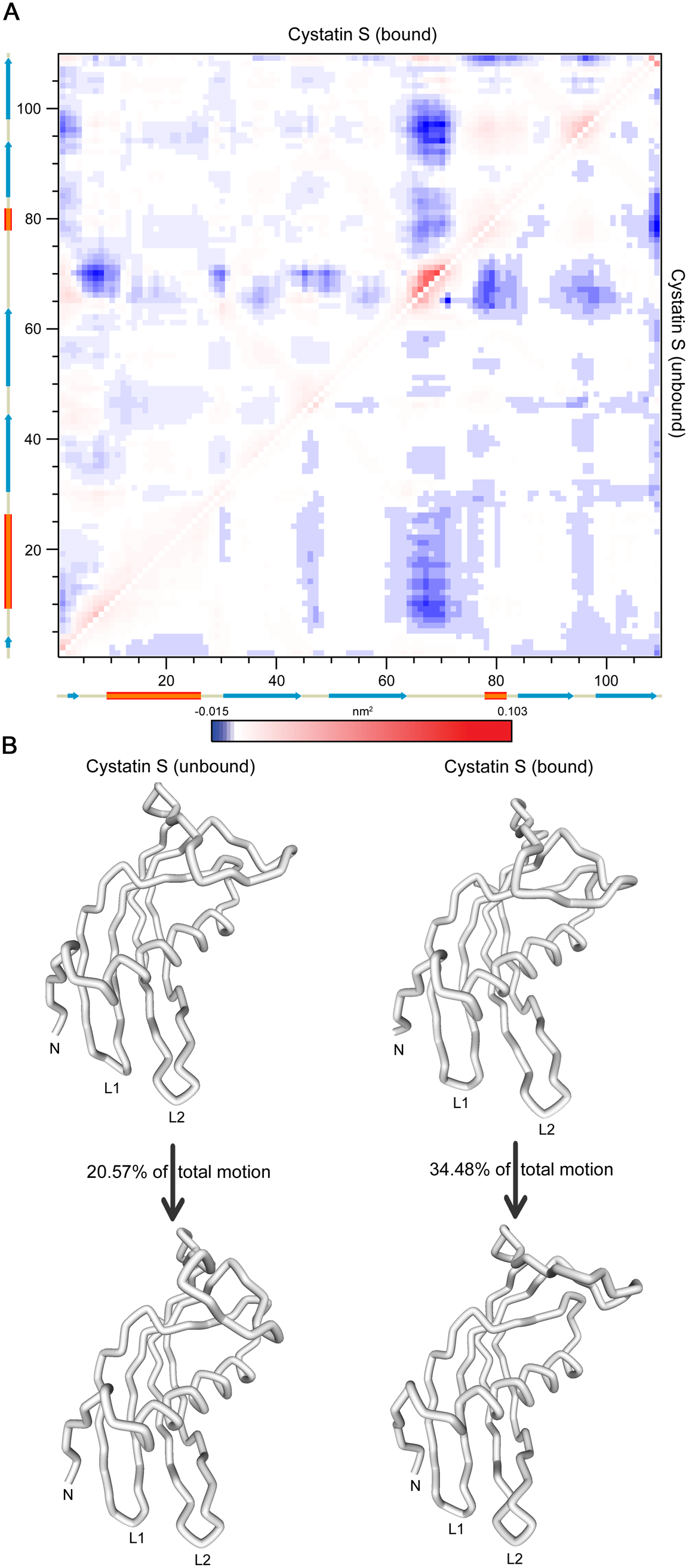

Supplement: S29 Fig — (A) Covariance matrix illustrating correlated and anticorrelated motions of bound (top left) and unbound (bottom right) cystatin S. The secondary structure of cystatin S backbone is represented along the axes (from left to right and from bottom to top). (b) Motion of the largest eigenvector of cystatin S in absence (left) and presence (right) of cathepsin L1. (TIF) [file pone.0164970.s029.tif]

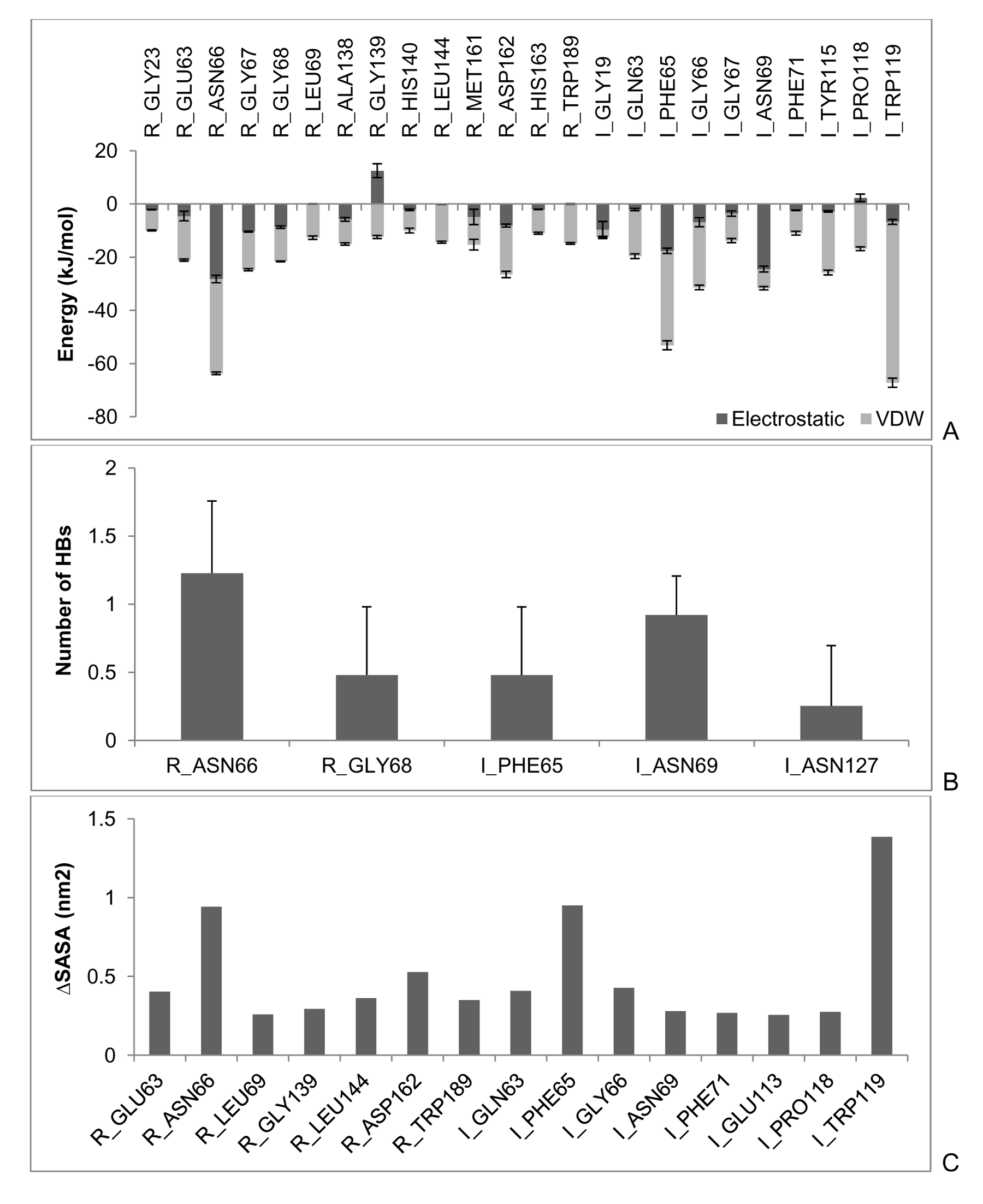

Supplement: S30 Fig — (A) Potential energy of interaction between binding interface residues of cystatin S (I) & cathepsin L1 (R). Error bars represent the estimated error in GROMACS calculation. (B) Average number of HBs formed among interface residues. Error bars designate standard deviation. (C) Appreciable changes in SASA on complex formation among binding interface residues. (TIF) [file pone.0164970.s030.tif]

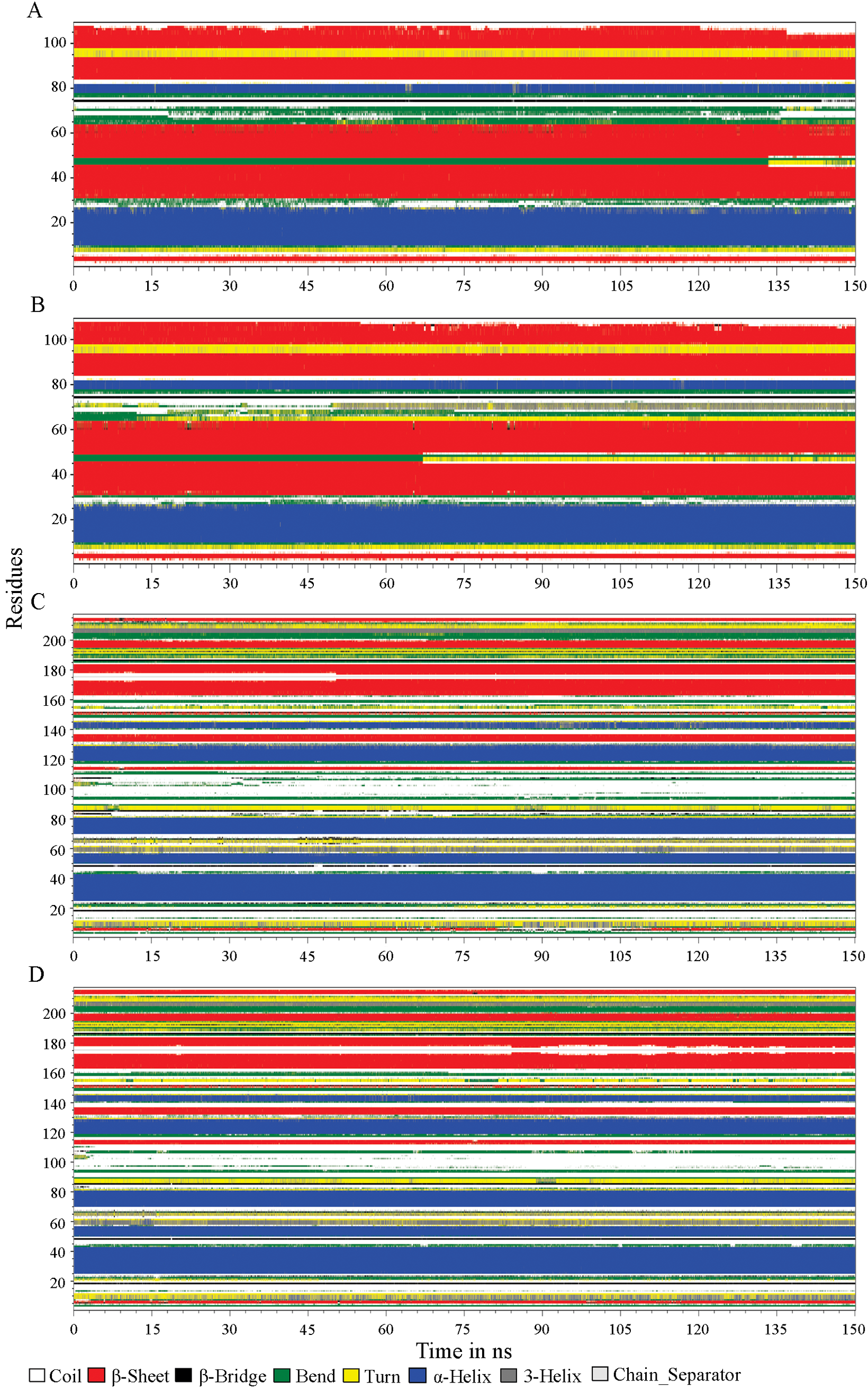

Supplement: S31 Fig — Secondary structure content of cystatin SA in bound (A) and unbound (B) state and that of cathepsin L1 in bound (C) and unbound (D) form. (TIF) [file pone.0164970.s031.tif]

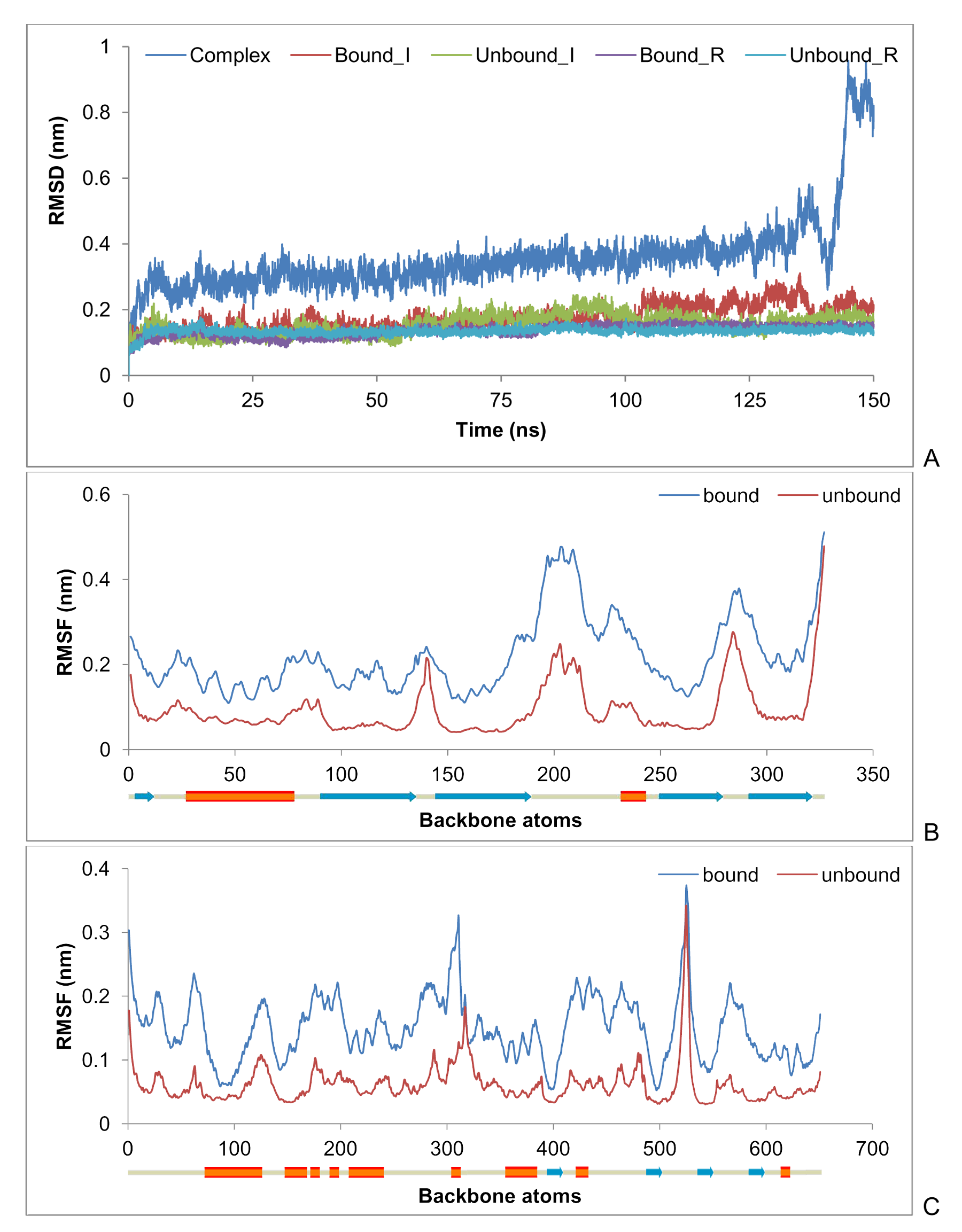

Supplement: S32 Fig — (A) Average backbone RMSD of the complex, inhibitor (I) and receptor (R) in bound and unbound state. RMSF of cystatin SA (B) and cathepsin L1 (C) in complexed form and in free state in solution. (TIF) [file pone.0164970.s032.tif]

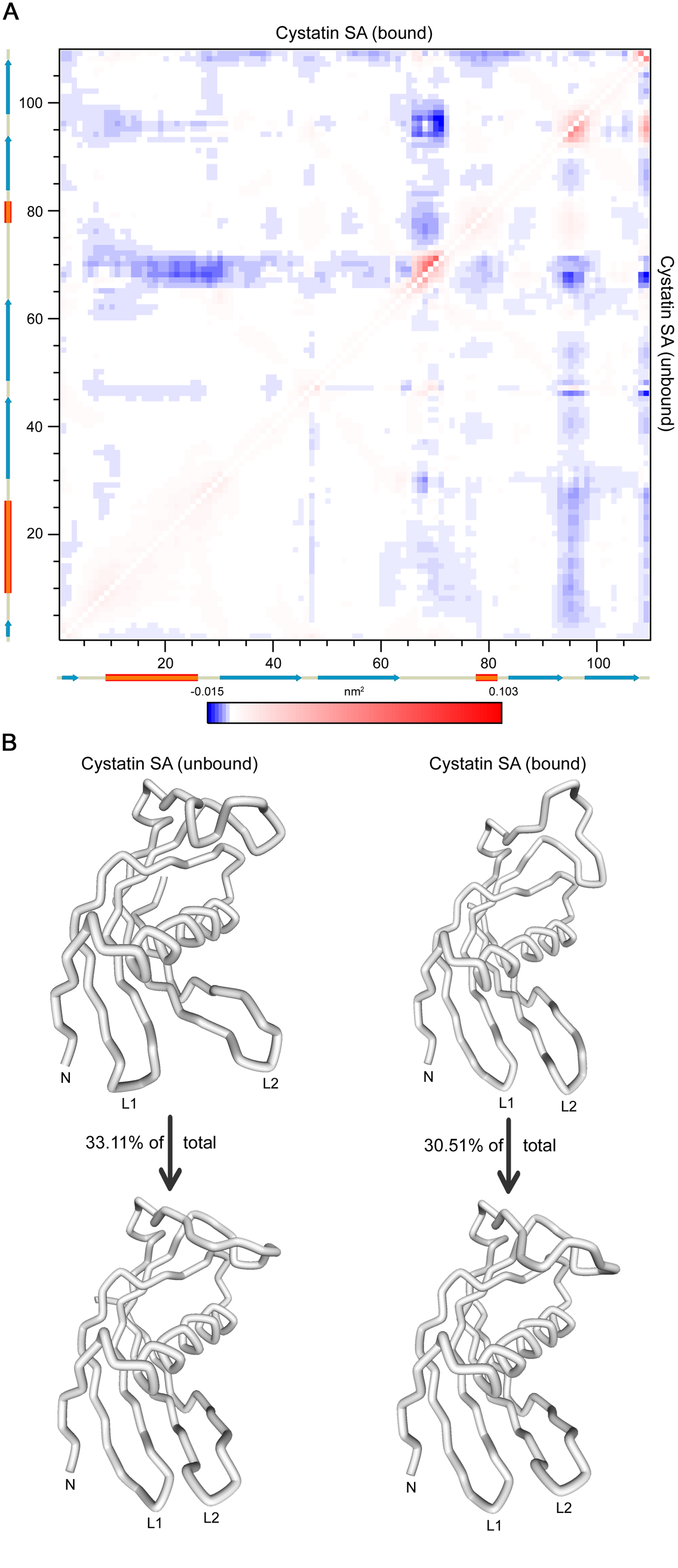

Supplement: S33 Fig — (A) Covariance matrix illustrating correlated and anticorrelated motions of bound (top left) and unbound (bottom right) cystatin SA. The secondary structure of cystatin SA backbone is represented along the axes (from left to right and from bottom to top). (b) Motion of the largest eigenvector of cystatin SA in absence (left) and presence (right) of cathepsin L1. (TIF) [file pone.0164970.s033.tif]

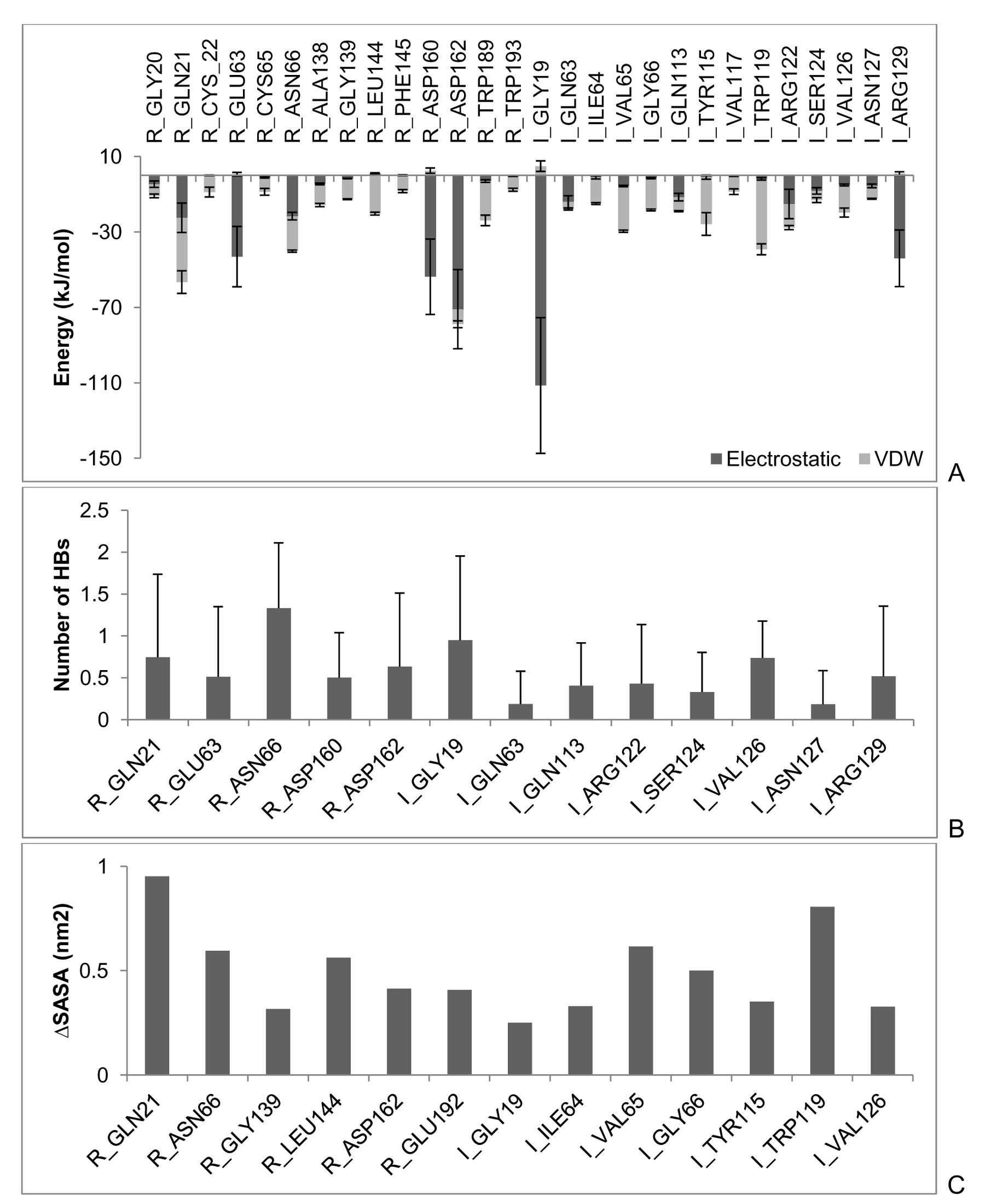

Supplement: S34 Fig — (A) Potential energy of interaction between binding interface residues of cystatin SA (I) & cathepsin L1 (R). Error bars represent the estimated error in GROMACS calculation. (B) Average number of HBs formed among interface residues. Error bars designate standard deviation. (C) Appreciable changes in SASA on complex formation among binding interface residues. (TIF) [file pone.0164970.s034.tif]

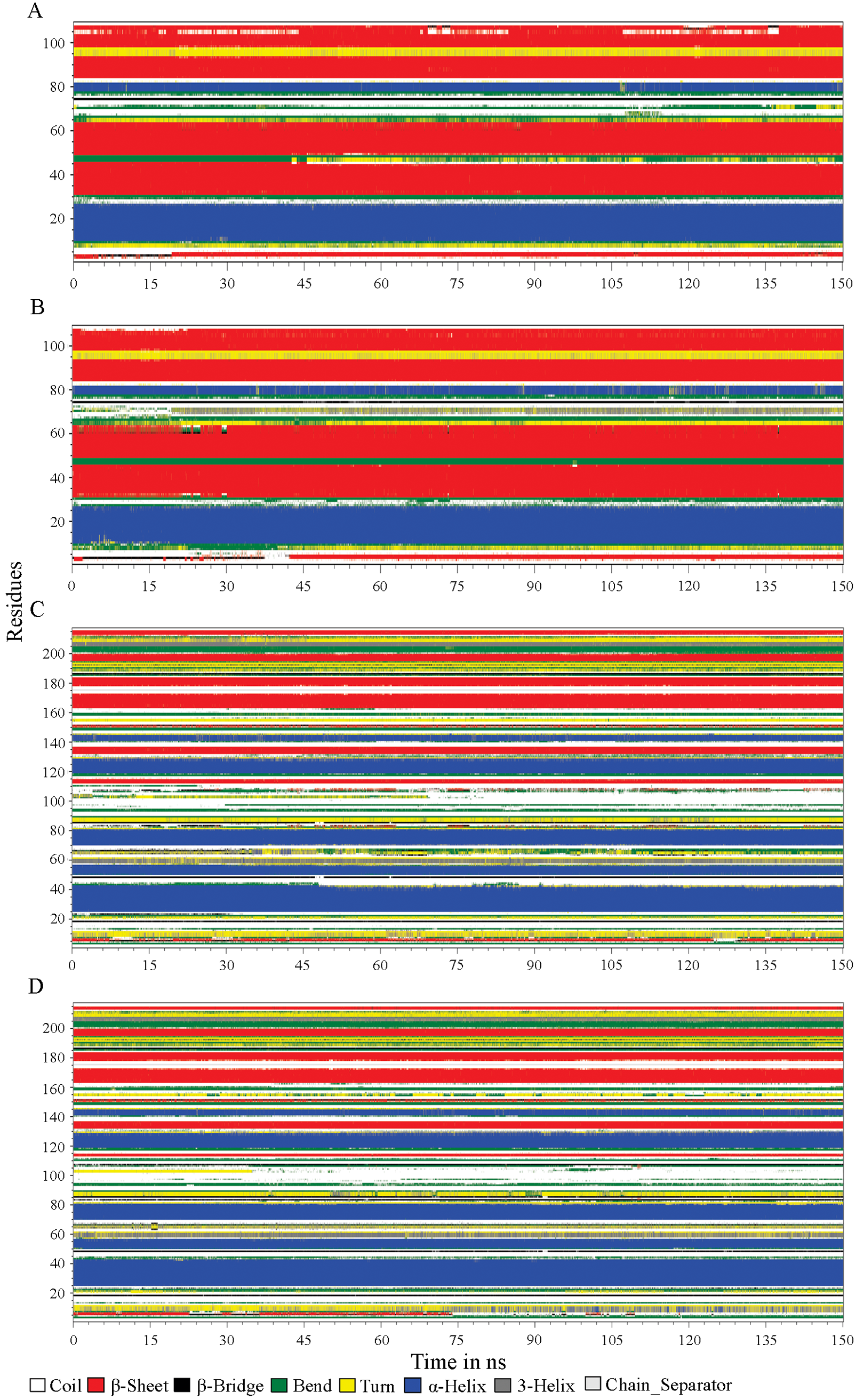

Supplement: S35 Fig — Secondary structure content of cystatin SN in bound (A) and unbound (B) state & that of cathepsin L1 in bound (C) and unbound (D) form. (TIF) [file pone.0164970.s035.tif]

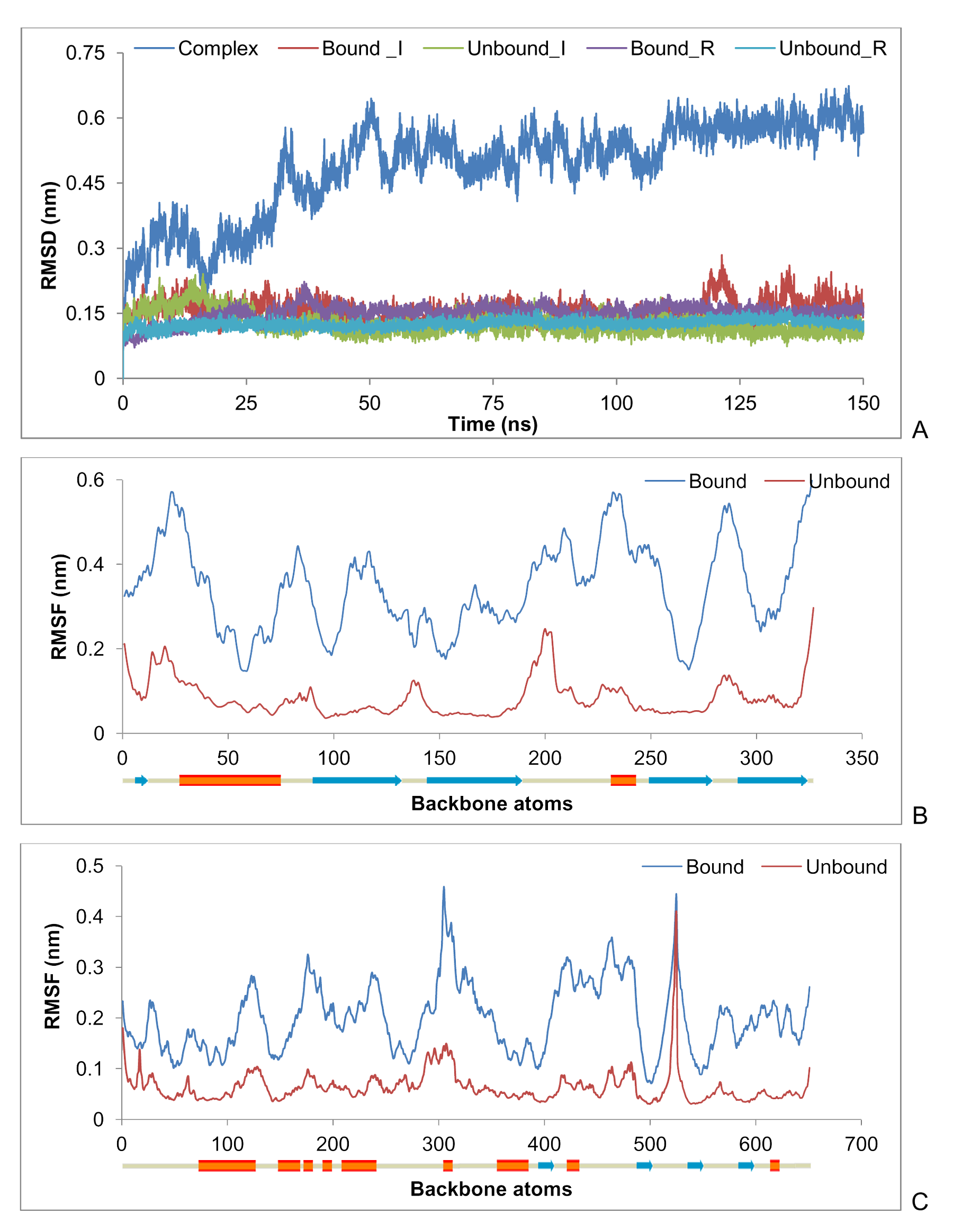

Supplement: S36 Fig — (A) Average backbone RMSD of the complex, inhibitor (I) and receptor (R) in bound and unbound state. RMSF of cystatin SN (B) and cathepsin L1 (C) in complexed form and in free state in solution. (TIF) [file pone.0164970.s036.tif]

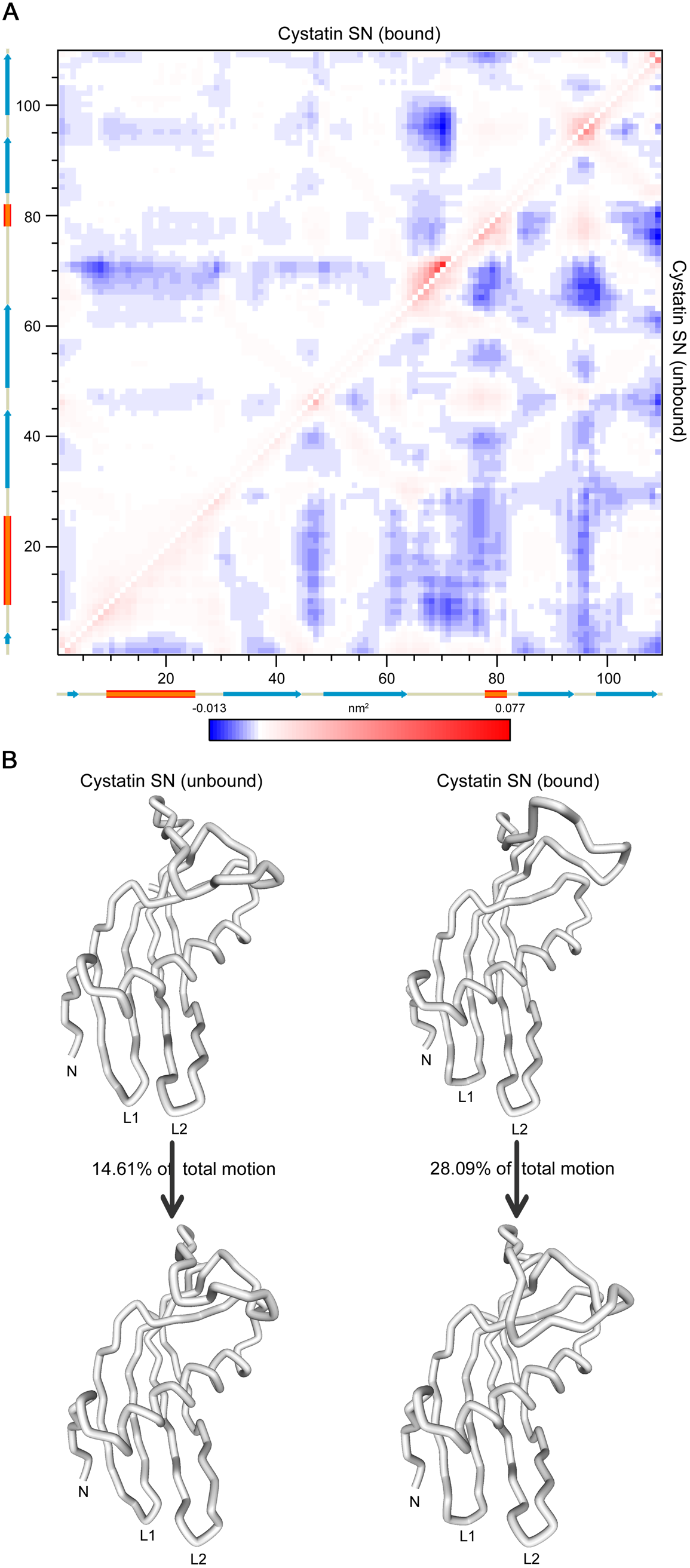

Supplement: S37 Fig — (A) Covariance matrix illustrating correlated and anticorrelated motions of bound (top left) and unbound (bottom right) cystatin SN. The secondary structure of cystatin SN backbone is represented along the axes (from left to right and from bottom to top). (b) Motion of the largest eigenvector of cystatin SN in absence (left) and presence (right) of cathepsin L1. (TIF) [file pone.0164970.s037.tif]

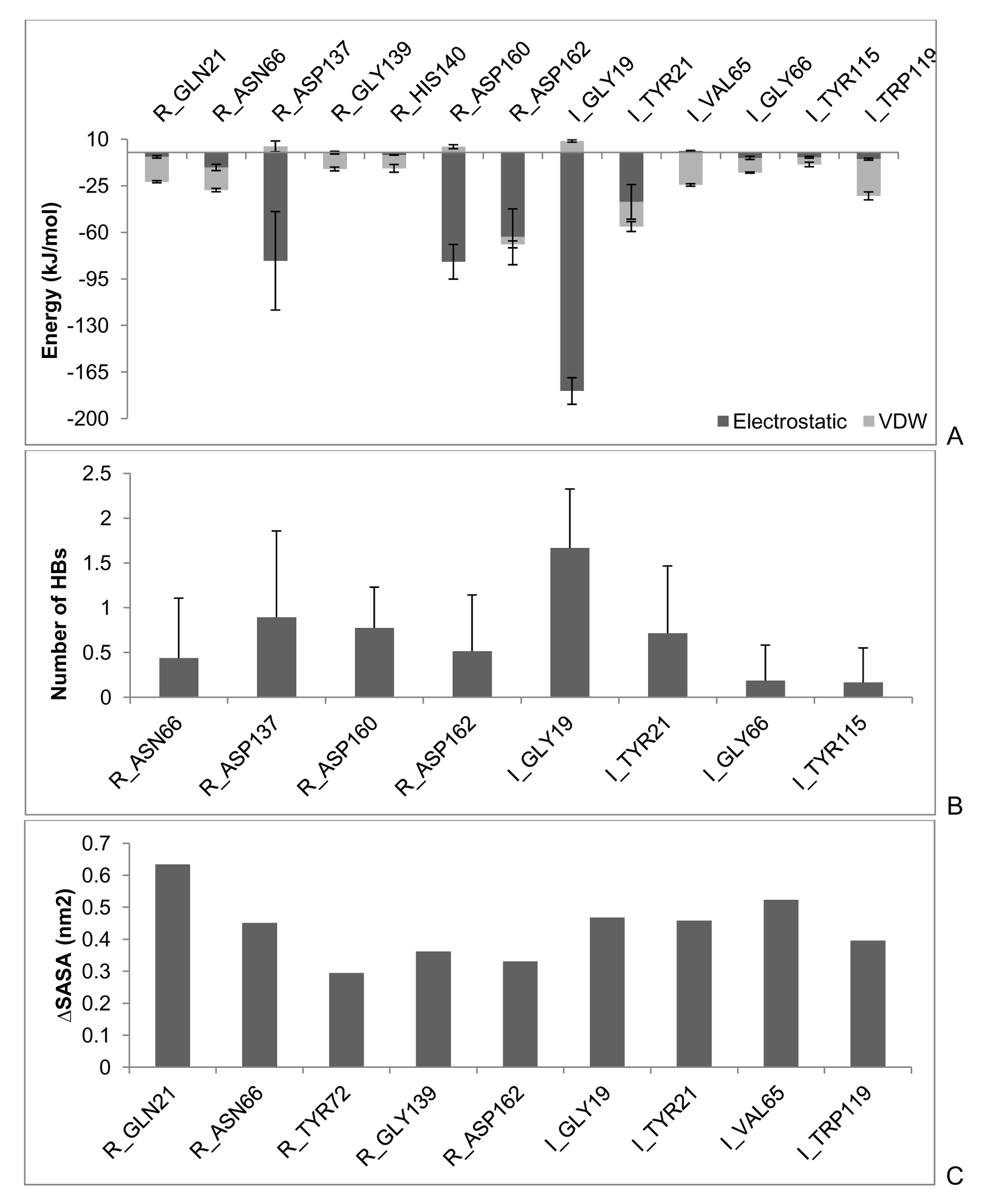

Supplement: S38 Fig — (A) Potential energy of interaction between binding interface residues of cystatin SN (I) & cathepsin L1 (R). Error bars represent the estimated error in GROMACS calculation. (B) Average number of HBs formed among interface residues. Error bars designate standard deviation. (C) Appreciable changes in SASA on complex formation among binding interface residues. (TIF) [file pone.0164970.s038.tif]
