# Supplementary material for: Structural Dynamics Investigation of Human Family 1 & 2 Cystatin-Cathepsin L1 Interaction: A Comparison of Binding Modes
Source: PLoS One. 2016 Oct 20;11(10):e0164970. doi: 10.1371/journal.pone.0164970 (PMC5072729; doi:10.1371/journal.pone.0164970)
Supplement: S1 Table — (DOCX) [file pone.0164970.s039.docx]

**S1 Table.** Structure files preparation.

| **PDB ID** | **Resolution** | **Description** | **Modification** |
| --- | --- | --- | --- |
| 1ICF | 2.00 Å | Crystallographic asymmetric unit contained two copies of cathepsin L1-p41 fragment complex [56]. | Only chain A, B was retained to represent cathepsin L1, other pair is deleted for simplicity. |
| 1NB3 | 2.80 Å | Four steﬁn A–cathepsin H complexes in single asymmetric unit [57]. | Chain I was chosen to represent stefin A. |
| 1STF | 2.37 Å | Stefin B in complex with papain illustrated two mismatches (S3C & Y31E) in Stefin B [*5*]. | Chain I stood for stefin B, mismatches were reversed and subjected to a 2000 step energy minimization with smart minimizer in Discovery Studio (DS). |
| 3GAX | 1.70 Å | Crystal structure of monomeric cystatin C with mutation (L47C & G69C) [58]. | Chain A corresponded cystatin C; mutations were upturned; missing residues were fabricated and minimized likewise [30] |
| 1RN7 | 2.50 Å | Crystal structure of human cystatin D [59]. | Reverse mutated (R26C) to regain the wild type and minimized as before. |
| 2CH9 | 2.10 Å | Crystal structure of human cystatin F [60]. | Hetero-atoms were removed. |

**References** (continued from manuscript)

1. Guncar G, Pungercic G, Klemencic I, Turk V, Turk D. Crystal structure of MHC class II-associated p41 Ii fragment bound to cathepsin L reveals the structural basis for differentiation between cathepsins L and S. EMBO J. 1999; 18: 793-803.
2. Jenko S, Dolenc I, Guncar G, Dobersek A, Podobnik M, Turk D. Crystal structure of Stefin A in complex with cathepsin H: N-terminal residues of inhibitors can adapt to the active sites of endo- and exopeptidases. J Mol Biol. 2003; 326: 875-885.
3. Kolodziejczyk R, Michalska K, Hernandez-Santoyo A, Wahlbom M, Grubb A, Jaskolski M. Crystal structure of human cystatin C stabilized against amyloid formation. FEBS J. 2010; 277: 1726-1737.
4. Alvarez-Fernandez M, Liang YH, Abrahamson M, Su XD. Crystal structure of human cystatin D, a cysteine peptidase inhibitor with restricted inhibition profile. J Biol Chem. 2005; 280: 18221-18228.
5. Schüttelkopf AW, Hamilton G, Watts C, van Aalten DM. Structural basis of reduction-dependent activation of human cystatin F. J Biol Chem. 2006; 281: 16570-16575.
