# Supplementary material for: Structural Dynamics Investigation of Human Family 1 & 2 Cystatin-Cathepsin L1 Interaction: A Comparison of Binding Modes
Source: PLoS One. 2016 Oct 20;11(10):e0164970. doi: 10.1371/journal.pone.0164970 (PMC5072729; doi:10.1371/journal.pone.0164970)
Supplement: S2 Table — (DOCX) [file pone.0164970.s040.docx]

**S2 Table.** Composition of simulated systems.

| **Simulated Systems** | | **Box Size (nm^3^)** | | **Total Number of Atoms** | **Amino Acid Residues** | **Water Molecules** | **Counter-ions** | |
| --- | --- | --- | --- | --- | --- | --- | --- | --- |
|  |  | **Initial** | **Final** |  |  |  | **Na^+^** | **Cl^-^** |
| Stefin A | Complex | (9.03269)^3^ | (9.00447)^3^ | 73714 | 315 | 22942 | 56 | 44 |
|  | Receptor | (7.88602)^3^ | (7.84787)^3^ | 48912 | 217 | 15203 | 40 | 30 |
|  | Inhibitor | (6.50193)^3^ | (6.45284)^3^ | 27325 | 98 | 8578 | 19 | 17 |
| Stefin B | Complex | (9.21883)^3^ | (9.15612)^3^ | 77531 | 315 | 24214 | 57 | 47 |
|  | Receptor | (7.88865)^3^ | (7.86004)^3^ | 49107 | 217 | 15268 | 40 | 30 |
|  | Inhibitor | (6.71342)^3^ | (6.68426)^3^ | 30058 | 98 | 9489 | 18 | 18 |
| Cystatin C | Complex | (10.02782)^3^ | (9.98458)^3^ | 100381 | 326 | 31775 | 70 | 61 |
|  | Receptor | (7.86049)^3^ | (7.88860)^3^ | 49095 | 217 | 15264 | 40 | 30 |
|  | Inhibitor | (7.02178)^3^ | (6.99928)^3^ | 34519 | 109 | 10928 | 21 | 22 |
| Cystatin D | Complex | (10.56587)^3^ | (10.53109)^3^ | 117664 | 329 | 37495 | 81 | 71 |
|  | Receptor | (7.88865)^3^ | (7.86617)^3^ | 49113 | 217 | 15270 | 40 | 30 |
|  | Inhibitor | (7.40548)^3^ | (7.37205)^3^ | 40428 | 112 | 12862 | 24 | 24 |
| Cystatin F | Complex | (10.16056)^3^ | (10.13161)^3^ | 104998 | 343 | 33190 | 68 | 63 |
|  | Receptor | (7.88865)^3^ | (7.86523)^3^ | 49128 | 217 | 15275 | 40 | 30 |
|  | Inhibitor | (8.18083)^3^ | (8.13574)^3^ | 54293 | 126 | 17386 | 33 | 38 |
| Cystatin M/E | Complex | (9.61897)^3^ | (9.60470)^3^ | 89322 | 330 | 28073 | 65 | 54 |
|  | Receptor | (7.88662)^3^ | (7.85814)^3^ | 48963 | 217 | 15220 | 40 | 30 |
|  | Inhibitor | (7.10230)^3^ | (7.07313)^3^ | 35681 | 113 | 11295 | 23 | 22 |
| Cystatin S | Complex | (10.00369)^3^ | (9.95358)^3^ | 99426 | 326 | 31433 | 77 | 60 |
|  | Receptor | (7.88658)^3^ | (7.84973)^3^ | 48969 | 217 | 15222 | 40 | 30 |
|  | Inhibitor | (6.87922)^3^ | (6.82139)^3^ | 32131 | 109 | 10109 | 27 | 20 |
| Cystatin SA | Complex | (10.20243)^3^ | (10.17998)^3^ | 106286 | 326 | 33752 | 70 | 56 |
|  | Receptor | (7.88796)^3^ | (7.85867)^3^ | 49116 | 217 | 15271 | 40 | 30 |
|  | Inhibitor | (6.94055)^3^ | (6.89888)^3^ | 33148 | 109 | 10441 | 24 | 20 |
| Cystatin SN | Complex | (9.75195)^3^ | (9.73041)^3^ | 92875 | 326 | 29245 | 65 | 56 |
|  | Receptor | (7.88525)^3^ | (7.84499)^3^ | 48762 | 217 | 15153 | 40 | 30 |
|  | Inhibitor | (7.04690)^3^ | (6.99761)^3^ | 34670 | 109 | 10947 | 21 | 22 |

Note: Initial & Final designate Box Size before minimization & after simulation, respectively.
