# Supplementary material for: Structural Dynamics Investigation of Human Family 1 & 2 Cystatin-Cathepsin L1 Interaction: A Comparison of Binding Modes
Source: PLoS One. 2016 Oct 20;11(10):e0164970. doi: 10.1371/journal.pone.0164970 (PMC5072729; doi:10.1371/journal.pone.0164970)
Supplement: S3 Table — (DOCX) [file pone.0164970.s041.docx]

**S3 Table.** Pairwise RMSD (Å) of the crystal structure (1ICF), individual trajectory-averages (CL1_A_ - CL1_SN_) and global trajectory-average structure (GT) using mainchain atoms.

| ID | 1ICF | CL1_A_ | CL1_B_ | CL1_C_ | CL1_D_ | CL1_F_ | CL1_ME_ | CL1_S_ | CL1_SA_ | CL1_SN_ | CL1_GT_ |
| --- | --- | --- | --- | --- | --- | --- | --- | --- | --- | --- | --- |
| 1ICF |  |  |  |  |  |  |  |  |  |  |  |
| CL1_A_ | 0.919 |  |  |  |  |  |  |  |  |  |  |
| CL1_B_ | 0.873 | 0.42 |  |  |  |  |  |  |  |  |  |
| CL1_C_ | 1.003 | 0.464 | 0.625 |  |  |  |  |  |  |  |  |
| CL1_D_ | 0.937 | 0.523 | 0.526 | 0.639 |  |  |  |  |  |  |  |
| CL1_F_ | 1.033 | 0.469 | 0.557 | 0.583 | 0.577 |  |  |  |  |  |  |
| CL1_ME_ | 0.912 | 0.484 | 0.535 | 0.572 | 0.45 | 0.59 |  |  |  |  |  |
| CL1_S_ | 0.982 | 0.399 | 0.453 | 0.503 | 0.513 | 0.445 | 0.527 |  |  |  |  |
| CL1_SA_ | 0.882 | 0.56 | 0.569 | 0.588 | 0.598 | 0.586 | 0.468 | 0.505 |  |  |  |
| CL1_SN_ | 0.979 | 0.475 | 0.464 | 0.602 | 0.503 | 0.578 | 0.587 | 0.419 | 0.601 |  |  |
| CL1_GT_ | 0.892 | 0.272 | 0.344 | 0.409 | 0.367 | 0.384 | 0.348 | 0.272 | 0.419 | 0.353 |  |
