# Supplementary material for: Structural Dynamics Investigation of Human Family 1 & 2 Cystatin-Cathepsin L1 Interaction: A Comparison of Binding Modes
Source: PLoS One. 2016 Oct 20;11(10):e0164970. doi: 10.1371/journal.pone.0164970 (PMC5072729; doi:10.1371/journal.pone.0164970)
Supplement: S4 Table — (DOCX) [file pone.0164970.s042.docx]

**S4 Table.** Conformational space normalized overlaps between cathepsin L1 (CL1).

| ID | CL1_A_ | CL1_B_ | CL1_C_ | CL1_D_ | CL1_F_ | CL1_ME_ | CL1_S_ | CL1_SA_ | CL1_SN_ | CL1_GT_ |
| --- | --- | --- | --- | --- | --- | --- | --- | --- | --- | --- |
| CL1_A_ | **0.534** |  |  |  |  |  |  |  |  |  |
| CL1_B_ | 0.574 | **0.523** |  |  |  |  |  |  |  |  |
| CL1_C_ | 0.360 | 0.327 | **0.350** |  |  |  |  |  |  |  |
| CL1_D_ | 0.578 | 0.555 | 0.372 | **0.512** |  |  |  |  |  |  |
| CL1_F_ | 0.562 | 0.555 | 0.353 | 0.544 | **0.352** |  |  |  |  |  |
| CL1_ME_ | 0.575 | 0.586 | 0.352 | 0.595 | 0.540 | **0.534** |  |  |  |  |
| CL1_S_ | 0.559 | 0.572 | 0.377 | 0.558 | 0.560 | 0.559 | **0.523** |  |  |  |
| CL1_SA_ | 0.560 | 0.582 | 0.372 | 0.579 | 0.545 | 0.614 | 0.548 | **0.526** |  |  |
| CL1_SN_ | 0.574 | 0.576 | 0.331 | 0.538 | 0.528 | 0.543 | 0.533 | 0.527 | **0.448** |  |
| CL1_GT_ | 0.495 | 0.449 | 0.643 | 0.517 | 0.495 | 0.493 | 0.520 | 0.492 | 0.471 |  |
| Note: Overlaps among unbound CL1 of different complexes were in regular font, while bold denotes the overlaps among bound and unbound from of CL1 of same complex. | | | | | | | | | | |
