# Supplementary material for: Structural Dynamics Investigation of Human Family 1 & 2 Cystatin-Cathepsin L1 Interaction: A Comparison of Binding Modes
Source: PLoS One. 2016 Oct 20;11(10):e0164970. doi: 10.1371/journal.pone.0164970 (PMC5072729; doi:10.1371/journal.pone.0164970)
Supplement: S5 Table — (DOCX) [file pone.0164970.s043.docx]

**S5 Table.** Structural Properties of unbound CL1s.

| **ID** | **Volume (nm^3^)** | **Density (g/L)** | **Rg (nm)** | **SASA (nm^2^)** |
| --- | --- | --- | --- | --- |
| 1ICF | 36.438 | 1086.440 | 1.662 | 103.947 |
| CL1_A_ | 36.504±1.225 | 1085.716±36.618 | 1.675±0.005 | 112.036 ±1.662 |
| CL1_B_ | 36.407±1.015 | 1088.213±30.478 | 1.670±0.005 | 111.593±1.559 |
| CL1_C_ | 36.645±1.064 | 1081.204±31.383 | 1.679±0.008 | 114.935±2.214 |
| CL1_D_ | 36.423±1.057 | 1087.812±31.667 | 1.674±0.006 | 111.627±1.670 |
| CL1_F_ | 36.606±0.996 | 1082.271±29.517 | 1.677±0.006 | 113.809±1.787 |
| CL1_ME_ | 36.343±1.126 | 1090.334±34.073 | 1.677±0.005 | 112.192±1.538 |
| CL1_S_ | 36.400±1.100 | 1088.562±32.981 | 1.670±0.005 | 111.888±1.537 |
| CL1_SA_ | 36.507±0.943 | 1085.130±28.148 | 1.671±0.007 | 112.913±1.823 |
| CL1_SN_ | 36.508±1.146 | 1085.429±34.176 | 1.669±0.005 | 111.910±1.668 |
| CL1_GT_ | 36.483±1.082 | 1086.075±32.332 | 1.673±0.007 | 112.545±2.036 |
