# Supplementary material for: Structural Dynamics Investigation of Human Family 1 & 2 Cystatin-Cathepsin L1 Interaction: A Comparison of Binding Modes
Source: PLoS One. 2016 Oct 20;11(10):e0164970. doi: 10.1371/journal.pone.0164970 (PMC5072729; doi:10.1371/journal.pone.0164970)
Supplement: S6 Table — (DOCX) [file pone.0164970.s044.docx]

**S6 Table. Structural properties of complexes as a measure of stability.**

| Cathepsin L1 in  complex with: | Rg (nm) | Total SASA (nm^2^) |
| --- | --- | --- |
| Stefin A | 2.08±0.01 | 163.31±2.27 |
| Stefin B | 2.10±0.10 | 164.22±2.19 |
| Cystatin C | 2.26±0.01 | 171.31±2.11 |
| Cystatin D | 2.36±0.02 | 176.32±2.22 |
| Cystatin F | 2.46±0.03 | 194.09±2.95 |
| Cystatin M/E | 2.30±0.02 | 179.91±2.43 |
| Cystatin S | 2.30±0.02 | 176.62±2.46 |
| Cystatin | 2.29±0.03 | 178.68±3.24 |
| Cystatin SN | 2.39±0.05 | 184.49±3.58 |
