# Supplementary material for: Structural Dynamics Investigation of Human Family 1 & 2 Cystatin-Cathepsin L1 Interaction: A Comparison of Binding Modes
Source: PLoS One. 2016 Oct 20;11(10):e0164970. doi: 10.1371/journal.pone.0164970 (PMC5072729; doi:10.1371/journal.pone.0164970)
Supplement: S7 Table — (DOCX) [file pone.0164970.s045.docx]

**S7 Table. Changes in secondary structure after refinement through MDS.**

|  | Cathepsin L1 complex with: | | | | | | | | | | | | | | | | | |
| --- | --- | --- | --- | --- | --- | --- | --- | --- | --- | --- | --- | --- | --- | --- | --- | --- | --- | --- |
|  | Stefin A | | Stefin B | | Cystatin C | | Cystatin D | | Cystatin F | | Cystatin M/E | | Cystatin S | | Cystatin SA | | Cystatin SN | |
| Residues | DC | RS | DC | RS | DC | RS | DC | RS | DC | RS | DC | RS | DC | RS | DC | RS | DC | RS |
| R_VAL5 |  |  |  |  | Sheet | Coil |  |  |  |  |  |  |  |  |  |  |  |  |
| R_ASP6 |  |  |  |  | Sheet | Coil |  |  |  |  |  |  |  |  |  |  |  |  |
| R_TRP7 |  |  |  |  |  |  |  |  |  |  |  |  | Turn | Helix |  |  |  |  |
| R_ARF8 |  |  |  |  |  |  |  |  |  |  |  |  | Turn | Helix |  |  |  |  |
| R_GLU9 |  |  |  |  |  |  |  |  |  |  |  |  | Turn | Helix |  |  |  |  |
| R_LYS10 |  |  |  |  |  |  |  |  |  |  |  |  | Turn | Helix |  |  |  |  |
| R_GLU50 | Helix | Coil |  |  |  |  |  |  | Helix | Coil |  |  |  |  |  |  |  |  |
| R_CYS56 |  |  |  |  | Helix | Turn |  |  |  |  |  |  |  |  |  |  |  |  |
| R_SER57 |  |  | Coil | Helix |  |  |  |  |  |  |  |  |  |  |  |  |  |  |
| R_GLY58 | Turn | Helix |  |  | Turn | Helix | Turn | Helix | Turn | Helix | Helix | Coil |  |  | Turn | Helix | Turn | Helix |
| R_PRO59 | Turn | Helix |  |  | Turn | Helix | Turn | Helix | Turn | Helix | Helix | Turn |  |  | Turn | Helix | Turn | Helix |
| R_GLN60 | Turn | Helix |  |  | Turn | Helix | Turn | Helix | Turn | Helix | Helix | Turn |  |  | Turn | Helix | Turn | Helix |
| R_GLY61 | Turn | Turn |  |  |  |  |  |  |  |  |  |  |  |  |  |  |  |  |
| R_GLY64 |  |  | Helix | Turn | Turn | Helix |  |  | Helix | Turn | Helix | Turn |  |  | Helix | Turn | Helix | Coil |
| R_CYS65 |  |  | Helix | Turn | Turn | Helix |  |  | Helix | Turn | Helix | Turn |  |  | Helix | Turn | Helix | Coil |
| R_ASN66 |  |  | Helix | Coil | Coil | Helix |  |  | Helix | Coil | Helix | Coil |  |  | Helix | Coil | Helix | Coil |
| R_MET70 |  |  |  |  |  |  |  |  |  |  | Helix | Coil |  |  |  |  |  |  |
| R_GLN75 |  |  |  |  | Helix | Turn |  |  |  |  |  |  |  |  |  |  |  |  |
| R_ASN80 |  |  |  |  | Helix | Turn |  |  | Helix | Coil |  |  |  |  |  |  | Helix | Turn |
| R_LEU83 | Sheet | Coil | Sheet | Coil | Sheet | Coil | Sheet | Coil | Sheet | Coil | Sheet | Coil | Sheet | Coil | Sheet | Coil | Sheet | Coil |
| R_ASP84 | Sheet | Coil | Sheet | Coil | Sheet | Coil | Sheet | Coil | Sheet | Coil | Sheet | Coil | Sheet | Coil | Sheet | Coil | Sheet | Coil |
| R_PRO102 | Helix | Coil | Helix | Coil | Helix | Coil | Helix | Coil | Helix | Coil | Helix | Coil | Helix | Coil | Helix | Coil | Helix | Coil |
| R_LYS103 | Helix | Coil | Helix | Coil | Helix | Coil | Helix | Coil | Helix | Coil | Helix | Coil | Helix | Coil | Helix | Coil | Helix | Coil |
| R_TYR104 | Helix | Coil | Helix | Coil | Helix | Coil | Helix | Coil | Helix | Coil | Helix | Coil | Helix | Coil | Helix | Coil | Helix | Coil |
| R_SER105 | Sheet | Coil | Sheet | Coil |  |  | Sheet | Coil | Sheet | Coil | Sheet | Coil | Sheet | Coil | Sheet | Coil | Sheet | Coil |
| R_VAL106 | Sheet | Coil | Sheet | Coil |  |  | Sheet | Coil | Sheet | Coil | Sheet | Coil | Sheet | Coil | Sheet | Coil | Sheet | Coil |
| R_ALA107 | Sheet | Coil | Sheet | Coil |  |  | Sheet | Coil | Sheet | Coil | Sheet | Coil | Sheet | Coil | Sheet | Coil | Sheet | Coil |
| R_ALA127 |  |  | Helix | Turn |  |  |  |  |  |  |  |  |  |  |  |  |  |  |
| R_THR128 |  |  | Helix | Turn |  |  |  |  |  |  |  |  |  |  |  |  |  |  |
| R_VAL129 |  |  |  |  |  |  |  |  |  |  | Turn | Helix | Turn | Helix | Turn | Helix |  |  |
| R_PHE145 |  |  |  |  |  |  |  |  |  |  | Turn | Helix |  |  |  |  |  |  |
| R_GLY169 |  |  |  |  | Sheet | Coil |  |  |  |  | Sheet | Coil |  |  |  |  |  |  |
| R_TYR170 |  |  |  |  | Sheet | Coil |  |  |  |  | Sheet | Coil |  |  |  |  |  |  |
| R_TYR182 |  |  | Sheet | Coil |  |  |  |  |  |  |  |  |  |  |  |  |  |  |
| R_ALA202 |  |  | Sheet | Coil |  |  |  |  |  |  |  |  |  |  |  |  |  |  |
| R_HIS208 |  |  |  |  |  |  |  |  |  |  |  |  | Helix | Turn |  |  |  |  |
| R_CYS209 |  |  |  |  |  |  |  |  |  |  |  |  | Helix | Turn |  |  |  |  |
| R_GLY210 |  |  |  |  |  |  |  |  |  |  |  |  | Helix | Coil |  |  |  |  |
| R_ILE211 |  |  |  |  |  |  |  |  |  |  |  |  | Turn | Turn |  |  |  |  |
| I_ILE20 |  |  |  |  |  |  |  |  |  |  |  |  |  |  | Coil | Sheet |  |  |
| I_LEU20 |  |  |  |  |  |  |  |  |  |  | Coil | Sheet |  |  |  |  |  |  |
| I_PRO20 |  |  |  |  | Sheet | Coil |  |  |  |  |  |  |  |  |  |  |  |  |
| I_TYR21 |  |  |  |  |  |  |  |  |  |  |  |  |  |  |  |  | Coil | Sheet |
| I_ASN22 |  |  |  |  |  |  |  |  |  |  |  |  |  |  |  |  | Coil | Sheet |
| I_ALA23 |  |  |  |  |  |  |  |  |  |  |  |  |  |  | Sheet | Coil |  |  |
| I_THR23 |  |  |  |  |  |  | Sheet | Coil |  |  |  |  |  |  |  |  |  |  |
| I_LEU23 |  |  |  |  |  |  |  |  |  |  | Sheet | Coil |  |  |  |  |  |  |
| I_VAL44 |  |  |  |  |  |  | Turn | Helix |  |  |  |  |  |  |  |  |  |  |
| I_ILE64 |  |  |  |  |  |  |  |  | Sheet | Coil |  |  |  |  |  |  |  |  |
| I_LEU64 |  |  |  |  |  |  |  |  |  |  | Sheet | Coil |  |  |  |  |  |  |
| I_THR64 |  |  |  |  |  |  |  |  |  |  |  |  |  |  |  |  | Sheet | Coil |
| I_ILE68 |  |  |  |  |  |  |  |  |  |  | Sheet | Coil |  |  |  |  |  |  |
| I_VAL68 |  |  |  |  |  |  |  |  |  |  |  |  | Sheet | Coil |  |  |  |  |
| I_LEU80 | Coil | Sheet |  |  |  |  |  |  |  |  |  |  |  |  |  |  |  |  |
| I_THR87 |  |  | Coil | Sheet |  |  |  |  |  |  |  |  |  |  |  |  |  |  |
| I_ASN88 |  |  | Coil | Sheet |  |  |  |  |  |  |  |  |  |  |  |  |  |  |
| I_LYS89 |  |  | Coil | Sheet |  |  |  |  |  |  |  |  |  |  |  |  |  |  |
| I_LEU93 |  |  |  |  |  |  |  |  | Helix | Turn | Coil | Helix |  |  |  |  |  |  |
| I_ASP94 |  |  |  |  |  |  |  |  | Helix | Turn |  |  |  |  |  |  |  |  |
| I_THR94 |  |  |  |  |  |  |  |  |  |  | Turn | Helix |  |  |  |  |  |  |
| I_ASP95 |  |  |  |  |  |  |  |  | Helix | Turn |  |  |  |  |  |  |  |  |
| I_THR95 |  |  |  |  |  |  |  |  |  |  | Turn | Helix |  |  |  |  |  |  |
| I_ALA103 |  |  |  |  |  |  |  |  |  |  | Helix | Coil |  |  |  |  |  |  |
| I_GLN104 |  |  |  |  |  |  |  |  |  |  | Helix | Coil |  |  |  |  |  |  |
| I_GLN105 |  |  |  |  |  |  |  |  |  |  | Helix | Coil |  |  |  |  |  |  |
| I_GLU106 |  |  |  |  |  |  |  |  |  |  | Helix | Coil |  |  |  |  |  |  |
| I_GLU107 |  |  |  |  |  |  | Sheet | Coil |  |  |  |  |  |  |  |  |  |  |
| I_VAL117 |  |  |  |  |  |  | Sheet | Coil |  |  |  |  |  |  |  |  |  |  |
| I_PRO118 |  |  |  |  |  |  | Helix | Turn |  |  |  |  | Turn | Helix |  |  | Turn | Helix |
| I_TRP119 |  |  |  |  |  |  | Helix | Turn |  |  |  |  | Turn | Helix |  |  | Turn | Helix |
| I_GLU120 |  |  |  |  |  |  | Helix | Turn |  |  |  |  | Turn | Helix |  |  | Turn | Helix |
| I_LYS122 |  |  |  |  |  |  | Sheet | Coil |  |  |  |  |  |  |  |  |  |  |
| I_ILE123 |  |  |  |  |  |  | Sheet | Coil |  |  |  |  |  |  |  |  |  |  |
| I_LEU125 |  |  |  |  | Sheet | Coil |  |  |  |  |  |  |  |  |  |  |  |  |
| I_SER126 |  |  |  |  | Sheet | Coil |  |  |  |  |  |  |  |  |  |  |  |  |
| I_TYR1128 |  |  |  |  |  |  | Sheet | Coil |  |  |  |  |  |  |  |  |  |  |
| I_LYS129 |  |  |  |  |  |  | Sheet | Coil |  |  |  |  |  |  |  |  |  |  |
| I_GLN132 |  |  |  |  |  |  |  |  |  |  | Sheet | Coil |  |  |  |  |  |  |
| I_GLU132 |  |  |  |  |  |  |  |  |  |  |  |  |  |  | Sheet | Coil |  |  |
