# Supplementary material for: Structural Dynamics Investigation of Human Family 1 & 2 Cystatin-Cathepsin L1 Interaction: A Comparison of Binding Modes
Source: PLoS One. 2016 Oct 20;11(10):e0164970. doi: 10.1371/journal.pone.0164970 (PMC5072729; doi:10.1371/journal.pone.0164970)
Supplement: S8 Table — (DOCX) [file pone.0164970.s046.docx]

**S8 Table. Reciprocal arrangements of cystatins in Docked & Refined structures.**

| **Cathepsin L1 complex with** | **Distance between centroids (Å)** | **RMSD (Å)** | **Tilt between secondary structures of Cystatin fold in degrees** | | | | | | |
| --- | --- | --- | --- | --- | --- | --- | --- | --- | --- |
|  |  |  | **β1** | **α1** | **β2** | **β3** | **α2** | **β4** | **β5** |
| Stefin A | 2.64 | 1.13 | 90.00 | 36.12 | -55.98 | 46.81 | n.d. | -45.87 | 48.80 |
| Stefin B | 1.88 | 0.78 | 34.88 | 43.20 | -40.88 | 34.32 | n.d. | -39.99 | 50.68 |
| Cystatin C | 3.88 | 1.48 | -62.41 | 72.21 | 13.39 | -43.57 | 86.28 | 86.26 | 58.26 |
| Cystatin D | 6.32 | 1.78 | 7.85 | 68.24 | 24.76 | 52.40 | n.d. | -68.55 | 43.43 |
| Cystatin F | 7.82 | 3.62 | 63.28 | 40.92 | -18.34 | 22.83 | 64.59 | -62.01 | 83.10 |
| Cystatin M/E | 6.27 | 2.41 | 83.08 | 60.17 | 18.35 | -68.89 | n.d. | 83.54 | 54.66 |
| Cystatin S | 8.33 | 1.19 | 87.34 | 89.62 | 35.2 | 82.56 | -48.54 | -29.76 | 14.01 |
| Cystatin SA | 6.59 | 1.38 | 80.78 | 79.40 | 45.92 | 70.18 | -10.89 | -48.17 | 36.77 |
| Cystatin SN | 13.51 | 1.32 | -44.33 | 41.33 | 50.45 | -87.16 | -57.08 | -80.92 | 77.07 |

**Note:** n.d. – not determined as α2 is not present in the docked and reference structure of the said cystatin. (-) sign signifies anti-clockwise movement. Stefins/cystatins are only considered for RMSD calculation.
