# Supplementary material for: Structural Dynamics Investigation of Human Family 1 & 2 Cystatin-Cathepsin L1 Interaction: A Comparison of Binding Modes
Source: PLoS One. 2016 Oct 20;11(10):e0164970. doi: 10.1371/journal.pone.0164970 (PMC5072729; doi:10.1371/journal.pone.0164970)
Supplement: S9 Table — (DOCX) [file pone.0164970.s047.docx]

**S9 Table. Changes in Binding Interface (BI).**

| Cathepsin L1 in complex with: | | | | | | | | | | | | | | | | | |
| --- | --- | --- | --- | --- | --- | --- | --- | --- | --- | --- | --- | --- | --- | --- | --- | --- | --- |
| Stefin A | | Stefin B | | Cystatin C | | Cystatin D | | Cystatin F | | Cystatin M/E | | Cystatin S | | Cystatin SA | | Cystatin SN | |
| DC | RS | DC | RS | DC | RS | DC | RS | DC | RS | DC | RS | DC | RS | DC | RS | DC | RS |
| R_ASN18 | R_ASN18 | R_ASN18 | R_ASN18 | R_GLN19 | R_GLN19 | R_GLN19 | R_GLN19 | R_GLN19 | R_GLN21 | R_GLN19 | R_GLN19 | R_ASN18 | R_GLN19 | R_ASN18 | R_ASN18 | R_GLN19 | R_GLN19 |
| R_GLN19 | R_GLN19 | R_GLN19 | R_GLN19 | R_CYS22 | R_CYS22 | R_GLY20 | R_GLY20 | R_GLY20 | R_CYS22 | R_GLY20 | R_GLN21 | R_GLN19 | R_GLN21 | R_GLN19 | R_GLN19 | R_GLY20 | R_GLY20 |
| R_GLY20 | R_GLY20 | R_GLY20 | R_GLY20 | R_GLY23 | R_GLY23 | R_GLN21 | R_GLN21 | R_GLN21 | R_GLY23 | R_GLN21 | R_GLY23 | R_GLY20 | R_GLY23 | R_GLY20 | R_GLY20 | R_GLN21 | R_GLN21 |
| R_GLN21 | R_GLN21 | R_GLN21 | R_GLN21 | R_SER24 | R_SER24 | R_CYS22 | R_CYS22 | R_CYS22 | R_TRP26 | R_CYS22 | R_CYS25 | R_GLN21 | R_CYS25 | R_GLN21 | R_GLN21 | R_CYS22 | R_CYS22 |
| R_CYS22 | R_CYS22 | R_CYS22 | R_CYS22 | R_CYS25 | R_CYS25 | R_GLY23 | R_GLY23 | R_GLY23 | R_GLU63 | R_GLY23 | R_TRP26 | R_CYS22 | R_TRP26 | R_CYS22 | R_CYS22 | R_GLY23 | R_GLY23 |
| R_GLY23 | R_GLY23 | R_GLY23 | R_GLY23 | R_TRP26 | R_TRP26 | R_CYS25 | R_SER24 | R_SER24 | R_CYS65 | R_SER24 | R_GLY61 | R_GLY23 | R_GLY61 | R_GLY23 | R_GLY23 | R_SER24 | R_CYS25 |
| R_CYS25 | R_SER24 | R_SER24 | R_SER24 | R_GLY61 | R_GLN60 | R_TRP26 | R_CYS25 | R_CYS25 | R_ASN66 | R_CYS25 | R_GLU63 | R_SER24 | R_ASN62 | R_CYS25 | R_SER24 | R_CYS25 | R_ASN66 |
| R_TRP26 | R_CYS25 | R_CYS25 | R_CYS25 | R_ASN62 | R_GLY61 | R_GLU63 | R_TRP26 | R_TRP26 | R_GLY67 | R_TRP26 | R_CYS65 | R_CYS25 | R_GLU63 | R_ASN66 | R_CYS25 | R_TRP26 | R_GLY67 |
| R_CYS65 | R_TRP26 | R_TRP26 | R_TRP26 | R_GLU63 | R_ASN62 | R_CYS65 | R_GLY61 | R_SER29 | R_GLY68 | R_GLY61 | R_ASN66 | R_TRP26 | R_CYS65 | R_ALA138 | R_GLU63 | R_GLU63 | R_GLY68 |
| R_ASN66 | R_ASN62 | R_ASN62 | R_GLU63 | R_CYS65 | R_GLU63 | R_ASN66 | R_ASN62 | R_ALA30 | R_LEU69 | R_GLU63 | R_GLY67 | R_GLY61 | R_ASN66 | R_GLY139 | R_CYS65 | R_CYS65 | R_ASP137 |
| R_GLY67 | R_GLU63 | R_GLU63 | R_CYS65 | R_ASN66 | R_CYS65 | R_GLY67 | R_GLU63 | R_ALA33 | R_MET70 | R_CYS65 | R_GLY68 | R_GLU63 | R_GLY67 | R_HIS140 | R_ASN66 | R_ASN66 | R_ALA138 |
| R_ GLY68 | R_CYS65 | R_GLY64 | R_ASN66 | R_GLY67 | R_ASN66 | R_GLY68 | R_CYS65 | R_GLU63 | R_ASP71 | R_ASN66 | R_LEU69 | R_CYS65 | R_GLY68 | R_GLU141 | R_GLY67 | R_GLY67 | R_GLY139 |
| R_LEU69 | R_ASN66 | R_CYS65 | R_GLY67 | R_GLY68 | R_GLY67 | R_LEU69 | R_ASN66 | R_CYS65 | R_TYR72 | R_GLY67 | R_MET70 | R_ASN66 | R_LEU69 | R_LEU144 | R_ALA93 | R_GLY68 | R_HIS140 |
| R_MET70 | R_GLY67 | R_ASN66 | R_GLY68 | R_LEU69 | R_GLY68 | R_MET70 | R_GLY67 | R_ASN66 | R_PHE74 | R_GLY68 | R_ALA135 | R_GLY67 | R_TYR72 | R_ASP160 | R_GLU95 | R_LEU69 | R_PHE143 |
| R_ALA135 | R_GLY68 | R_GLY67 | R_LEU69 | R_TYR72 | R_LEU69 | R_LYS117 | R_GLY68 | R_GLY67 | R_GLN75 | R_LEU69 | R_ALA138 | R_GLY68 | R_THR94 | R_ASP162 | R_ASP137 | R_MET70 | R_LEU144 |
| R_ALA138 | R_LEU69 | R_GLY68 | R_MET70 | R_ALA135 | R_MET70 | R_VAL134 | R_LEU69 | R_GLY68 | R_ALA93 | R_TYR72 | R_GLY139 | R_LEU69 | R_GLU95 | R_HIS163 | R_ALA138 | R_TYR72 | R_PHE145 |
| R_GLY139 | R_MET70 | R_LEU69 | R_ASP71 | R_ALA138 | R_TYR72 | R_ALA135 | R_MET70 | R_LEU69 | R_THR94 | R_ALA135 | R_HIS140 | R_TYR72 | R_ALA138 | R_SER188 | R_GLY139 | R_ALA135 | R_ASP155 |
| R_GLU141 | R_TYR72 | R_MET70 | R_ALA93 | R_GLY139 | R_ALA135 | R_SER158 | R_TYR72 | R_MET70 | R_GLU95 | R_ASP137 | R_GLU141 | R_ALA138 | R_GLY139 | R_TRP189 | R_PHE143 | R_ILE136 | R_CYS156 |
| R_LEU144 | R_ALA135 | R_TYR72 | R_THR94 | R_HIS140 | R_ILE136 | R_GLU159 | R_ALA135 | R_ASP71 | R_PHE112 | R_ALA138 | R_PHE143 | R_GLY139 | R_HIS140 | R_GLU192 | R_LEU144 | R_ASP137 | R_SER157 |
| R_PHE145 | R_ILE136 | R_ALA93 | R_ALA135 | R_LEU144 | R_ALA138 | R_ASP160 | R_ILE136 | R_TYR72 | R_ASP114 | R_GLY139 | R_LEU144 | R_HIS140 | R_GLU141 | R_TRP193 | R_PHE145 | R_ALA138 | R_ASP160 |
| R_GLU159 | R_ALA138 | R_GLU95 | R_ALA138 | R_GLU159 | R_GLY139 | R_MET161 | R_ALA138 | R_PHE74 | R_LYS117 | R_HIS140 | R_GLU159 | R_LEU144 | R_PHE143 | R_GLY19 | R_ASP160 | R_GLY139 | R_MET161 |
| R_ASP160 | R_GLY139 | R_ALA135 | R_GLY139 | R_ASP160 | R_HIS140 | R_ASP162 | R_GLY139 | R_PHE112 | R_SER133 | R_PHE143 | R_ASP160 | R_ASP160 | R_LEU144 | I_TYR21 | R_ASP162 | R_HIS140 | R_ASP162 |
| R_MET161 | R_GLU141 | R_ILE136 | R_HIS140 | R_MET161 | R_GLU141 | R_HIS163 | R_HIS140 | R_ASP114 | R_VAL134 | R_LEU144 | R_MET161 | R_MET161 | R_MET161 | I_ARG29 | R_HIS163 | R_LEU144 | R_HIS163 |
| R_ASP162 | R_PHE143 | R_ALA138 | R_GLU141 | R_ASP162 | R_PHE143 | R_GLY164 | R_PHE143 | R_LYS117 | R_ALA135 | R_GLU159 | R_ASP162 | R_ASP162 | R_ASP162 | I_GLN63 | R_TRP189 | R_SER157 | R_TRP189 |
| R_HIS163 | R_LEU144 | R_GLY139 | R_LEU144 | R_HIS163 | R_LEU144 | R_SER213 | R_LEU144 | R_SER133 | R_GLY139 | R_ASP160 | R_HIS163 | R_HIS163 | R_HIS163 | I_ILE64 | R_TRP193 | R_GLU159 | I_GLY19 |
| R_GLY164 | R_PHE145 | R_HIS140 | R_PHE145 | R_GLY164 | R_GLU159 | R_ALA214 | R_GLU159 | R_VAL134 | R_GLU141 | R_MET161 | R_GLY164 | R_TRP189 | R_GLY164 | I_VAL65 | I_GLY19 | R_ASP160 | I_TYR21 |
| R_SER188 | R_TYR146 | R_GLU141 | R_LYS147 | R_TRP189 | R_ASP160 | I_ALA17 | R_ASP160 | R_ALA135 | R_GLU159 | R_ASP162 | R_TRP189 | I_TYR21 | R_TRP189 | I_GLY66 | I_GLN63 | R_MET161 | I_TRP29 |
| R_TRP189 | R_ASP160 | R_PHE143 | R_MET161 | I_GLY19 | R_MET161 | I_GLY18 | R_MET161 | R_ILE136 | R_ASP160 | R_HIS163 | I_GLU19 | I_TRP11 | R_GLY19 | I_GLY67 | I_ILE64 | R_ASP162 | I_GLN62 |
| R_GLU192 | R_MET161 | R_LEU144 | R_ASP162 | I_PRO20 | R_ASP162 | I_GLY19 | R_ASP162 | R_ASP137 | R_MET161 | R_GLY164 | I_ARG21 | I_THR64 | I_ILE20 | I_VAL68 | I_VAL65 | R_HIS163 | I_GLN63 |
| R_TRP193 | R_ASP162 | R_PHE145 | R_HIS163 | I_MET21 | R_HIS163 | I_HIS21 | R_HIS163 | R_ALA138 | R_ASP162 | R_SER188 | I_GLN29 | I_PHE65 | I_TYR21 | I_GLN113 | I_GLY66 | R_GLY164 | I_THR64 |
| I_MET1 | R_HIS163 | R_LYS147 | R_GLY164 | I_GLU27 | R_GLY164 | I_TYR61 | R_GLY164 | R_GLY139 | R_ALA214 | R_TRP189 | I_SER62 | I_GLY66 | I_GLN63 | I_TYR115 | I_GLY67 | R_TRP189 | I_VAL65 |
| I_ILE2 | R_GLY164 | R_MET161 | R_SER188 | I_LYS62 | R_TRP189 | I_GLN63 | R_TRP189 | R_HIS140 | R_ALA215 | R_ALA214 | I_GLN63 | I_GLY67 | I_THR64 | I_GLU116 | I_VAL68 | R_ALA214 | I_GLY66 |
| I_PRO3 | R_SER188 | R_ASP162 | R_TRP189 | I_GLN63 | R_ALA214 | I_ILE64 | R_ALA214 | R_GLU141 | R_SER216 | I_GLU19 | I_LEU64 | I_VAL68 | I_PHE65 | I_VAL117 | I_ASN69 | I_GLY19 | I_GLY67 |
| I_GLY4 | R_TRP189 | R_HIS163 | R_GLU192 | I_ILE64 | I_GLY19 | I_VAL65 | I_ALA17 | R_PHE143 | I_THR6 | I_ARG21 | I_VAL65 | I_ASN69 | I_GLY66 | I_TRP119 | I_PHE71 | I_TYR21 | I_TYR115 |
| I_GLY5 | R_GLU192 | R_GLY164 | R_TRP193 | I_VAL65 | I_PRO20 | I_GLY66 | I_GLY18 | R_LEU144 | I_CYS7 | I_LEU23 | I_ALA66 | I_GLU113 | I_GLY67 | I_GLU120 | I_GLN113 | I_ALA23 | I_TRP119 |
| I_LEU6 | R_TRP193 | R_TRP189 | R_SER213 | I_ALA66 | I_MET21 | I_GLY67 | I_GLY19 | R_ASP155 | I_GLN9 | I_ASP27 | I_GLY67 | I_TYR115 | I_VAL68 | I_ARG122 | I_TYR115 | I_ASP27 |  |
| I_SER7 | R_GLY194 | R_GLU192 | R_ALA214 | I_GLY67 | I_GLU27 | I_VAL68 | I_GLN62 | R_CYS156 | I_SER13 | I_GLN29 | I_ILE68 | I_VAL117 | I_ASN69 | I_MET123 | I_GLU116 | I_TRP29 |  |
| I_GLU15 | I_MET1 | R_TRP193 | I_MET1 | I_VAL68 | I_LYS62 | I_ASN115 | I_GLN63 | R_SER157 | I_ARG14 | I_SER62 | I_LYS69 | I_PRO118 | I_PHE71 | I_SER124 | I_VAL117 | I_GLN62 |  |
| I_THR45 | I_ILE2 | R_ALA214 | I_MET2 | I_ASN69 | I_GLN63 | I_GLU116 | I_ILE64 | R_SER158 | I_VAL15 | I_GLN63 | I_TYR70 | I_TRP119 | I_GLU113 | I_VAL126 | I_PRO118 | I_GLN63 |  |
| I_GLN46 | I_PRO3 | I_MET1 | I_CYS3 | I_PHE71 | I_ILE64 | I_VAL117 | I_VAL65 | R_GLU159 | I_LYS16 | I_LEU64 | I_GLU113 | I_GLU120 | I_TYR115 |  | I_TRP119 | I_THR64 |  |
| I_VAL47 | I_GLY4 | I_MET2 | I_GLY4 | I_GLN113 | I_VAL65 | I_PRO118 | I_GLY66 | R_ASP160 | I_PRO17 | I_VAL65 | I_LEU115 | I_ASP121 | I_PRO118 |  | I_GLU120 | I_VAL65 |  |
| I_VAL48 | I_GLY5 | I_CYS3 | I_ALA5 | I_TYR115 | I_ALA66 | I_TRP119 | I_GLY67 | R_MET161 | I_GLY18 | I_ALA66 | I_VAL117 | I_ARG122 | I_TRP119 |  | I_ARG122 | I_GLY66 |  |
| I_ALA49 | I_LEU6 | I_GLY4 | I_PRO6 | I_PRO118 | I_GLY67 | I_GLU120 | I_ASN69 | R_ASP162 | I_PHE19 | I_GLY67 | I_PRO118 | I_SER124 | I_GLU120 |  | I_MET123 | I_GLY67 |  |
| I_GLY50 | I_SER7 | I_ALA5 | I_LYS44 | I_TRP119 | I_VAL68 | I_LYS122 | I_TYR71 | R_HIS163 | I_LYS21 | I_ILE68 | I_TRP119 | I_VAL126 | I_ASP121 |  | I_SER124 | I_VAL68 |  |
| I_THR51 | I_GLU15 | I_PRO6 | I_GLN46 | I_GLN120 | I_ASN69 | I_ILE123 | I_GLN113 | R_GLY164 | I_ILE64 | I_LYS69 | I_GLN1120 |  | I_ARG122 |  | I_VAL126 | I_ASN69 |  |
| I_ASN52 | I_THR45 | I_GLN46 | I_VAL47 | I_SER126 | I_TYR70 | I_SER124 | I_ASN115 | R_ASN187 | I_VAL65 | I_TYR70 | I_ASN121 |  | I_VAL126 |  | I_ASN127 | I_TYR70 |  |
| I_TYR53 | I_GLN46 | I_VAL47 | I_VAL48 |  | I_PHE71 | I_LEU126 | I_VAL117 | R_TRP189 | I_LYS66 | I_LEU115 | I_GLN124 |  | I_ASN127 |  | I_ARG129 | I_GLU113 |  |
| I_TYR54 | I_VAL47 | I_VAL48 | I_ALA49 |  | I_GLN113 |  | I_PRO118 | R_GLY210 | I_VAL117 | I_VAL117 | I_LEU126 |  |  |  |  | I_TYR115 |  |
| I_LYS68 | I_VAL48 | I_ALA49 | I_GLY50 |  | I_PRO118 |  | I_TRP119 | R_SER213 | I_PRO118 | I_PRO118 | I_LYS127 |  |  |  |  | I_VAL117 |  |
| I_PHE70 | I_ALA49 | I_GLY50 | I_THR51 |  | I_TRP119 |  | I_GLU120 | R_ALA214 | I_TRP119 | I_TRP119 |  |  |  |  |  | I_PRO118 |  |
| I_LYS71 | I_GLY50 | I_THR51 | I_ASN52 |  |  |  | I_ASP121 | R_ALA215 | I_LEU120 | I_GLN120 |  |  |  |  |  | I_TRP119 |  |
| I_SER72 | I_THR51 | I_ASN52 | I_PHE54 |  |  |  | I_LEU126 | R_SER216 | I_GLN121 | I_ASN121 |  |  |  |  |  | I_GLU120 |  |
| I_LEU73 | I_TYR54 | I_ARG68 | I_ARG68 |  |  |  |  | R_TYR217 |  | I_SER122 |  |  |  |  |  | I_ARG122 |  |
| I_PRO74 | I_LYS68 | I_PHE70 | I_PHE70 |  |  |  |  | I_SER8 |  | I_GLN124 |  |  |  |  |  | I_SER124 |  |
| I_GLY75 | I_PHE70 | I_GLN71 | I_LEU73 |  |  |  |  | I_GLN9 |  | I_LEU126 |  |  |  |  |  | I_VAL126 |  |
| I_GLN76 | I_LYS71 | I_LEU73 | I_PRO74 |  |  |  |  | I_ASP10 |  |  |  |  |  |  |  |  |  |
| I_GLU78 | I_SER72 | I_PRO74 | I_HIS75 |  |  |  |  | I_LEU11 |  |  |  |  |  |  |  |  |  |
| I_VAL81 | I_LEU73 | I_HIS75 | I_GLU76 |  |  |  |  | I_ASN12 |  |  |  |  |  |  |  |  |  |
| I_THR83 | I_PRO74 | I_SER83 | I_SER83 |  |  |  |  | I_SER13 |  |  |  |  |  |  |  |  |  |
| I_GLY97 | I_GLY75 | I_THR96 | I_THR96 |  |  |  |  | I_ARG14 |  |  |  |  |  |  |  |  |  |
| I_PHE98 | I_THR83 | I_TYR97 | I_TYR97 |  |  |  |  | I_VAL15 |  |  |  |  |  |  |  |  |  |
|  |  |  |  |  |  |  |  | I_LYS16 |  |  |  |  |  |  |  |  |  |
|  |  |  |  |  |  |  |  | I_PRO17 |  |  |  |  |  |  |  |  |  |
|  |  |  |  |  |  |  |  | I_GLY18 |  |  |  |  |  |  |  |  |  |
|  |  |  |  |  |  |  |  | I_PHE19 |  |  |  |  |  |  |  |  |  |
|  |  |  |  |  |  |  |  | I_PRO20 |  |  |  |  |  |  |  |  |  |
|  |  |  |  |  |  |  |  | I_LYS21 |  |  |  |  |  |  |  |  |  |
|  |  |  |  |  |  |  |  | I_ILE23 |  |  |  |  |  |  |  |  |  |
|  |  |  |  |  |  |  |  | I_PRO28 |  |  |  |  |  |  |  |  |  |
|  |  |  |  |  |  |  |  | I_GLY29 |  |  |  |  |  |  |  |  |  |
|  |  |  |  |  |  |  |  | I_VAL62 |  |  |  |  |  |  |  |  |  |
|  |  |  |  |  |  |  |  | I_GLN63 |  |  |  |  |  |  |  |  |  |
|  |  |  |  |  |  |  |  | I_ILE64 |  |  |  |  |  |  |  |  |  |
|  |  |  |  |  |  |  |  | I_VAL65 |  |  |  |  |  |  |  |  |  |
|  |  |  |  |  |  |  |  | I_LYS66 |  |  |  |  |  |  |  |  |  |
|  |  |  |  |  |  |  |  | I_GLY67 |  |  |  |  |  |  |  |  |  |
|  |  |  |  |  |  |  |  | I_LEU68 |  |  |  |  |  |  |  |  |  |
|  |  |  |  |  |  |  |  | I_TRP115 |  |  |  |  |  |  |  |  |  |
|  |  |  |  |  |  |  |  | I_VAL117 |  |  |  |  |  |  |  |  |  |
|  |  |  |  |  |  |  |  | I_PRO118 |  |  |  |  |  |  |  |  |  |
|  |  |  |  |  |  |  |  | I_TRP1119 |  |  |  |  |  |  |  |  |  |
|  |  |  |  |  |  |  |  | I_LEU120 |  |  |  |  |  |  |  |  |  |
|  |  |  |  |  |  |  |  | I_GLN121 |  |  |  |  |  |  |  |  |  |
|  |  |  |  |  |  |  |  | I_HIS122 |  |  |  |  |  |  |  |  |  |
|  |  |  |  |  |  |  |  | I_GLU124 |  |  |  |  |  |  |  |  |  |
| **Note:** DC – Docked Complex, RS – Reference Structure, R-Receptor, I-Inhibitor. Red colored residues are unique either to DC or RS of the concerned complex. | | | | | | | | | | | | | | | | | |
