# Supplementary material for: Structural Dynamics Investigation of Human Family 1 & 2 Cystatin-Cathepsin L1 Interaction: A Comparison of Binding Modes
Source: PLoS One. 2016 Oct 20;11(10):e0164970. doi: 10.1371/journal.pone.0164970 (PMC5072729; doi:10.1371/journal.pone.0164970)
Supplement: S10 Table — (DOCX) [file pone.0164970.s048.docx]

**S10 Table. Flexibility of bound and unbound cystatins.**

| **Cathepsin L1-Stefin/Cystatin Complexes** | **Trace Values in nm^2^** | |
| --- | --- | --- |
|  | **Bound State** | **Unbound state** |
| Stefin A | 0.469 | 0.865 |
| Stefin B | 0.423 | 1.402 |
| Cystatin C | 0.800 | 1.009 |
| Cystatin D | 0.946 | 1.546 |
| Cystatin F | 2.281 | 3.971 |
| Cystatin M/E | 2.991 | 2.346 |
| Cystatin S | 0.928 | 0.871 |
| Cystatin SA | 1.270 | 1.321 |
| Cystatin SN | 0.897 | 0.495 |
