# Supplementary material for: Structural Dynamics Investigation of Human Family 1 & 2 Cystatin-Cathepsin L1 Interaction: A Comparison of Binding Modes
Source: PLoS One. 2016 Oct 20;11(10):e0164970. doi: 10.1371/journal.pone.0164970 (PMC5072729; doi:10.1371/journal.pone.0164970)
Supplement: S1 Text — (DOCX) [file pone.0164970.s049.docx]

**Simulation results of Cathepsin L1-Stefin/Cystatin complexes**

In the following sub-sections, discussion on the results of MDS was organized according to cathepsin L1 (CL1)-cystatin complexes. First the dynamics of cystatins and its binding partner were compared in bound and unbound state and then the specific intermolecular interactions of stefin/cystatin-CL1 pair were talked about.

**Stefin A–Cathepsin L1 complex**

The stability of stefin A and CL1 during MDS was assessed through DSSP profile (S3 Fig). In bound state, a short consistent β-bridge was observed in stefin A near N-terminal; and fluctuations among bend, turn and coil were almost nonexistent in L1 (residues 46-50) & L2 (residues 72-79) loop. Further a 3% increase in β-sheets and concomitant decrease in coil structure was observed in bound state. The receptor protein (CL1) did not show any appreciable distinction between bound and unbound state.

The RMSD of the complex was stabilized ≈20 ns but exhibited variation over the bound and unbound state of stefin A and CL1 (S4A Fig). Bound stefin A registered lower average backbone RMSD compared to its free state in solution. On the other hand, the receptor showed a slightly lower average backbone RMSD in unbound state. These two results resembled well with its DSSP profile (S3 Fig).

A root-mean-square ﬂuctuation (RMSF) analysis revealed, (S4B, C Fig) except the conserved regions of stefin A that directly participated in interaction with CL1, viz. N-terminal (residues 0-5), L1 loop (residues 46-50), L2 loop and partly the fourth β sheet (residues 70-79), all other part fluctuated more in the bound state compared to the free protein in solution (S4B Fig). The bound and unbound state of CL1 illustrated minor differences in terms of RMSF. The loop regions of CL1 including residues 103-110 and residues near chain break (175), recognized as disordered region, fluctuated more in complexed form and contributed to the RMSD plot. Whereas the regions directly participating in interaction (residues 19-26, 65-69, 160-163, 189-193) with stefin A fluctuated less in bound state (S4C Fig).

S5A Fig showed a covariance matrix for the two states of stefin A - bound to CL1 (top left) and in absence of CL1 (bottom right). Stefin A in its bound state experienced large areas of both correlated and anti-correlated motion compared to the unbound state. Coordinated motions were observed between two β hairpin loops, N-terminal and L1 loop, L2 loop and C-terminal. These observations were in accordance with earlier RMSF results – say, overall higher fluctuation of the inhibitor at bound state and notably lower fluctuation of three conserved regions participating in interaction (S4B Fig). A total of 294 vectors were generated to describe the trajectory illustrating a normalized conformational space overlap of 0.512. Top 9 eigenvectors described more than 70% of the overall system motion for the unbound trajectory while first 17 eigenvectors accounted for 71% of collective motions of complexed stefin A. The bound inhibitor reported lower flexibility in terms of trace values of covariance matrix (S10 Table) and thus stabilization of the complex - seconded earlier RMSD & RMSF results. In synchronization of these observations, the largest eigenvector of the unbound inhibitor represented 33.7% of total motion whereas that of bound inhibitor was credited for mere 13.89% of total motion (S5B Fig). The three conserved regions fell apart from each other in the unbound state, L2 loop moved away, but reform a wedge shaped structure when bound to CL1. In both case the α1 helix was adjusted accordingly - anti-correlated motions between α1 helix & L2 loop can be noted in both bound and unbound state, while L1 loop withdraws its correlated motion with α1 helix in bound state (S5A Fig).

The major contributing factors that stabilized the interaction between stefin A and CL1 were summarized in S6A Fig. The bound state was dominated by VDW interaction providing 72% of the IE. The N-terminal residues (39% of IE) and the L1 loop (35% of IE) contributed almost equally and were the main players in stefin A-CL1 interaction, whereas L2 loop provided 24% of IE, C-terminal end (2% of IE) also recorded its presence. All three conserved regions chiefly acted as VDW interaction centre, only at C-terminal VDW and electrostatic interaction were found to be equally essential. The importance of N-terminal, especially of the residue Pro3 [9] and relatively smaller contribution of the second binding loop [12] were in agreement with earlier studies, which validated the model and opened the path for new observations about the dynamics of the inhibitor in bound and unbound state. Met1, Pro3, Gly4, Val47, Val48, Ala49, Lys71 of stefin A and Gln21, Asn66, Leu144, Asp162 Trp189 of CL1 demonstrated large negative potential energies of interaction; Pro3 contributed the largest. Pro3, Gly4 and Ala49 of stefin A formed persistent inter molecular HBs with Gly68, Asp 162 and Gly20 of the receptor (S6B Fig). Met1 of stefin A also formed favorable electrostatic contacts with the receptor residues Asp160, Met161 and Asp162. N-terminal residues Met1, Ile2, Pro3, Gly4, conserved L1 loop residues Val47, Val48, Ala49 and Leu73, Pro74 of L2 loop of stefin A formed important VDW contacts with the hydrophobic core near active site of CL1, comprising residues Cys22, Cys25, Leu69, Ala135, Ala138, Leu144, Phe145, Met161. Other receptor residues such as Gln19, Gln21, Gly23, Trp26, Asn66, Gly67, Gly68, Asp162, His163, Trp189, and Trp193 also set up some important VDW contacts with the inhibitor. The residue wise significant changes in ∆SASA (i.e. difference in SASA in bound and unbound state of the receptor and inhibitor protein), on complex formation were also shown in S6C Fig. Some key residues such as Asn66, Asp162 in receptor & Pro3, Gly4, Ala49, Lys71 of inhibitor could be identified, which not only reported high IE and but also participated in both hydrogen bonding and VDW interaction.

**Stefin B–Cathepsin L1 complex**

DSSP analysis elucidated the stability of stefin B and CL1 in complex and in free state (S7 Fig). In case of inhibitor, stabilization of L1 loop (residues 48-50), increase of coil conformation with subsequent decrease in bend and turn were observed at C-terminal and L2 loop region (residues 72-80) in bound state. Gain in β-sheet was also noticed around 63 ns at bound state near β1 (residues 7-9). 2% increase in overall secondary structure of the bound receptor was found; associated with loss of coil (2%) and β-bridge (1%) and concomitant gain in β-sheet (2%) and turn (1%).

Average backbone RMSD of stefin B in bound state exhibited marked stability; the bound inhibitor got stabilized almost at once while the unbound one takes ≈100 ns to reach steady state (S8A Fig). The receptor in the bound state showed more deviation compared to unbound state and almost followed the RMSD of stefin B-CL1 complex. RMSD of both, the complex and the bound CL1, got stabilized around 15 ns and remained so till 75 ns, after which both showed a gradual increase up to 100 ns and stabilized thereafter. The RMSF analysis identified the region (residues 99-112) exhibited enhanced fluctuation in bound state, also spotted as disordered region, and responsible for the increase in RMSD in both cases after 75 ns (S8A, C Fig). Loss of β-bridge in bound state and abolition of bend conformation after 78 ns in bound state of the receptor was also linked with the same region in DSSP profile (S7C, D Fig). The bound receptor fluctuated more than the unbound one; the regions directly involved in interaction (residues 19-23, 64-68, 160-161) and residues 173-175 were the only exception (S8C Fig). The inhibitor also illustrated more fluctuation in bound state, with stabilization of interacting regions, to wit N-terminal (residues 1-7), L1 loop & portion of β2 & β3 (residues 44-53), L2 loop & part of β4 & β5 (residues 67-79, 82-83) and C-terminal segment (residues 87-98) (S8B Fig).

The bound stefin B was also more populated with correlated and anti-correlated motions compared to unbound state, coped with RMSF results (S8B, S9A Fig), shared a conformational space overlap of 0.326. Correlated motions between N-terminal & L1 loop was observed only in bound state while anti-correlated movements between N-terminal & L2 loop and correlated motions between L1 & L2 loop were detected in both bound and unbound state. The unbound protein showed more flexibility compared to the bound one (S10 Table). First 7 eigenvectors accounts for 72% of total motion in unbound state whereas top 17 eigenvectors were needed to describe similar amount of motion in bound state. The largest principal component (PC) (28.89% of total motion) of unbound stefin B described closing of three conserved sites through displacement of L1 loop by N-terminal, depicted as an anti-correlated motion between the two in covariance matrix. In contrast, the largest PC (15.78% of total motion) of stefin A in complex with CL1 described minor adjustments among three conserved regions needed for accommodating themselves within the catalytic core (S9A, B Fig).

The important interactions between stefin B and CL1 were reported in S10 Fig. Here also, the major portion (60%) of IE was provided by VDW contacts. The N-terminal region was the principal contributor of binding energy, providing 45% of IE, whereas L1 loop, L2 loop and C-terminal provided 23%, 21% and 11% of IE respectively. The N-terminal and C-terminal ends participated almost equally in both VDW and electrostatic interaction, but L1 & L2 loop chiefly acted as VDW interaction center. Receptor residues Gln21, Asn66, Asp71, and inhibitor residues Met1, Cys3, Tyr97, depicted large negative potential energies of interaction (S10A Fig). These observations, namely relative importance of conserved regions and role of Met1, Met2, Cys3, Tyr97 for endo-peptidases, went well with the published literature [5, 10, 14*,* 54] and validated the model. The N-terminal residue Cys3 of stefin B and Gly68 of CL1, formed two consistent HBs with each other’s backbone carbonyl oxygen; Met1 formed HBs with receptor residue Met70 & Asp71; N-terminal Gly4 & L1 loop residues Gln46, Ala49 remained persistently hydrogen bonded with the backbone carbonyl oxygen of CL1 residues Asp162, Cys65 & Gly20 correspondingly; Tyr97 formed multiple HBs with receptor residues Gln21 & Asn66 and Gln21 as well did the same with inhibitor residues Asn52 & Ser83 (S10B Fig). Apart from hydrogen bonding, Arg68 and His75 of stefin B formed favorable electrostatic contacts with Gln21 and Leu144, Tyr146 respectively. Receptor residue Gly67 in addition interacted electrostatically with N-terminal residues (1-6) and Gln46 of L1 loop. The N-terminal (residue 1-6) made favorable VDW contacts with receptor residues 23, 25, 26, 65-70, 135, 161-164; L1 loop residues (46-50) showed VDW interaction with residues 19-23, 65-67, 144, 189 of CL1; L2 loop (residues 70-75) built VDW contact with CL1 residues 21, 144, 147, 189, 192, 193; and the C-terminal residue Tyr97 formed VDW contacts with residues 21-23, 65, 66. Most of the residues participated in the VDW contact undergo appreciable change in SASA, which further confirmed their participation in the interaction (S10A, C Fig). Receptor residues Gln21, Asn66, Gly68, Asp162, and Met1, Cys3, Gly4, Ala49, His75, Tyr97 of stefin B were found to contribute in both electrostatic and VDW interactions and recorded high IE as well.

**Cystatin C–Cathepsin L1 complex**

DSSP profile demonstrated overall stability of secondary structural elements in both cystatin C and CL1 (S11 Fig). The L1 loop (residues 46-49), AS loop (residues 66-77) and the 2^nd^ α-helix (78-81) of cystatin C showed less fluctuation in bound state. An overall 2% decrease in secondary structure content of cystatin C was noted in bound state, as turn was replaced by bend in all three regions (L1, AS & α2), and further complemented by a minute increment in β-sheet content. The average backbone RMSD of the complex was stabilized around 80 ns after initial fluctuations (S12A Fig). Cystatin C RMSD in bound and unbound state almost followed each other, although the bound state moved ahead almost throughout the course with a marked discrepancy around 48 ns; whereas the receptor showed marked stabilization when bound with cystatin C. The consistently higher RMSD of the unbound receptor after 30 ns (S12A Fig) was due to the higher fluctuation of the disordered region residues 101-111, which was further confirmed from RMSF analysis, where the unbound receptor fluctuated a bit less on the whole, with a noted exception for the residues 103-111 (S12C Fig). The same region was characterized with occasional β-bridge and consistent bend conformation in bound state instead coil conformation that prevailed in the unbound protein, which was further supported by the decrease in coil and consequent increase in β-bridge in bound state of CL1 (S11C, D Fig). For cystatin C, except the L1 (residues 44-48) & L2 (residues 92-96) loop, the rest of the residues indicated higher fluctuation in bound state (S12B Fig).

The unbound protein showed more flexibility compared to the bound one (S10 Table) with an overlap of 0.583. The covariance matrix was characterized with larger number of collective motions when cystatin C remained in complex with CL1, also recognized in RMSF studies (S13A, S12B Fig). The N-terminal region and L2 loop of bound cystatin C were engaged in correlated motions with L1 loop while L2 loop and α2 helix portrayed correlated motions in both bound and unbound state. The first 9 eigenvectors in unbound state described 71% of total collective motion while top 12 eigenvectors did the same in bound state. In absence of CL1, the major eigenvector (24.74% of total motion) portrayed outward motion of L2 loop and subsequent inward motion of AS loop as seen from anti-correlated and correlated motions of L1, L2 and AS Loop (S13A, B Fig). The very motion was dropped to 21.53% in bound state characterized with major inward movement of AS loop and minor adjustments of three conserved regions – intense anti-correlated motion between AS & L2 loop could also be noted (S13A, B Fig).

The interacting residues of cystatin C and CL1 were described in S14A Fig. VDW interaction holds major share (≈68%) in IE; L1 & L2 loop served as VDW interaction centre, while N-terminal residues established electrostatic & VDW contacts equally. The L1 loop of the inhibitor provided 68% of IE, N-terminal and L2 loop contributed 19% and 13% of IE respectively. This initial finding corresponded well with previous estimations that the first hairpin loop was responsible for ≈60% of the energy of binding of cystatin C to papain and cathepsin B; and likewise comparable contribution of N-terminal and second hairpin loop along with importance of the conserved Trp residue in L2 loop for papain, actinidin, cathepsins B and H binding [13]. Gly19, Gln63, Ile64, Val65, Ala66 of cystatin C and Asp162, Gly68 of CL1 exhibited large negative potential energies of interaction. Ile64 and Gly67 of cystatin C made HB with Gly68 and Asp162 of CL1 respectively throughout the simulation (S14B Fig). The L1 loop residues, Lys62, Gln63, Val65 and Ala66 of the inhibitor formed favorable electrostatic contacts with Gly23, Cys25, Glu63, Asn66, Gly67, Gly68, Asp160, Asp162 of CL1; N-terminal Gly19 of cystatin C electrostatically interacted with Gln60 and Tyr72 of the receptor (S14A Fig). Further N-terminal cystatin C residues 19-21, established VDW contacts with receptor residues 69, 72, 161; L1 loop residues (62-68) set up strong VDW interaction with residues 25, 26, 63, 66-69, 160-163 of CL1; the two highly conserved L2 loop residues (PW) also took part in VDW interaction with receptor residues 139, 144 & 189. The conserved Trp residue provided almost half (49%) of the L2 loops share in binding free energy, which was also in accordance with previous observations [13]. These interactions were further validated by major changes in SASA (S14A, C Fig). Moreover, few key residues such as Gly68, Asp162 of CL1 and Gly19, Ile64 of cystatin C could be seen to have high IE, as well noting their presence in both HB & SASA analysis.

**Cystatin D–Cathepsin L1 complex**

DSSP analysis recorded overall stability of the secondary structures of cystatin D and CL1 (S15 Fig). The 5^th^ β sheet of the inhibitor at the bound state was split into β-sheet and β-bridge linked by coil, appeared after 36 ns and lasted throughout the simulation (S15A Fig). The RMSF profile also spotted increased fluctuation for C-terminal residues of the inhibitor in bound state (S16B Fig); as well simultaneous decrease in sheet structure and increase in coil conformation led to overall loss of secondary structure by 2% (13A, B Fig). The RMSD of cystatin D-CL1 complex as well that of cystatin D varied throughout the simulation irrespective of its bound or unbound state. In CL1, the bound and unbound state almost followed each other up to 90 ns; after that the receptor in complexed form showed a little more deviation (S16A Fig). The RMSF analysis of the inhibitor indicated greater fluctuation in bound state except in some N-terminal (1-3) and L1 residues (47-50), that took part in interaction with receptor (S16B Fig). In receptor also, the bound state fluctuates more, only two regions (residues 41-44 & 107-110) were seen to fluctuate less than its unbound state (S16C Fig). Both regions also demonstrated more consistent appearance of secondary structures at DSSP profile in bound state, manifestation of β-bridge was also observed (S15C, D Fig).

The covariance matrix of cystatin D was notably less populated with collective motions in absence of binding partner (S17A Fig) showing a normalized overlap of 0.521 between bound and unbound state. In bound state, L2 loop showed correlated motion with L1 & AS loop, similar motions were also observed between N-terminal & L1 loop (S17A Fig). Out of 336 eigenvectors that defined the total motion of the system, first 7 eigenvectors described >70% of total motion in unbound state and top 9 eigenvectors were needed to do the same in bound state. The unbound inhibitor appeared to be more flexible compared to the bound one (S10 Table). The largest PC (23.75% of total motion) in bound state illustrated slight reorientation of the conserved sites and big inward movement of post helix region and AS loop towards the wedge shaped association, reflected in covariance matrix analysis, as well (S17A, B Fig). The highest PC (29.96% of total motion) of the unbound inhibitor also portrayed the same motion with a flip of N-terminal region.

Key interactions between cystatin D and CL1 were outlined in S18 Fig. VDW interactions (68% of IE) dominated over the electrostatic one and prevailed in all three interaction sites; L1 loop hold the biggest part (61% of IE), L2 Loop (33% of IE) and N-terminal (6% of IE) offered the rest. Residues Asn66, Gly68, Asp162 of the receptor and Ile64, Val65, Gly66, Trp119 of the inhibitor displayed large negative potential energy of interaction (S18A Fig). Val65 of cystatin D and Gly68 of CL1 formed persistent dual HBs with each other’s backbone carbonyl oxygen and amide nitrogen; inhibitor residue Gly66 built HB with backbone carbonyl oxygen of Asp162; Gln63 remained hydrogen bonded with Asp160 of CL1; receptor residue Asn66 set up multiple HBs with Asn69 & Asn115 of cystatin D; Gln113 made HB with receptor residue Glu63 (S18B Fig). Further Gly67 of CL1 established favorable electrostatic contacts with L1 loop (residues 64-67 & 69). In L1 loop of cystatin D, VDW interactions were organized centering Val65 along with Ile64 & Gly66, which interacts with receptor residues 23, 25, 26, 63, 67-69, 135, 161-164; and the highly conserved Trp residue of L2 loop formed VDW contacts with residues 162 & 163 of CL1. The appreciable changes in SASA further illustrated this fact (S18A, C Fig). Still there were some residues inevitable in all kind of interaction such as Gln63, Val65, Gly66 of cystatin D and Glu63, Asn66, Asp162 of CL1, might hold the key of cystatin D-CL1 interaction.

**Cystatin F–Cathepsin L1 complex**

DSSP analysis showed conservation of secondary structure in both receptor and inhibitor throughout the range of simulation (S19 Fig). Cystatin F exhibited marked stabilization at the bound state in average backbone RMSD analysis; whereas the bound and unbound states of CL1 remained almost inseparable in RMSD plot until 53ns, then the bound receptor showed a steep increase and stabilized thereafter (S20A Fig). The highly fluctuating random coil dominated disordered region (residues 95-110) in the receptor was mainly responsible for the deviation in bound state (S20C Fig). The DSSP profile in addition evidenced ≈65 ns residues 95 – 107 in bound state took random coil conformation in contrary to the bend and consistent β-bridge of the unbound receptor (S19C, D Fig). The deviation observed during 126–144 ns in bound cystatin F in RMSD analysis was resulted from varying N-terminal and AS loop structure (S20A, C and S21A Fig). Around same period, transformation of bend to coil conformation at N-terminal was noted in secondary structure content of bound state (S19A, B Fig). The RMSD of the complex accumulated the peculiarities of both bound inhibitor and receptor; initial stabilization was followed by increase ≈55 ns, stabilized again and showed some discrepancy ≈120 ns (S20A Fig). In RMSF analysis, both the bound receptor and inhibitor exhibited more fluctuation except the interaction hubs, viz. N-terminal (residues 1-11), L1 loop residues (59-60) and portions of 4^th^ and 5^th^ β sheet (residues 101-105 & 114-118) of inhibitor (S20B, C Fig).

Most of the correlated and anti-correlated motions in both bound and unbound state were localized around N-terminal, which points towards the importance of N-terminal in complex formation. Other two conserved regions were involved in correlated motions within them in both bound and unbound state (S21A Fig). The unbound cystatin F exhibited more flexibility compared to the bound one (S10 Table), reported conformational overlap of 0.472. First 3 eigenvectors of unbound cystatin F described >70% of the total motion while top 4 eigenvectors did so for the bound inhibitor. The top PC of unbound cystatin F reported folding back of N-terminal and consequent motions of AS loop and post helix region, accounted for 49.39% of total motion. The first PC of the bound inhibitor explained 30.67% of total motion, where the N-terminal was extended to occupy with the catalytic core of CL1 (S21B Fig).

The IE profile of cystatin F-CL1 expressed large dominance of N-terminal region providing 73% of IE, while L1 and L2 loop shared 17% and 10% of IE. Moreover, here electrostatic interaction (73% of IE) prevailed over the VDW (27% of IE) one and except L2 loop, both N-terminal and L1 loop mainly acted as electrostatic interaction center although the former also set up some VDW contacts. Glu63, Asp71, Asp114, Asp160, Met161 of CL1 and inhibitor residues Thr6, Arg14, Lys16, Lys66, and Trp119 demonstrated large negative potential energy of interaction (S22A Fig). Each of these residues mainly participated in electrostatic interactions, except Met161, which depicted almost equal electrostatic and VDW potential energy and the conserved Trp119 of L2 loop which chiefly acted as a VDW interaction centre. Arg14 made three persistent HB with Asp71, Ala214 & Ser216 of CL1; Lys16 formed two HBs, one with MET161, other was distributed over Glu159 & Asp160; Thr6 participated in hydrogen bonding with Asp71 & Asp114 while Lys66 did the same with Glu63 & Asn66 of the receptor (S22B Fig). Furthermore, Lys21 & Trp119 of cystatin F built electrostatic contacts with receptor residues Gly139 & Asn66 respectively. Cystatin F-CL1 complex also documented an intra-chain salt bridge between receptor residue Glu63 and Lys66 of cystatin F. Among the two VDW interaction centre, the N-terminal one (residues 7, 14-17) interacted with receptor residues 69, 70, 72, 160-162 and the L2 loop centre (residues 118-119) set up VDW contacts with residues 21-23, 65 & 66 of CL1. The same is also evidenced in ∆SASA analysis (S22A, C Fig). Further residues Asn6, Asp71, Asp160, Met161 of CL1 and Thr6, Arg14, Lys16 of cystatin F scored highly in IE, formed HBs and VDW contacts as well.

**Cystatin M/E–Cathepsin L1 complex**

DSSP study evidenced in general conservation of secondary structures in both cystatin M/E & CL1 in bound and unbound state (S23 Fig). The secondary structure assignment of extended AS loop region (residues 64-86) somewhat differed in bound & unbound state and also exhibited more fluctuations in bound state as seen from RMSF analysis; the second α-helix emerged for a brief period at the initial stages of simulation in the unbound form (S23A, B and S24B Fig). The average backbone RMSD of CL1 demonstrated little difference in bound and unbound state but the inhibitor fluctuate much more throughout the simulation period. The bound inhibitor showed noticeable stabilization compared to the unbound one after 115 ns (S24A Fig). The complex RMSD resembled well with that of bound inhibitor, depicted stabilization ≈60 ns. RMSF analysis of cystatin M/E portrayed less fluctuation in bound state for regions that interacted with CL1, such as N-terminal (residues 1-3), L1 loop (residues 44-49), L2 loop and adjacent β-sheets (residues 96-99, 102-105) (S24B Fig). CL1 fluctuated little bit more in bound state; although exhibited less fluctuation at binding sites (residues 63-69 & 159-162) (S24C Fig).

The covariance matrix showed less collective motions in bound state compared to the unbound state (S25A Fig) with a normalized conformational space overlap of 0.527. In both bound and unbound state the elongated AS loop acts as a center of collective motions; correlated motions between N-terminal & L1 loop, both correlated & anti-correlated motions at L2 loop were also detected (S25A Fig). Here the bound cystatin showed more flexibility than the unbound one (S10 Table). Top 3 PC of bound cystatin M/E demonstrated 70.71% of total motion while 6 largest eigenvectors of the unbound inhibitor described 72.15% of total motion. The largest PC in bound state credited for 46.58% of the total motion, portrayed formation of wedge shaped structure as three conserved regions came close with an inward flip of the extended AS loop. In unbound state the first PC rendered the same motion in reverse order, the AS loop flip back and conserved regions falls apart, attributed 31.12% of the total motion (S25B Fig).

The IE profile of cystatin M/E–CL1 complex (S26A Fig) was dominated by electrostatic interactions (61% of IE); N-terminal and L1 loop served as electrostatic interaction centers, although L2 loop primarily established VDW contacts. L1 loop emerged as major contributor (55%) of IE, N-terminal came second - credited for 27% of IE and L2 loop supplied the rest (18% of IE). Glu63, Glu159, Asp160, Asp162 of CL1 and Arg21, Ala66, Lys69, Trp119 of cystatin M/E surfaced as major interacting residues. Arg21 made multiple HBs with Asp160 & Glu159 of CL1; Ala 66 also formed multiple HBs with the receptor residue Gly68; Gly67 built HB with backbone carbonyl oxygen of CL1 residue Asp162; Lys69 remained hydrogen bonded with receptor residue Glu63 (S26B Fig). Ile68 and Lys127 of cystatin M/E further set up electrostatic contacts with Gly67, Asp162 and Glu63 of CL1, respectively. The conserved residues of L1 loop (residues 64-68) and L2 loop (residues 118-120) of cystatin M/E formed VDW contacts with residues 25, 26, 66-69, 161-164 and 138, 139, 144, 162, 163, 189 of CL1, respectively. Residues Leu115 & Leu126 of 4^th^ & 5^th^ β sheet of cystatin M/E established favorable VDW contacts with Asn66. Large changes in SASA of Asn66, Asp162 of CL1 and Leu64, Val65, Ala66 & Trp119 further proved their involvement in VDW interactions (S26C Fig). Receptor residues Glu63, Gly68, Asp160, Asp162 and Arg21, Ala66, Gly67, Lys69 of cystatin M/E not only scored high IE but also named their presence in both HB & VDW contacts.

**Cystatin S–Cathepsin L1 complex**

DSSP profiles portrayed overall conservation of cystatin S and CL1 secondary structure with few distinct features (S27 Fig). The 1^st^ β sheet of cystatin S fluctuated between β sheet and β-bridge structure in bound state and later regained its sheet structure. The receptor on the other hand, recorded only 1% decrease in secondary structure when bound to cystatin S coupled with similar decrease in sheet structure. The average backbone RMSD of both inhibitor and receptor was slightly lower in their bound form compared to the unbound state (S28A Fig). While the inhibitor was stabilized around 70 ns in bound form, the receptor exhibited regular pattern throughout the simulation and the complex reached steady state around 80 ns after initial gradual increase and fall. In RMSF analysis, the bound form illustrated added fluctuation for both receptor & inhibitor. The L1 loop residues (45-46) of cystatin S and the disordered loop region (residues 107-109) of CL1 registered values lower than unbound state (S28B, C Fig).

The covariance matrix reported higher number of collective motions in complexed form in comparison to the unbound one, which was also seconded by the RMSF results (S29A and S28B Fig). Bound cystatin S showed correlated motion between L1 loop & N terminal, α2 & L2 loop, and both correlated & anti-correlated motions between L1 & L2 loop. The bound cystatin S exhibited minutely higher flexibility than the unbound one (S10 Table) with a normalized overlap of 0.546. Top 8 PC of bound cystatin S accounted for >71% of total motion, whereas the first 10 eigenvectors of unbound inhibitor were needed to describe similar amount of motion. The largest eigenvector of bound cystatin S (34.49% of total motion) illustrated that three conserved regions approached each other to form the wedge and flipping out of AS loop; the top PC of unbound inhibitor (20.57% of total motion) portrayed slight reorientation of three conserved regions and AS loop (S29B Fig).

The interaction contour of cystatin S–CL1 was mainly governed by VDW contacts (78% of IE). L1 and L2 loop played the key role in interaction by providing 52% & 42% of IE, while N-terminal was responsible for 6% of the interaction. L1 loop mainly took part in VDW contacts formation but also participated in electrostatic interactions, N-terminal just did the opposite and L2 loop solely acted as VDW interaction centre. Asn66 of CL1 and Phe65, Gly66, Asn69 and Trp119 of cystatin S reported large negative potential energy of interaction (S30A Fig). Asn69 formed a consistent HB with Asn66 of CL1 and Gly68 of the receptor made HB with backbone carbonyl oxygen of Phe65 of cystatin S (S30B Fig). The L1 loop residues (65, 66 and 69) of cystatin S established favorable electrostatic and VDW contacts with receptor residues 66-68 & 161 and 162. The highly conserved Pro118 & Trp119 of cystatin S established VDW contact with receptor residues 138-140, 144, 162-163 & 189. Tyr115 also set up VDW contacts with residues 23 & 66 of CL1. ∆SASA analysis also confirmed these residues as VDW interaction centre (S30C Fig). The L1 loop (residues 65-67) and adjacent β-sheet residues (63, 69 and 71) also made VDW contacts with residues 23, 63, 66-69 and 161-163 of CL1. Few key residues for interaction such as Asn66 of CL1, Phe65, Asn69 of cystatin S were evidenced to participate in all type of interaction and recorded high IE.

**Cystatin SA–Cathepsin L1 complex**

Overall conservation of secondary structures of receptor and inhibitor was noticed in DSSP profile (S31 Fig). The conformation of L1 loop (residues 45-48) was transformed from bend to a mixture of turn, bend and coil in both bound and unbound state of cystatin SA after ≈132 ns and 66 ns, respectively. Loss of sheet structure near C-terminal end also occurred around similar time and the second α-helix was noticed in both bound and unbound state; turn remained almost absent in AS loop region (residues 65-77) in bound state, appeared only for a brief period ≈138 ns. The receptor recorded 1% loss in secondary structure in bound state mainly attributed to β-bridge; loss of β-sheet near chain break (residue 175) was restored in bound state. Average backbone RMSD profile of receptor was almost same in bound and unbound state; while the both states of inhibitor almost followed each other upto 100 ns, then the deviation in bound state increased and again got stabilized around 140 ns (S32A Fig). The divergence was mostly due to AS loop (residues 66-83), which also showed highest fluctuation in RMSF analysis, whereas the regions interacting with receptor, L1 (residues 45-48) & L2 loop (residues 94-97) demonstrated less fluctuation in bound state (S32B Fig). This peculiar behavior of AS loop as evidenced by DSSP ≈135 ns was also responsible for the marked variation in complex RMSD (S31A, B and S32A, B Fig). The bound receptor illustrated more fluctuation comparative to unbound state (S32C Fig).

The covariance matrix reported correlated motions of L1 loop with both N-terminal & L2 loop in bound state and both coordinated and uncoordinated motions between β-hairpin loops in unbound state (S33A Fig). The bound and unbound inhibitor was hardly distinguishable in terms of flexibility, showed an overlap of 0.509 (S10 Table). First 7 eigenvectors of both bound and unbound inhibitors were credited for >70% of motion. The largest PC (33.11 % of total motion) of unbound cystatin SA represented teaming up of three conserved regions and subsequent adjustment of AS loop & post helix region. In case of bound inhibitor, the top PC (30.51% of total motion) characterized minor reorientation of conserved regions with a major inward flip of AS loop (S33B Fig).

In cystatin SA–CL1 interaction electrostatic and VDW contacts played almost equally important roles by providing nearly 53% & 47% of IE. L2 loop dominated the interactions by contributing 53% of IE, both VDW and electrostatic contacts were observed in 60:40 ratio. N-terminal and L1 loop participated evenly by furnishing 26% & 21% of IE, although N-terminal contribution was largely dominated by electrostatic interaction, VDW contacts prevailed in the L1 loop participation. The major contributors of IE were enlisted in S34A Fig. Gln21, Glu63, Asn66, Asp160, Asp162 of CL1 and Gly19, Trp119, Arg129 of cystatin SA recorded considerable negative potential energy of interaction. Gly19 remained hydrogen bonded with Asp160 & Asp162 of CL1; Arg129 formed HB with Glu63 of the receptor; Asn66 of CL1 established multiple HBs with Val126, Gln113 & Asn127 of cystatin SA; receptor residue Gln21 built HBs with Arg122 & Ser124; Asp162 of the receptor also participated in hydrogen bonding with Gln63 (S34B Fig). The L1 loop residues (64-66) set up VDW contacts with receptor residues 138, 139, 144, 145, 162, 189 and 193. The L2 loop and adjacent β-sheet residues (113, 115, 117, 119, 122, 126, 127) also made favorable VDW contacts with CL1 residues 19-22, 65, 66 & 189. Most of these residues also underwent major change in SASA (S34C Fig). Gln21, Asn66, Asp162 of CL1 and Gly19 of Cystatin SA were enlisted in all type of interaction and as well reported high IE.

**Cystatin SN–Cathepsin L1 complex**

DSSP analysis revealed in general conservation of secondary structures in both receptor and inhibitor (S35 Fig). An initial stage fluctuation between β-bridge & β-sheet in the 1^st^ β-sheet of cystatin SN was observed in both bound and unbound state and resolved to sheet structure after 42 ns and 19 ns in unbound and bound state respectively (S35A, B Fig). 1% decrease in overall secondary structure in bound state, increase in coil structure and subsequent decrease in sheet and bend was observed in cystatin SN. The secondary structure pattern of L1 loop (residues 46-49) and the fifth β-sheet approved this observation (S35A, B Fig). CL1 exhibited an increase in secondary structure content by 1% in bound state, associated with increase in β sheet & turn and successive decrease in coil and α-helix structure (S35C, D Fig). The average backbone RMSD of CL1 and cystatin SN in bound state almost followed the unbound state throughout the simulation period but portrayed comparatively higher values (S35A Fig). Additionally, bound cystatin SN exhibited an increase in RMSD around 120ns mainly due to conformational changes in AS loop region (residues 65-73) and C-terminal end (S35A and S36A, B Fig). The complex RMSD had been stabilized ≈55ns. RMSF study also demonstrated higher fluctuation in bound state for both receptor and inhibitor molecules (S36B, C Fig).

The covariance matrix of the unbound cystatin SN was highly populated with correlated and anti-correlated motions compared to the bound inhibitor (S37A Fig) with a prevalence of correlated motions in bound state. The bound cystatin SN also appeared to be more flexible than the unbound one showed a normalized conformational overlap of 0.548 (S10 Table). Top 9 PCs of the bound inhibitor explained ≈70% of total motion, on the other hand first 16 eigenvectors of unbound cystatin SN were required for doing so. The largest PC (28.09% of total motion) in bound state described a big flipping in of AS loop towards α1 helix and minor readjustment of the conserved regions. The motion of the top eigenvector in unbound state (14.61% of total motion) corresponded to falling apart of three conserved regions assisted by reorientation of AS loop – the motion that was restricted on complex formation with CL1 (S37B Fig).

Major interactions between cystatin SN and CL1 were sketched out into S38A Fig. Electrostatic forces (69% of IE) played the major role in interaction mainly through N-terminal, which provided 67% of IE and dominated over the L1 (19 % of IE) and L2 loop (14% of IE). The β-hairpin loops were chiefly VDW contact centre. Asp137, Asp160, and Asp162 of receptor; Gly19, Tyr21, and Trp119 of the inhibitor recorded large negative potential energy of interaction and except Tyr21 and Trp119 rest acted as electrostatic interaction hub only. Gly19 and Tyr21 formed HB with three approaching Aspartic amino acid residues 137, 160 & 162 of CL1. Asn 66 of CL1 also set up favorable electrostatic interactions including HB with Gly66 & Tyr115 of the inhibitor (S38B Fig). L1 loop residues Thr64, Val65, Gly66 built favorable VDW contacts with Asn66, Gly67, Gly139, Asp162, and His63 of CL1; conserved Trp119 made VDW interaction with receptor residue Gln21; while Tyr21 participated in VDW interaction with CL1 residue Gly139 and His140. ∆SASA analysis was also well correlated with the above observations (S38A, C Fig). Asp162 of the receptor and Gly19, Tyr21, Trp119 of cystatin SN were found to take part in both electrostatic & VDW interaction.
